# A Janus heteroatom-doped carbon electrocatalyst for hydrazine oxidation

**DOI:** 10.1093/nsr/nwac231

**Published:** 2022-10-21

**Authors:** Jieting Ding, Hao-Fan Wang, Xianfeng Yang, Wenbo Ju, Kui Shen, Liyu Chen, Yingwei Li

**Affiliations:** School of Chemistry and Chemical Engineering, South China University of Technology, Guangzhou 510640, China; School of Chemistry and Chemical Engineering, South China University of Technology, Guangzhou 510640, China; Analytical and Testing Centre, South China University of Technology, Guangzhou 510640, China; School of Physics and Optoelectronics, South China University of Technology, Guangzhou 510640, China; School of Chemistry and Chemical Engineering, South China University of Technology, Guangzhou 510640, China; School of Chemistry and Chemical Engineering, South China University of Technology, Guangzhou 510640, China; School of Chemistry and Chemical Engineering, South China University of Technology, Guangzhou 510640, China

**Keywords:** electronic conductivity, heteroatom-doped carbon, hydrazine oxidation reaction, intrinsic activity, metal–organic frameworks

## Abstract

The trade-off between the intrinsic activity and electronic conductivity of carbon materials is a major barrier for electrocatalysis. We report a Janus-type carbon material combining electrically conductive nitrogen-doped carbon (NC) and catalytically active boron, nitrogen co-doped carbon (BNC). The integration of NC with BNC can not only ensure high electronic conductivity of the hybrid, but also achieve an enhancement in the intrinsic activity of the BNC side due to the electron redistribution on their coupling interfaces. In the electrocatalytic hydrazine oxidation reaction (HzOR), the Janus carbon electrocatalyst exhibits superior activity than their single counterparts and simple physical mixtures. Density functional theory calculations reveal that the NC/BNC interfaces simultaneously promote efficient electron transport and decrease the free energy of the rate-determining step in the HzOR process.

## Teaser

A Janus carbon-based structure with different heteroatom doping levels on two sides can resolve the conflict between intrinsic activity and electronic conductivity to boost the catalytic performance of carbon-based electrocatalysts in the hydrazine oxidation reaction.

## INTRODUCTION

Electrocatalysis holds a critical position in the conversion between chemical energy and electrical energy. The design of electrocatalysts with highly active centers and efficient electron conduction is crucial to achieving excellent electrocatalytic efficiency [1–3]. Carbon materials, with high electronic conductivity and tunable porous structures, are an important class of materials for electrocatalysis. Heteroatom doping is an effective strategy to regulate the electronic structures of carbon materials for enhanced electrocatalytic activities [4–7]. The heteroatom-induced charge delocalization can create highly active sites with suitable adsorption strength of reaction substrates/intermediates to facilitate the catalytic reactions [8]. However, the sp^2^ conjugated structure of carbon is disrupted in the meantime, resulting in lower electronic conductivity and thus hindering the electrocatalysis process [9–11]. Here, we reasonably envisioned that a Janus carbon-based structure with different heteroatom-doping levels between the two sides might resolve the conflict between intrinsic activity and electronic conductivity to boost the performance of carbon-based electrocatalysts.

Carbonization of carbon-containing precursors is a widely employed method for the preparation of carbon materials [12,13]. However, traditional precursors lack the designability for the synthesis of carbon materials with tunable structures and compositions. Metal–organic frameworks (MOFs), constructed using metal ions with organic linkers, are an emerging class of materials featuring high designability, tunable compositions and ordered atom distributions [14,15]. These properties have made MOFs excellent precursors for the design and fabrication of carbon-based materials with facile heteroatoms doping [16–18], tunable compositions [19–21], controllable morphologies [22–24] and well-dispersed active sites [25–29]. By carefully designing Janus MOF structures with different types or contents of heteroatoms on the two sides, it would be promising to realize the synthesis of Janus carbon materials as described above for electrocatalytic applications.

**Figure 1.**
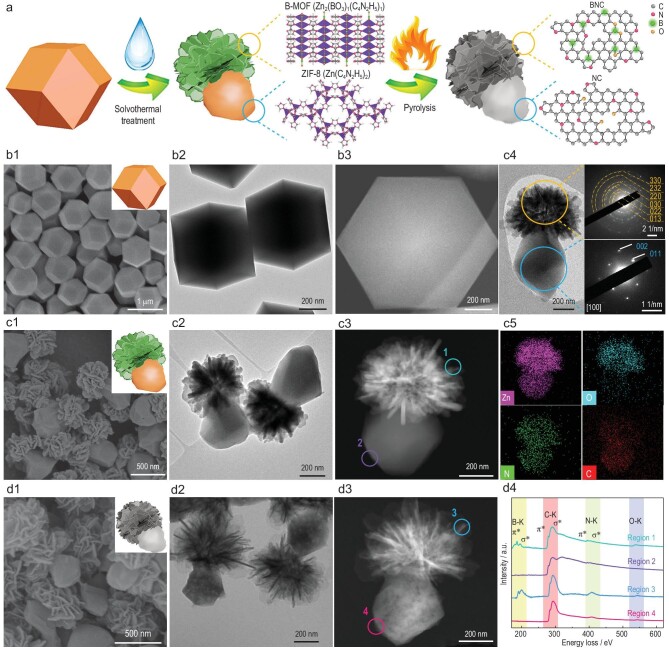
(a) Schematic synthesis process of ZIF-8/B-MOF and NC/BNC. (b1–d1) Low-magnification SEM images, (b2–d2) TEM images and (b3–d3) HAADF-STEM images of ZIF-8, ZIF-8/B-MOF and NC/BNC. (c4) TEM image of ZIF-8/B-MOF and the corresponding SAED patterns in different regions. (c5) Elemental mapping images of ZIF-8/B-MOF. (d4) EELS spectra of ZIF-8/B-MOF (Regions 1 and 2) and NC/BNC (Regions 3 and 4).

In this work, we report the realization of Janus heteroatom-doped carbon material consisting of two joint components with different compositions by creating a Janus MOF hybrid as a precursor. As a proof of concept, a Janus MOF heterostructure composed of ZIF-8 crystals and boron-containing MOF nanosheets (B-MOF) was constructed through a ‘molecular clipping and re-suturing’ process. This process enabled the partial etching of ZIF-8 for nucleation and growth of B-MOF on the etched ZIF-8, leading to the formation of ZIF-8/B-MOF with a Janus structure (Fig. 1a). The pyrolysis of ZIF-8/B-MOF yielded Janus carbon structures integrating nitrogen-doped carbon block and boron, nitrogen co-doped carbon nanosheets (denoted as NC/BNC). The NC side with a lower

doping level showed high electronic conductivity but poor intrinsic activity, while the BNC side with a higher doping level possessed high intrinsic activity but poor electron conduction. The integration of NC with BNC could not only ensure high electronic conductivity of the hybrid, but also induce further charge delocalization of active sites on the BNC side with enhanced catalytic activity. In the electrocatalytic hydrazine oxidation reaction (HzOR), NC/BNC exhibited significantly improved activity compared with the single component NC, BNC and the physical mixture NC + BNC. Experimental characterizations and theoretical calculations revealed that the NC/BNC interface could optimize the charge distribution to balance the electronic conductivity and enhance the intrinsic activity.

## RESULTS AND DISCUSSION

The ZIF-8 crystals were prepared according to the literature with some modifications [30]. The crystals exhibited a uniform rhombic dodecahedral morphology and good crystallinity with a particle size of ∼600 nm (Fig. 1b1–b3 and Supplementary Figs S1–S3). The ZIF-8 crystals were then heated in a methanol solution of boric acid (H_3_BO_3_) at 150°C for 5 h to convert the ZIF-8 into Janus ZIF-8/B-MOF.

X-ray diffraction (XRD) patterns of ZIF-8/B-MOF exhibited characteristic peaks ascribed to ZIF-8 (Zn(C_4_N_2_H_5_)_2_) and B-MOF (Zn_2_(BO_3_)_1_(C_4_N_2_H_5_)_1_) (Supplementary Fig. S4) [31]. An ^11^B solid-state nuclear magnetic resonance spectrum of ZIF-8/B-MOF showed characteristic peaks of B-MOF at 5.2, 9.3 and 13.6 ppm (Supplementary Fig. S5), confirming the formation of B-MOF. Field emission scanning electron microscopy (SEM) and transmission electron microscopy (TEM) images showed that the ZIF-8/B-MOF had a uniform pineapple-like morphology composed of a B-MOF nanosheet ‘crown’ and ZIF-8 block ‘body’ (Fig. 1c1 and c2, and Supplementary Fig. S4). Selected area electron diffraction (SAED) patterns obtained from the crown and body regions of the ZIF-8/B-MOF revealed the polycrystalline nature of B-MOF and the single-crystal nature of ZIF-8, respectively (Fig. 1c4). The high-angle annular dark-field scanning transmission electron microscopy (HAADF-STEM) image and corresponding elemental mappings showed the asymmetrically distributed compositions and Janus geometry, in which the segregation of oxygen matched well with the position of B-MOF featured with BO_3_ groups in the Janus particle (Fig. 1c3 and c5). The analysis of B distribution in the Janus particle was not successful because of the overlap between peaks of B and C in the energy dispersive X-ray spectroscopy (EDS) spectrum (Supplementary Fig. S6). Nevertheless, the electron energy loss spectra (EELS) of ZIF-8/B-MOF clearly showed the existence of B with a characteristic peak at 185 eV in the region of the B-MOF nanosheets (Fig. 1c3 and d4).

N_2_ sorption isotherms of ZIF-8/B-MOF indicated a steep increase at relatively low pressures (*P*/*P*_0_ < 0.05) and a hysteresis loop at high pressures, representing the features of microporous and mesoporous structures, respectively (Supplementary Fig. S7). Compared with the parent microporous ZIF-8 and fully converted mesoporous B-MOF, the decreased adsorption amount in the micropore region and the appearance of mesopores reflected the partial etching of ZIF-8 and the formation of B-MOF nanosheets.

To further understand the formation process of the unique ZIF-8/B-MOF structure, we carried out a time-dependent experiment and characterized the intermediate products. In the first 20 minutes, XRD patterns of the intermediate products showed identical diffractions to those of the initial ZIF-8 (Supplementary Fig. S8). From SEM and TEM images, ZIF-8 crystals were etched and evolved from a rhombic dodecahedron into a truncated rhombic dodecahedron (Supplementary Fig. S9b). At 1 h, heterogeneous nucleation of B-MOF on the vertices or edges of ZIF-8 blocks was verified by the SEM and TEM observation, with diffractions indexed to B-MOF appearing in the XRD patterns (Supplementary Figs S8 and S9c). On further prolonging the etching time, the B-MOF grew into nanosheets and assembled to a crown-like superstructure accompanied by the gradual etching of the ZIF-8 block (Supplementary Fig. S9d–f). EDS mapping images, line scan profiles, Fourier transform infrared spectroscopy (FTIR) and X-ray photoelectron spectroscopy (XPS) spectra indicated that the Zn, C and N elements dissolved from the ZIF-8 side were involved in the growth of B-MOF together with borate ions (Supplementary Figs S10–S14). After 10 h, ZIF-8 was completely transformed into B-MOF nanosheets (Supplementary Fig. S9g), in which each Zn center was coordinated by three oxygen atoms from borate ions and one nitrogen atom from the imidazole ligand (Supplementary Fig. S15).

Based on the above characterizations, we proposed a ‘molecular clipping and re-suturing’ mechanism to illustrate the formation process of ZIF-8/B-MOF. First, H_3_BO_3_ serves as a proton source to break the coordination bonds and thus liberate Zn^2+^ ions and imidazolate linkers. The etching process starts in the vertices and edges of ZIF-8 with rich metal–ligand bonds, similar to previous reports [32]. Then, the unsaturated Zn sites on the etched ZIF-8 can form the coordination bond with the released imidazolate linkers and BO_3_^3−^ (as verified by FTIR and XPS measurements), inducing the heterogeneous nucleation of B-MOF on the vertices/edges of the etched ZIF-8 (Supplementary Figs S13 and S14). Lastly, due to the lattice mismatch between B-MOF and ZIF-8 (Supplementary Fig. S15), B-MOF preferentially grows on the seeds to form a crown-like structure assembled of nanosheets on the top of ZIF-8 without covering the whole ZIF-8 surface.

In the preparation process, the temperature, solvent and acidity had important effects on the formation of ZIF-8/B-MOF. When the reaction temperature decreased to 110°C, ZIF-8 was etched to a small extent without the production of B-MOF (Supplementary Fig. S16). At 130°C, a small number of B-MOF clusters were grown on the surface of ZIF-8. At a high temperature of 170°C, most of the ZIF-8 crystals were completely converted into B-MOF. The solvent also played an important role in the formation of ZIF-8/B-MOF. If methanol was changed to deionized water, the ZIF-8 crystals were completely transformed into flower-like B-MOF nanosheets (Supplementary Fig. S17a). Conversely, the ZIF-8 particles were mostly preserved when ethanol was used as the solvent (Supplementary Fig. S17b). Therefore, the solvent with higher acidity would lead to faster etching of ZIF-8 and conversion to B-MOF. In addition, when the concentration of the H_3_BO_3_ in the methanol was increased to 30 and 40 mM, the ZIF-8 crystals were completely transformed into B-MOF with flower-like structures and detached nanosheets, respectively (Supplementary Fig. S18). These results demonstrated the importance of the temperature, solvent and concentration of H_3_BO_3_ to balance the rates of ZIF-8 etching and B-MOF growth for the formation of ZIF-8/B-MOF. For our method using ZIF-8 as both the template and precursor, the slow etching of the ZIF-8 crystals could provide a continuous supply of low-concentration Zn ions and 2-methylimidazole (2-MeIm) and create uncoordinated Zn sites to coordinate with BO_3_^3−^ for the on-site nucleation and growth of B-MOF on the etched ZIF-8. On the contrary, directly mixing the zinc acetate hexahydrate, 2-MeIm and H_3_BO_3_ in one pot only yielded separate growth of irregular ZIF-8 particles and B-MOF nanosheets (Supplementary Fig. S19), which may be due to the rapid growth of MOF at high reactant concentrations [32].

More interestingly, our strategy can be easily extended to a series of bimetallic ZIFs by simply introducing other metallic precursors. First, various bimetallic ZIF crystals were synthesized in one pot, including ZnCo–ZIF, ZnNi–ZIF and ZnCu–ZIF (see Supplementary information for details). Similarly to ZIF-8, all of the bimetallic ZIF crystals exhibited a smooth surface and uniform dodecahedral morphology (Supplementary Fig. S20). After solvothermal treatment using H_3_BO_3_, ZnM–ZIF/B-MOF (M = Co, Ni, Cu) with uniform pineapple-like morphology was obtained (Supplementary Figs S21–S24). These results demonstrated the good versatility of our strategy for the construction of Janus-type MOF heterostructures.

We further employed ZIF-8/B-MOF as the precursor for the synthesis of the Janus NC/BNC by calcination under an argon atmosphere at 950°C. During the thermal transformation, NC/BNC maintained the uniform pineapple-like morphology of ZIF-8/B-MOF (Fig. 1d1 and Supplementary Fig. S25). BNC nanosheets became thinner as compared with the parent B-MOF nanosheets, implying the shrinkage of MOF upon calcination. The NC blocks showed obvious mesopores (Fig. 1d2 and d3, and Supplementary Fig. S26), which could be generated by the enlargement of defects in the etched ZIF-8 during carbonization (as also demonstrated by the electron paramagnetic resonance results in Supplementary Fig. S27) [33]. High-resolution TEM images showed graphitized carbon layers with distorted morphology in both sides of NC/BNC (Supplementary Fig. S26). The SAED patterns mainly showed two reflections corresponding to the (002) and (100) planes of the hexagonal graphitic structures (Supplementary Fig. S26). HAADF and the corresponding elemental mappings demonstrated the asymmetrical distribution of compositions and the Janus geometry of NC/BNC (Supplementary Fig. S28). The EELS spectrum of NC/BNC revealed that the BNC nanosheets contained B, N and C elements, while the NC side only possessed C and N elements, confirming the asymmetrical distribution of compositions (Fig. 1d3 and d4, and Supplementary Fig. S29).

**Figure 2.**
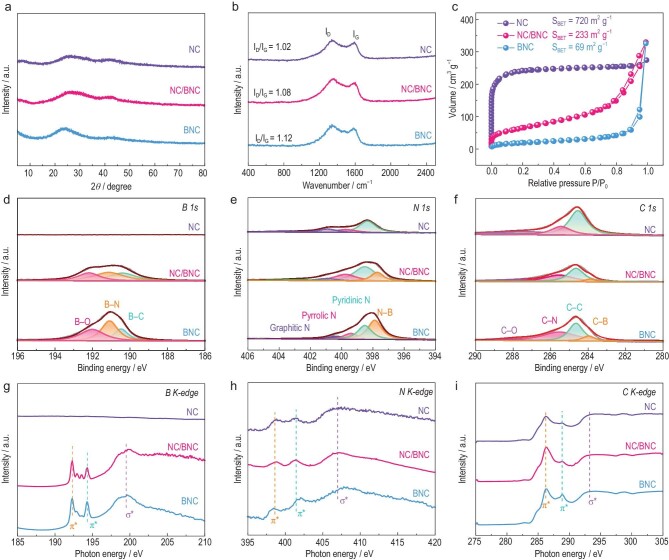
(a) XRD patterns, (b) Raman spectra, (c) N_2_ adsorption–desorption isotherms, (d) high-resolution B 1s spectra, (e) high-resolution N 1s spectra and (f) high-resolution C 1s spectra of NC, NC/BNC and BNC. XANES spectra of (g) B K-edge, (h) N K-edge and (i) C K-edge for NC, NC/BNC and BNC.

XRD patterns of NC/BNC exhibited two broad peaks at ∼24° and ∼43°, which belonged to the (002) plane and (100) plane of the graphitic carbon, respectively, indicating the complete carbonization of the MOF (Fig. 2a). The N_2_ sorption isotherm of NC/BNC showed the features of both microporous and mesoporous structures (Fig. 2c). The pore-size distribution revealed that the micropore and mesopore diameters mainly centered at ∼0.66 and ∼24 nm, respectively (Supplementary Fig. S30). The Brunauer–Emmett–Teller (BET) surface area of NC/BNC was calculated to be 233 m^2^ g^–1^ (Supplementary Table S1). The Raman spectra of NC/BNC presented an obvious D band at 1336 cm^−1^ and G band at 1584 cm^−1^, associated with the defective carbon and graphitic carbon, respectively (Fig. 2b). The N and B contents of NC/BNC were determined to be 14.1 and 6.8 wt% using elemental analysis and inductively coupled plasma atomic emission spectroscopy (ICP-AES), respectively (Supplementary Table S2).

XPS survey spectra revealed the predominant elements of C, N, B and O in NC/BNC (Supplementary Fig. S31). The N 1s spectra showed four peaks centered at 397.8, 398.6, 399.7 and 400.6 eV after deconvolution, assigned to B-bonded, pyridinic, pyrrolic and graphitic N atoms, respectively (Fig. 2e). The high-resolution B 1s spectra can be deconvoluted into three bands at 190.5, 191.1 and 192.0 eV, corresponding to B–C, B–N and B–O bonds, respectively (Fig. 2d) [34]. Based on the above analyses, it can be concluded that N and B heteroatoms have been successfully doped into the carbon matrix, as also supported by the C 1s spectra (Fig. 2f).

X-ray absorption near-edge structure (XANES) spectroscopy was further used to provide a direct probe of the bond type. In the B K-edge XANES spectra, a sharp peak at 192.3 eV corresponding to π* states of the B–C and/or B–N configuration and a peak at 194.3 eV attributed to B–O species were observed (Fig. 2g). The N K-edge XANES spectra

showed two states before the σ*-edge. The peak at 401.4 eV was assigned to the π* states of graphitic N, while the peak at 398.6 eV was attributed to pyridinic, pyrrolic and B-bonded N (Fig. 2h) [35]. The C K-edge XANES spectra exhibited three peaks at 286.2, 292.9 and 288.8 eV, assigned to the π* and σ* states of the graphitic carbon and the π* state of the heteroatom-linked carbon, respectively (Fig. 2i). The higher intensity of the graphitic carbon peak compared with the heteroatom-linked carbon peak implies that the 2D nature and periodicity of the graphitic layers are essentially unperturbed upon heteroatom doping at this level, suggesting its good electronic conductivity for electrocatalysis [36].

The Janus-type NC/BNC was then employed as the electrocatalyst for the hydrazine oxidation reaction (N_2_H_4_ + 4OH^−^ → N_2_ + 4H_2_O + 4e^−^, *E*^0^ = −0.33 V vs the reversible hydrogen electrode, RHE). The HzOR has become an emerging topic in the field of electrocatalysis in recent years for its critical role in hydrazine fuel cells and hydrazine-assisted hydrogen production [37,38]. However, the four-electron HzOR process suffers from its high overpotential. Therefore, efficient HzOR electrocatalysts are urgently needed to further improve the energy efficiency of related energy devices.

**Figure 3.**
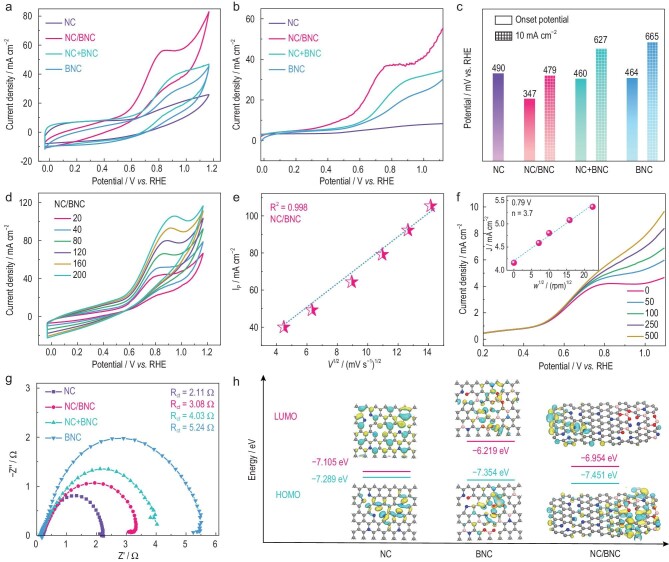
(a) CV curves of four catalysts in 1 M KOH with 0.1 M N_2_H_4_ solution at a scan rate of 50 mV s^−1^. (b) LSV curves of four catalysts in 1 M KOH with 0.1 M N_2_H_4_ solution at a scan rate of 5 mV s^−1^. (c) Histogram of onset potential and working potential to achieve 10 mA cm^−2^ for respective catalysts. (d) CVs on NC/BNC electrode in 1 M KOH with 0.1 M N_2_H_4_ solution at different scan rates and (e) plots of peak current density vs. scan rate. (f) LSV (10 mM N_2_H_4_, 1 M KOH) at different rotation speeds, analysed using a Koutecký–Levich plot at 0.79 V vs RHE. (g) EIS of samples recorded at a constant potential of 0.66 V vs RHE; (h) Highest occupied molecular orbitals (HOMO) and lowest unoccupied molecular orbitals (LUMO) of NC, BNC and NC/BNC.

The electrochemical tests were conducted via a three-electrode system with a carbon rod as the counter electrode and Hg/HgO (1 M KOH) as the reference electrode. The working electrode was fabricated by dispersing electrocatalysts on a carbon cloth substrate. The cyclic voltammetry (CV) curve showed no obvious anodic response current in the potential window without the addition of N_2_H_4_, while it gave a significant peak at 0.5 V (vs RHE, the same below if not mentioned) after the addition of N_2_H_4_ (Fig. 3a and Supplementary Fig. S32), indicating an electrocatalytic activity of N_2_H_4_ oxidation. The carbon cloth substrate exhibited a near-zero HzOR current density, excluding the contribution of the substrate on the electrocatalytic activity (Supplementary Fig. S33). Linear sweep voltammetry (LSV) tests of NC/BNC showed an onset potential of 347 mV and a working potential of 479 mV to achieve 10 mA cm^−2^ (Fig. 3b and c). Encouragingly, the onset potential of NC/BNC compared favorably to the most active carbon-based electrocatalysts reported thus far for the HzOR (Supplementary Table S3).

To understand the electrocatalysis mechanism on NC/BNC, CV tests were performed at different scan rates. The oxidation peak current density (*I*_P_) increased linearly with the square root of the scan rate (*V*^1/2^) in the range of 20–200 mV s^−1^, indicating that the oxidation of the hydrazine on NC/BNC was a diffusion-limited process (Fig. 3d and e) [39]. The electron number was determined to be 3.7 using Koutecký–Levich analysis, suggesting the near-complete oxidation of N_2_H_4_ on NC/BNC via a four-electron process to N_2_ (Fig. 3f). The evolution of N_2_ was also verified by a large number of bubbles from the electrode surface during the HzOR (Supplementary Fig. S34).

To demonstrate the advantage of the Janus NC/BNC structure, we prepared NC and BNC materials that were derived from pure ZIF-8 and B-MOF, respectively (Supplementary Figs S35 and S36). NC and BNC possessed microporous and mesoporous structures inherited from ZIF-8 and B-MOF precursors, respectively. BNC possessed higher heteroatom contents with B (14.6%) and N (19.1%) compared with that of NC (N, 10.2%), as determined using ICP-AES and elemental analysis (Supplementary Table S2). XRD patterns showed that the peak for the (002) plane of BNC shifted toward a lower diffraction angle as compared with that of NC, which may be due to the enhanced repulsive forces in the (002) plane of the carbon by the higher doping level (Fig. 2a) [40]. Raman spectra also demonstrated a higher I_D_/I_G_ value of BNC than NC, suggesting more structural defects in the carbon framework induced by heteroatom doping (Fig. 2b). The C K-edge XANES spectra of BNC showed a higher intensity of heteroatom-linked carbon peak at 288.8 eV compared with that of NC, implying the higher heteroatom-doping level of BNC (Fig. 2i).

In the HzOR, NC/BNC exhibited a lower onset potential than those of NC (490 mV) and BNC (464 mV) (Fig. 3c). Moreover, NC/BNC showed a lower working potential to achieve 10 mA cm^−2^ than BNC (665 mV), while NC cannot reach a current density of 10 mA cm^−2^ under the measured potential. As the number and intrinsic activity of accessible active sites are the two main factors to determine the electrocatalytic activity [41,42], their contributions to the activity difference in the catalytic system were then discussed.

The electrochemical active surface area (ECSA), which reflects the accessibility of active sites in electrocatalytic reactions, was determined by measuring the double-layer capacitance (*C*_dl_) of the catalysts. The *C*_dl_ values of the catalysts were in the order of NC (186.9 mF cm^−2^) > NC/BNC (127.9 mF cm^−2^) > BNC (28.7 mF cm^−2^) (Supplementary Fig. S37). The different numbers of accessible active sites in the catalysts may be related to their different pore structures, heteroatom-doping levels and electronic conductivity. BNC with abundant mesopores and higher doping levels showed lower ECSA than NC, indicating that electronic conductivity may mainly contribute to the number of accessible active sites. The charge-transfer resistances of the NC, BNC and NC/BNC electrodes were calculated to be 2.11, 5.24 and 3.08 Ω, respectively, according to the electrochemical impedance spectroscopy (EIS) results (Fig. 3g). The electronic conductivity of the samples determined using the four-point probe method also showed a trend of NC > NC/BNC > BNC, consistently with the EIS results (Supplementary Table S4). The lower electronic conductivity of BNC may be due to the co-doping of N and B with different electronegativities to break the integrity of the π-conjugated systems of graphite [43]. For NC/BNC, its electronic conductivity was improved compared with BNC due to the integration of highly conductive NC, thus increasing the accessible active sites with enhanced activity. Interestingly, a physical mixture of NC and BNC also exhibited lower charge-transfer resistances (4.03 Ω) and higher activity than BNC, confirming that NC could promote efficient electron transport for activity improvement. Furthermore, when graphene was used as the electron-transfer substrate to be mixed with BNC, the HzOR activity could be further improved as compared to NC + BNC (Supplementary Fig. S38b), indicating the importance of electron conductivity to the catalytic system.

However, the electronic conductivity alone causing the increase in the number of accessible active sites could not well explain the electrocatalytic activity difference of BNC and NC. The LSV curves were then normalized by the accessible catalytic active sites (Supplementary Figs S37 and S38a).

The normalized LSV curve showed that the intrinsic activities of the materials were in the trend of NC/BNC > BNC > NC. Compared with NC, BNC with co-doping of B and N may lead to a highly delocalized π-system for the generation of active sites with enhanced intrinsic activity. Moreover, the integration of BNC with NC for the Janus NC/BNC may lead to further electron redistribution on their coupling interfaces and thus enhanced intrinsic activity of the BNC side [44–46].

Based on the above analyses, the superior electrocatalytic activity of NC/BNC should be attributed to its unique Janus structure, which combines highly active but poorly conductive BNC and conductive but inactive NC to address the conflict between intrinsic activity and electronic conductivity. The integration of NC and BNC into a single particle led to rapid electron transfer and improved intrinsic activity and thus good electroactivity, which is superior to that of simple physical mixing.

**Figure 4.**
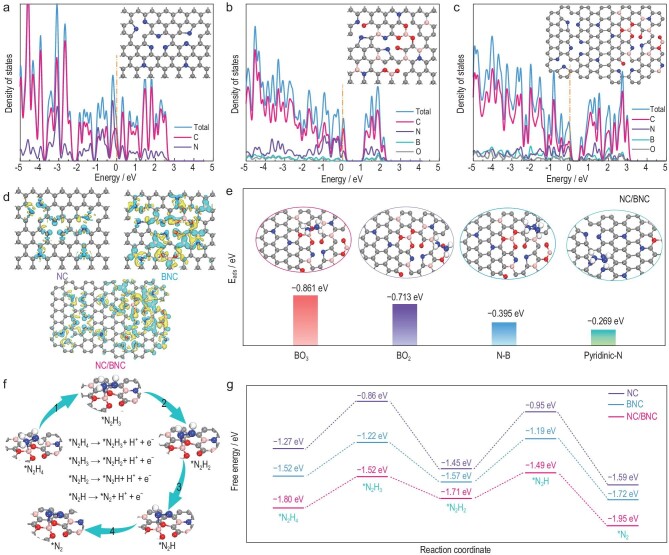
(a–c) The PDOS calculated for NC, BNC and NC/BNC. (d) The local structures and charge difference maps of NC, BNC and NC/BNC. The yellow and blue isosurfaces donate electron gain and loss, respectively. Gray, blue, pink and red spheres represent C, N, B and O atoms, respectively. (e) The adsorption energies of N_2_H_4_ on different adsorption sites of NC/BNC. (f) Optimized geometric configurations of various reaction intermediates along the reaction path of hydrazine oxidation on NC/BNC. (g) Free-energy profile for the HzOR process on NC, NC/BNC and BNC.

To uncover the possible origins of superior electrocatalytic activity of the NC/BNC heterostructure, we further performed density functional theory calculations (computational details are presented in the Supplementary information). The structure models of NC/BNC, NC and BNC were constructed based on the experimental data (Supplementary Fig. S39). The projected density-of-states (PDOS) distribution indicated that NC had a metal-like electronic-state distribution near the Fermi level (*E*_F_), while BNC and NC/BNC exhibited semiconductor properties with a discontinuous energy band near the *E*_F_ (Fig. 4a–c). However, the band gap of NC/BNC (0.50 eV) was much smaller than that of BNC (1.14 eV), suggesting a better electron transportation ability after integration of NC with BNC (Fig. 3h), consistently with the EIS results.

The charge difference distribution diagram of NC suggested its slight electronic redistribution (Fig. 4d). The Bader charges of NC indicated that C atoms adjacent to N dopants became electron-deficient with electrons transferred from the C to N atoms (Supplementary Fig. S40a). For BNC, the charge redistribution of BNC was more obvious. B atoms lose electrons accompanied by electron transfer from B to the nearest O and N atoms (Supplementary Fig. S40b). Moreover, B atoms became more positive at the NC/BNC interface due to the strong electronic affinity of N atoms (Supplementary Fig. S40c). These electropositive B and C atoms would provide strong binding sites for N_2_H_4_ adsorption. The adsorption energies (*E*_ads_) of hydrazine on different adsorption sites of NC, BNC and NC/BNC were optimized and increased in the order of pyridinic–N–C on NC (−0.378 eV) < BO_3_ on BNC (−0.521 eV) < BO_3_ on NC/BNC (−0.861 eV) (Fig. 4e and Supplementary Figs S41 and S42). These results suggested that B- and N-induced charge delocalization on the NC/BNC interface could lead to significant enhancement of N_2_H_4_ adsorption and thus contribute to the superior activity in the HzOR.

Previous studies reported that the HzOR involved four consecutive dehydrogenation steps (N_2_H_4_ → N_2_H_3_ → N_2_H_2_ → N_2_H → N_2_) [47,48]. We further investigated the stepwise dehydrogenation process of hydrazine on the surface of NC (pyridinic–N–C), BNC (BO_3_) and NC/BNC (BO_3_) (Fig. 4f and g, and Supplementary Fig. S43) to disclose the reaction mechanism. The calculation results revealed that the rate-determining step (RDS) of the HzOR on NC and BNC was the dehydrogenation from *N_2_H_2_ to *N_2_H, in which the free-energy difference for BNC (0.38 eV) was lower than that for NC (0.5 eV). More importantly, the RDS on the NC/BNC heterostructure was the formation of *N_2_H_3_ with a minimum free-energy change of 0.28 eV, which is much smaller than that of NC and BNC. The results demonstrated that the NC/BNC hybrid was more effective in facilitating the multistep dehydrogenation process, accounting for its superior electrocatalytic activity for the HzOR.

To demonstrate the advantage of MOFs as precursors for the preparation of heteroatom-doped carbon materials, we also prepared B, N-doped carbon (bulk-BNC) by using physical mixtures of Zn salt, 2-MeIm and H_3_BO_3_ as precursors. Bulk-BNC possessed similar compositions but different morphologies and fewer defects compared with BNC (Supplementary Fig. S44). Bulk-BNC showed a similar onset potential and Tafel slope to those of BNC, implying their similar intrinsic activities and thus eliminating the possible contribution of defects to the intrinsic activity [49,50]. Nevertheless, bulk-BNC exhibited lower current densities than BNC in HzOR (Supplementary Fig. S44e), verifying the advantage of MOF precursors in the synthesis of heteroatom-doped carbon materials with unique structures that increase the number of active sites. In addition, we also synthesized Zn-free B, N-doped carbon (Zn-free-bulk-BNC) without the addition of Zn salts in the preparation. Zn-free-bulk-BNC exhibited a similar N_2_H_4_ electrocatalytic activity to that of the bulk-BNC in terms of the onset potential and Tafel slope, suggesting that the electrocatalytic activity of Zn toward the HzOR is negligible (Supplementary Fig. S44).

The stability of NC/BNC was also investigated using CV cycling in 0.1 M N_2_H_4_. After 3000 cycles, the current value on NC/BNC decreased only slightly (Supplementary Fig. S45). The morphology and chemical states of NC/BNC were well preserved after the cycling test, demonstrating the excellent stability of NC/BNC (Supplementary Figs S46–S48).

Inspired by the excellent HzOR performances of NC/BNC, we further evaluated its potential application for electrocatalytic hydrogen production based on overall hydrazine splitting (OHzS) in a two-electrode electrolyser (Pt/C + NC/BNC) (Supplementary Fig. S49a). The comparing LSV curves of OHzS (1.0 M KOH/0.1 M N_2_H_4_) and overall water splitting (OWS, 1.0 M KOH) illustrated the significantly enhanced energy efficiency using HzOR to assist H_2_ production (Supplementary Fig. S49b). Specifically, the OHzS system only required cell voltages of 0.29, 0.61 and 0.90 V to reach current densities of 20, 50 and 100 mA cm^−2^, respectively (Supplementary Fig. S49c). For comparison, much higher voltages of 1.53, 1.90 and 2.60 V were required for the OWS system to obtain the above current densities, respectively. The OHzS system could maintain a cell voltage of 0.5 V with acceptable stability considering the hydrazine consumption during the 30 h of continuous testing (Supplementary Fig. S49d).

## CONCLUSIONS

In summary, we have proposed a ‘molecular clipping and re-suturing’ process to construct Janus-type MOF heterostructures that could be further

thermally transformed into Janus NC/BNC electrocatalysts for the hydrazine oxidation reaction. The B and N heteroatom-induced charge delocalization in BNC contributes to the high intrinsic HzOR activity, while the N-doped NC ensures high electronic conductivity and induces further charge delocalization to regulate the electronic structure of the active sites near the NC–BNC interface for improved HzOR activity. Benefitting from the unique Janus structure, NC/BNC exhibited a low HzOR onset potential of 347 mV and a working potential of 479 mV to achieve 10 mA cm^−2^ in the electrolyte of 1 M KOH with 0.1 M N_2_H_4_. This work not only provides a new strategy for the synthesis of Janus-type MOFs and MOF-derived materials, but also offers insights into the design of heteroatom-doped carbon materials for efficient electrocatalysis.

## MATERIALS AND METHODS

Detailed materials and methods are available in the Supplementary data.

## Supplementary Material

nwac231_Supplemental_File
