# Supplementary material for: A Janus heteroatom-doped carbon electrocatalyst for hydrazine oxidation
Source: Natl Sci Rev. 2022 Oct 21;10(3):nwac231. doi: 10.1093/nsr/nwac231 (PMC10084918; doi:10.1093/nsr/nwac231)
Supplement: nwac231_Supplemental_File [file nwac231_supplemental_file.doc]

**Supplementary Information**

**A Janus heteroatom-doped carbon electrocatalyst for hydrazine oxidation**

Jieting Ding,^1^ Hao-Fan Wang,^1^ Xianfeng Yang,^2^ Wenbo Ju,^3^ Kui Shen,^1^ Liyu Chen,^1*^ Yingwei Li^1*^

^1^ School of Chemistry and Chemical Engineering, South China University of Technology, Guangzhou 510640, China.

^2^ Analytical and Testing Centre, South China University of Technology, Guangzhou 510640, China.

^3^ School of Physics and Optoelectronics, South China University of Technology, Guangzhou 510640, China.

*Corresponding author. liyuchen@scut.edu.cn; [liyw@scut.edu.cn](mailto:liyw@scut.edu.cn)

**Content**

**1. Experimental Section** 4

**1.1 Chemicals** 4

**1.2 Materials preparation** 4

**1.3 Characterizations** 6

**1.4 Electrochemical measurements** 7

**1.5 DFT calculations** 9

**2.** **XRD and Raman characterizations of ZIF-8** 10

**3. XPS characterizations of ZIF-8** 11

**4.** **Microstructure of ZIF-8** 12

**5.** **Characterizations and microstructure of ZIF-8, ZIF-8/B-MOF and B-MOF** 13

**6.** **^11^B SSNMR spectra** **of ZIF-8/B-MOF and B-MOF** 14

**7.** **EDS spectra of ZIF-8/B-MOF** 15

**8.** **N_2_ sorption** 16

**9.** **XRD patterns** 17

**10. SEM and TEM images** 18

**11. HAADF-STEM and elemental mapping images** 19

**12. Elemental mapping images and elemental line scan profiles of Zn, O** 20

**13. Elemental mapping images and elemental line scan profiles of C, O** 21

**14. FT-IR spectrum** 22

**15. XPS spectra** 23

**16. Crystal structure** 24

**17. SEM images of ZIF-8 in a methanol solution of H_3_BO_3_ at different temperatures** 25

**18. SEM images of ZIF-8 in different solutions of H_3_BO_3_** 26

**19. SEM images of ZIF-8 in methanol solution with different concentrations of H_3_BO_3_** 27

**20. Characterizations and microstructure of the control sample synthesized in one pot** 28

**21. SEM images of different bimetallic ZIFs** 29

**22. SEM images** **of different bimetallic ZnM-ZIF/B-MOF heterostructures (M = Co, Ni, Cu)** 30

**23. XRD characterizations of ZnM-ZIF/B-MOF heterostructures (M = Co, Ni, Cu)** 31

**24. XPS characterizations of ZnM-ZIF/B-MOF heterostructures (M = Co, Ni, Cu)** 32

**25. HAADF-STEM and elemental mapping images** **of ZnM-ZIF/B-MOF heterostructures (M = Co, Ni, Cu)** 33

**26. SEM images of NC/BNC** 34

**27. Microstructure of NC/BNC** 35

**28. EPR spectra of ZIF-8, ZIF-8/B-MOF, and B-MOF** 36

**29. HAADF-STEM and elemental mapping images of NC/BNC** 37

**30. XPS and EELS characterizations of NC derived from ZIF-8-110** 38

**31. Pore size distribution of NC, NC/BNC, and BNC** 39

**32. Survey XPS spectra of NC, NC/BNC and BNC** 40

**33. Electrocatalytic N_2_H_4_ performance** 41

**34. CV curves of NC/BNC and carbon cloth electrodes** 42

**35. Photograph of NC/BNC** 43

**36. Microstructure of NC** 44

**37. Microstructure of BNC** 45

**38. Electrochemical double-layer capacitance measurement** 46

**39. LSV polarization curves** 47

**40. Atomic structure models** 48

**41. Bader charges** 49

**42. The adsorption energies of N_2_H_4_ on different adsorption sites of NC** 50

**43. The adsorption energies of N_2_H_4_ on different adsorption sites of BNC** 51

**44. Geometric configurations** 52

**45. Electrocatalytic N_2_H_4_ performance** 53

**46. Stability of NC/BNC electrodes** 54

**47. Microstructure of NC/BNC after the stability test** 55

**48. XRD and XPS spectra of NC/BNC after the stability test** 56

**49. XANES spectra of NC/BNC after the stability test** 57

**50. Electrocatalytic OHzS performance** 58

**51. Characterization results of catalysts** 59

**52. ICP-AES results for Zn and B, and elemental analysis data for C, N and H in NC, NC/BNC and BNC** 60

**53. Comparison of the HzOR performances of NC/BNC with various reported electrocatalysts** 61

**54. Conductivity tests of the catalysts** 62

**55. References** 63

**1. Experimental Section**

**1.1 Chemicals**

All chemicals were purchased from commercial sources and used without further treatment. Cobalt(II) acetate tetrahydrate (Co(AC)_2_∙4H_2_O, 99%, Aladdin Industrial Corporation), zinc acetate dihydrate (Zn(AC)_2_∙2H_2_O, 99%, Aladdin Industrial Corporation), copper(II) acetate monohydrate (Cu(AC)_2_∙H_2_O, 99%, Aladdin Industrial Corporation), nickel(II) acetate tetrahydrate (Ni(AC)_2_∙4H_2_O, 99%, Aladdin Industrial Corporation), 2-methylimidazole (2-MeIm, C_4_H_6_N_2_, 98%, Aladdin Industrial Corporation), boric acid (H_3_BO_3_, 99%, Tianjin Damao Chemical Reagent Factory), potassium hydroxide (KOH, 99%, Aladdin Industrial Corporation), methanol (CH_3_OH, ≥ 99.7%, Guangdong Guanghua Sci-Tech Co., Ltd.), and hydrazinium hydrate (N_2_H_4_∙H_2_O, 50%, Aladdin Industrial Corporation).

**1.2 Materials preparation**

**Preparation of ZIF-8.** First, 5 mL of 2-MeIm aqueous solution (13.64 mM) was rapidly added to 5 mL of Zn(AC)_2_∙2H_2_O aqueous solution (1.36 mM). After stirring for 10 s, the aqueous solution was left at room temperature for 24 h. Finally, the obtained sample was washed with methanol and dried in an oven at 60 °C.

**Preparation of** **ZnM-ZIF (M = Co, Ni, Cu).** Bimetallic ZnCo-ZIF was prepared by the same procedure as that of ZIF-8 except that 5 mL of Zn(AC)_2_∙2H_2_O (1.16 mM) and Co(AC)_2_∙4H_2_O (0.2 mM) aqueous solution instead of 5 mL of Zn(AC)_2_∙2H_2_O aqueous solution (1.36 mM) was used. Bimetallic ZnNi-ZIF was prepared by the same procedure as that of ZIF-8 except that 5 mL of Zn(AC)_2_∙2H_2_O (1.16 mM) and Ni(AC)_2_∙4H_2_O (0.2 mM) aqueous solution instead of 5 mL of Zn(AC)_2_∙2H_2_O aqueous solution (1.36 mM) was used. Bimetallic ZnCu-ZIF was prepared by the same procedure as that of ZIF-8 except that 5 mL of Zn(AC)_2_∙2H_2_O (1.16 mM) and Cu(AC)_2_∙4H_2_O (0.2 mM) aqueous solution instead of 5 mL of Zn(AC)_2_∙2H_2_O aqueous solution (1.36 mM) was used.

**Preparation of ZIF-8/B-MOF.** The as-prepared ZIF-8 was dispersed in 30 mL of methanol, and then 30 mL of H_3_BO_3_ methanol solution (20 mM) was added. After stirring for 15 minutes, the mixture was transferred into a 100 mL stainless steel autoclave lined with Teflon and heated at 150 ℃ for 5 h to obtain ZIF-8/B-MOF.

**Preparation of B-MOF.** The as-prepared ZIF-8 was dispersed in 30 mL of methanol, and then 30 mL of H_3_BO_3_ methanol solution (20 mM) was added. After stirring for 15 minutes, the mixture was transferred into a 100 mL stainless steel autoclave lined with Teflon and heated at 150 ℃ for 10 h to yield B-MOF.

**Preparation of ZnM-ZIF/B-MOFs (M = Co, Ni, Cu).** A series of bimetallic ZnM-ZIF/B-MOF hybrids were also prepared by the same procedure as that of ZIF-8/B-MOF except that ZnM-ZIFs instead of ZIF-8 were used.

**Preparation of NC, BNC and NC/BNC.** The dried ZIF-8, B-MOF and ZIIF-8/B-MOF samples were calcined at 950 ºC for 2 h with a heating rate of 2.5 °C·min^−1^ in argon atmosphere. The obtained materials derived from ZIF-8, ZIF-8/B-MOF, and B-MOF were denoted as NC, NC/BNC, and BNC, respectively.

**Preparation of bulk-BNC.** 0.30 g of Zn(AC)_2_∙2H_2_O, 1.12 g of 2-MeIM and 1.24 g of H_3_BO_3_ were directly mixed and subsequently calcined at 950 °C for 2 h with a heating rate of 2.5 °C min^−1^ under argon atmosphere to obtain the bulk-BNC material.

**Preparation of Zn-free-bulk-BNC.** 1.12 g of 2-MeIM and 1.24 g of H_3_BO_3_ were directly mixed, then calcined at 550 ºC for 2 h with a heating rate of 2.5 °C·min^−1^ in argon atmosphere to obtain the Zn-free-bulk-BNC material.

**1.3 Characterizations**

X-ray diffraction (XRD) patterns were recorded on a Bruker D8 ADVANCE, using filtered Cu-Kα radiation (λ = 0.1543 nm) generated at 40 kV and 40 mA. Scans for 2*θ* values were recorded at 4° min^‒1^ between 5° and 80°. Raman spectra were obtained on the HJY LabRAM Aramis Raman spectrometer (HORIBA Jobin Yvon) at room temperature. The structure and morphology of various samples were determined on a high-resolution field-emission scanning electronic microscopy (SEM, Hitachi SU8220). Transmission electron microscopy (TEM), EDX analysis (Bruker XFlash 5030T) and high angle annular dark field scanning transmission electron microscopy (HAADF-STEM) images were taken on a JEOL (JEM-2100 F) microscope operating at 200 kV. X-ray photoelectron spectroscopy (XPS) was obtained by the Thermo Scientific NEXSA spectrometer with a Mono AlKa (1486.6 eV, 15 KV, 15 mA X-ray source). The N2 adsorption/desorption isotherms were measured by a Micromeritics ASAP 2020M instrument at 77 K. High resolution solid-state nuclear magnetic resonance (NMR) spectra were recorded at ambient temperature on an Agilent dsx-300 spectrometer using a standard Agilent magic angle rotation (MAS) probe with a 4 mm (outer diameter) zirconia rotor. The metal and B element contents of the samples were determined by inductively coupled plasma atomic emission spectroscopy (ICP-OES) on an Agilent 720ES instrument. The contents of C, H and N in the samples were determined on a German elementar Vario EL cube. The X-ray absorption near-edge structure (XANES) spectroscopy data of the samples were recorded at room temperature in transmission mode and in the fluorescent mode with silicon drift fluorescence detector at beam line BL08U1A of the Shanghai Synchrotron Radiation Facility (SSRF), China.

**1.4 Electrochemical measurements**

First, 31.17g of KOH was dissolved in 500 mL of ultrapure water to obtain 1 M KOH electrolyte. Subsequently, the electrolyte was purified by electrolysis between two graphite rods at 0.1 mA for 24 hours to remove traces of metal ions. Electrochemical performance tests were performed using an electrochemical workstation (CHI 760E, Shanghai Chenhua Ltd., China) and Neware battery system (CT3008-5V10mA-164, Shenzhen, China). A conventional three electrode system with carbon rods as counter electrode, Hg/HgO (1 M KOH) electrode as reference electrode, and carbon cloth supported catalyst as working electrode (carbon cloth exposed geometric area was 1 × 1 cm^2^; the loading mass of NC, BNC or NC/BNC was 2.05 ± 0.03 mg) was used. All the measured potentials (vs. the Hg/HgO reference electrode) in this work were converted to the reversible hydrogen electrode (RHE) by using the following equation:

E_(RHE)_ = E_(Hg/HgO)_ + 0.059 pH + 0.14

No iR compensation was used for all electrochemical measurements.

Koutecký–Levich analysis was conducted via a conventional three electrode system with carbon rods as counter electrode, Hg/HgO (1 M KOH) electrode as reference electrode, and catalyst-coated glassy carbon electrode (GCE) as working electrode. For the catalyst ink, 2.0 mg of the catalyst was dispersed in a mixture of 190 µL of ethanol and 10 µL of Nafion solution and sonicated for 20 min. 10 µL of the ink was deposited on GCE (A = 0.1962 cm^2^) and dried in air. To calculate the number of electrons involved in the oxidation processes, we were performed linear sweep voltammetry (LSV) on a rotating disk electrode (RDE) in the presence of 10 mM hydrazine in 1 M KOH.

The number of electrons recovered for N_2_H_4_ oxidation at the NC/BNC was determined using the Levich equation:

*i_l_* = 0.62nFAD_R_^2/3^ ω^1/2^ ν^−1/6^ C_R_*

where *i_l_* is the limiting current at a given potential, n is the number of electrons involved in the reaction, F is Faraday’s constant (96500 C/mol), A is the electrode surface area (0.1962 cm^2^), D_R_ is the diffusion coefficient of the hydrazine (1.40 × 10^−5^ cm^2^·s^−1^), ω is the rotation rate, ν is the solution’s kinematic viscosity (1 M KOH, 1.08 × 10^−2^ cm^2^·s^−1^), and C_R_* is the concentration of the reduced species (10 mM N_2_H_4_). To eliminate error from background currents and verify transport-limited behavior, values for n were determined from the slopes of linear regions of Levich plots (*i_l_* vs ω^1/2^).

The electrochemical active surface area (ECSA) was estimated by measuring the capacitive current associated with double-layer charging from the scan-rate dependence of cyclic voltammograms (CVs).

**1.5 DFT calculations**

We employed the Vienna Ab Initio Package (VASP) to perform all the density functional theory (DFT) calculations within the generalized gradient approximation (GGA) using the PBE formulation^S1–S3^. We chose the projected augmented wave (PAW) potentials to describe the ionic cores and take valence electrons into account using a plane wave basis set with a kinetic energy cutoff of 450 eV^S4, S5^. Partial occupancies of the Kohn−Sham orbitals were allowed using the Gaussian smearing method and a width of 0.05 eV. The electronic energy was considered self-consistent when the energy change was smaller than 10^−4^ eV. A geometry optimization was considered convergent when the force change was smaller than 0.05 eV/Å. Grimme’s DFT-D3 methodology was used to describe the dispersion interactions^S6^.

The equilibrium lattice constants of Graphene unit cell were optimized, when using a 10 × 10 × 4 Monkhorst-Pack k-point grid for Brillouin zone sampling. We then used it to construct a supercell Graphene model in the x and y directions. During structural optimizations, the 3 × 3 × 1 Monkhorst-Pack k-point grid for Brillouin zone was used for k-point sampling for the N doping, N, B and O atoms doping in the supercell graphene structure. Finally, the adsorption energies (E_ads_) were calculated as E_ads_ = E_ad/sub_ − E_ad_ − E_sub_, where E_ad/sub_, E_ad_, and E_sub_ are the total energies of the optimized adsorbate/substrate system, the adsorbate in the structure, and the clean substrate, respectively.

**2.** **XRD and Raman characterizations of ZIF-8**


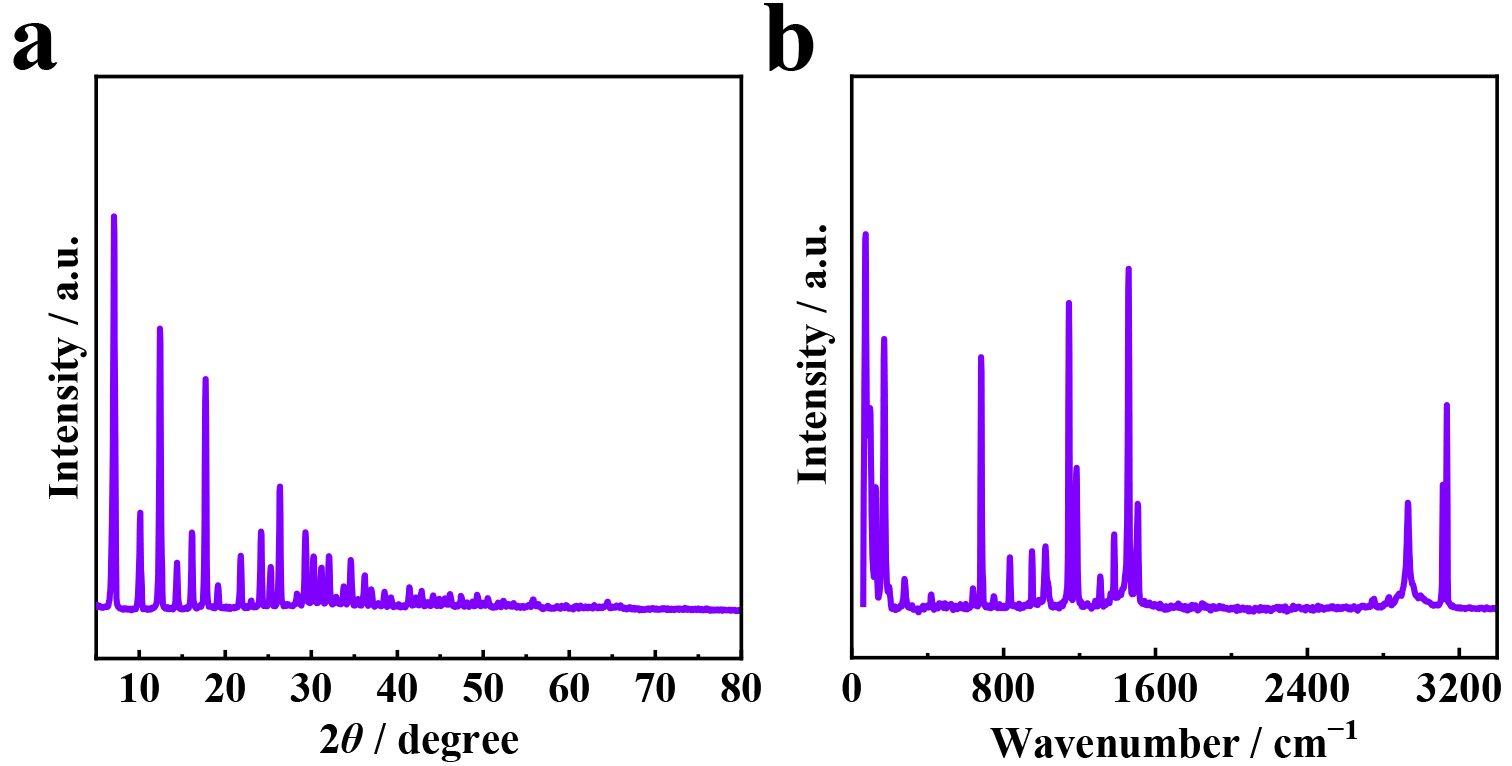


**Figure S1.** XRD and Raman characterizations. a, XRD patterns of ZIF-8. b, Raman spectra of ZIF-8.

**3.** **XPS characterizations of ZIF-8**


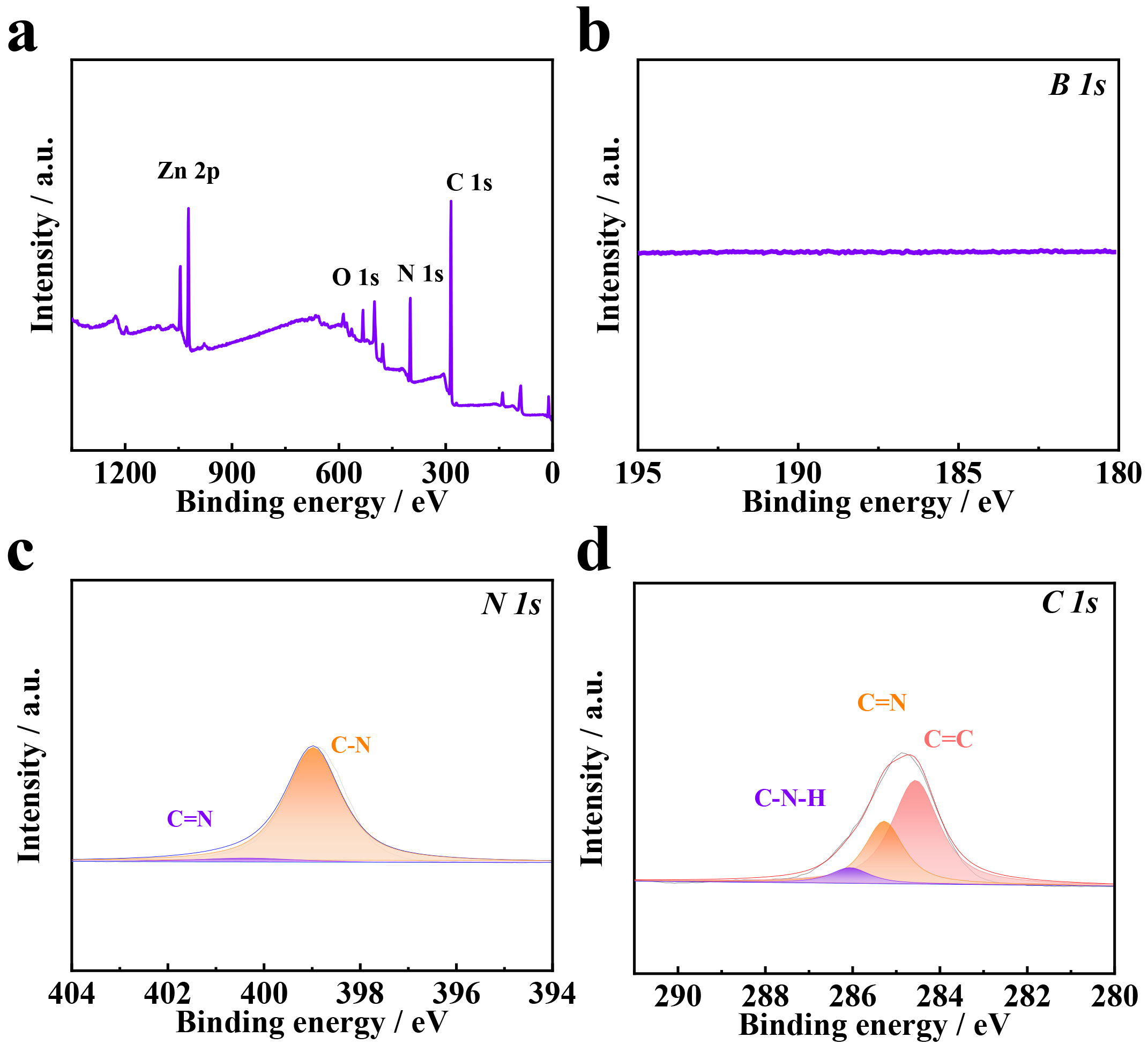


**Figure S2.** XPS characterizations. a–d, XPS survey spectra (a), high-resolution B 1s spectra (b), high-resolution N 1s spectra (c), and high-resolution C 1s spectra (d) of ZIF-8.

**4.** **Microstructure of ZIF-8**


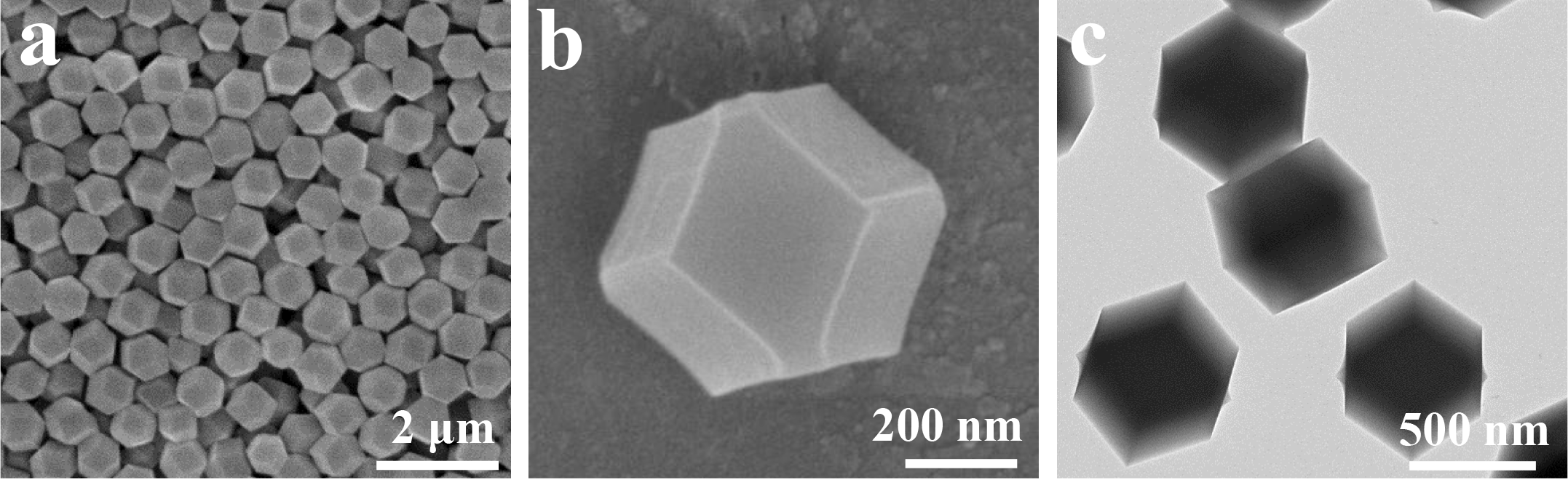


**Figure S3.** Microstructure of ZIF-8. a, b, SEM images. c, TEM image.

**5.** **Characterizations and microstructure of ZIF-8, ZIF-8/B-MOF and B-MOF**


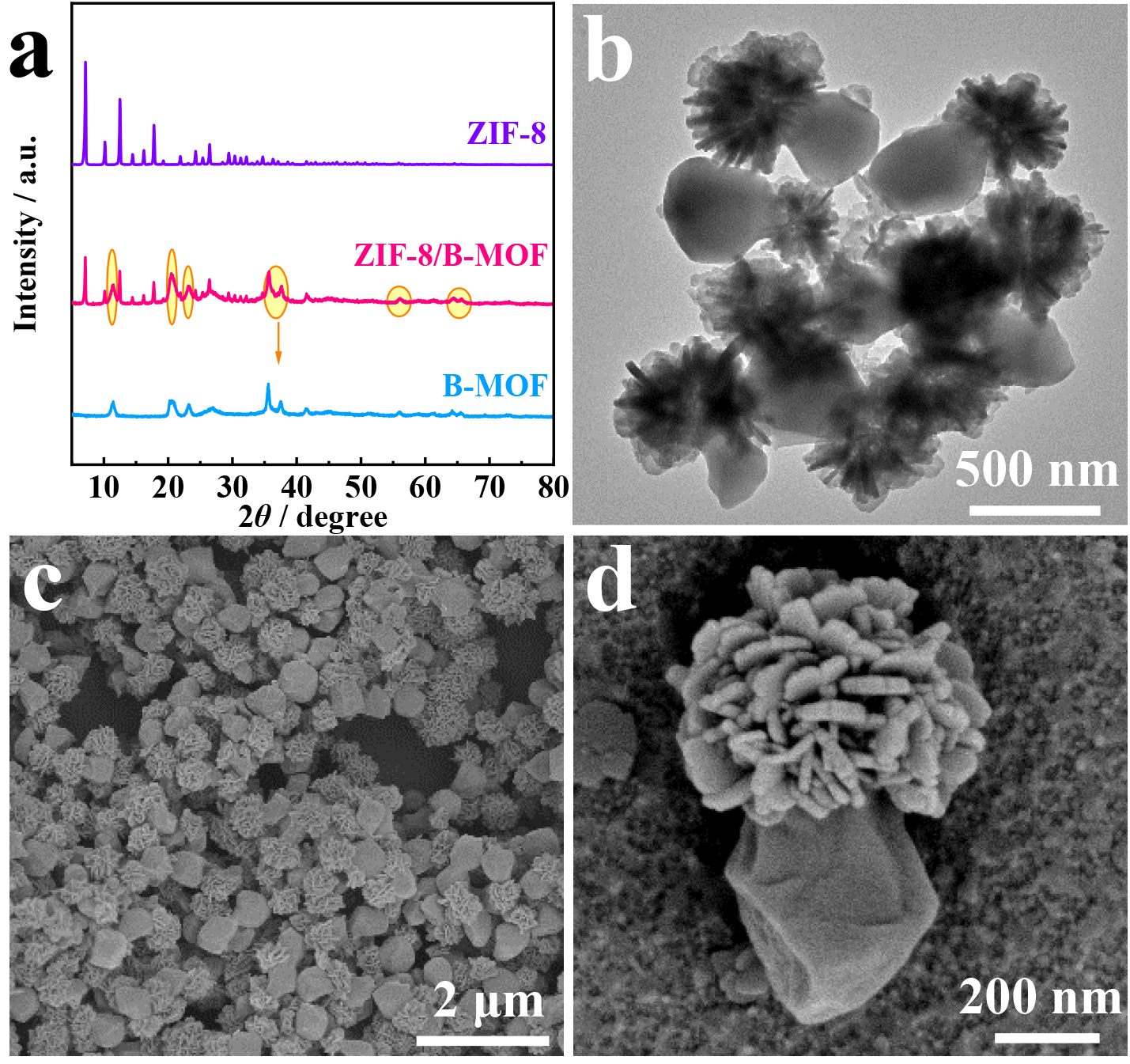


**Figure S4.** Characterizations and microstructure of ZIF-8, ZIF-8/B-MOF and B-MOF. a, XRD pattern of ZIF-8, ZIF-8/B-MOF and B-MOF. b, TEM image of ZIF-8/B-MOF. c, d, SEM images of ZIF-8/B-MOF.

**6.** **^11^B SSNMR spectra** **of ZIF-8/B-MOF and B-MOF**


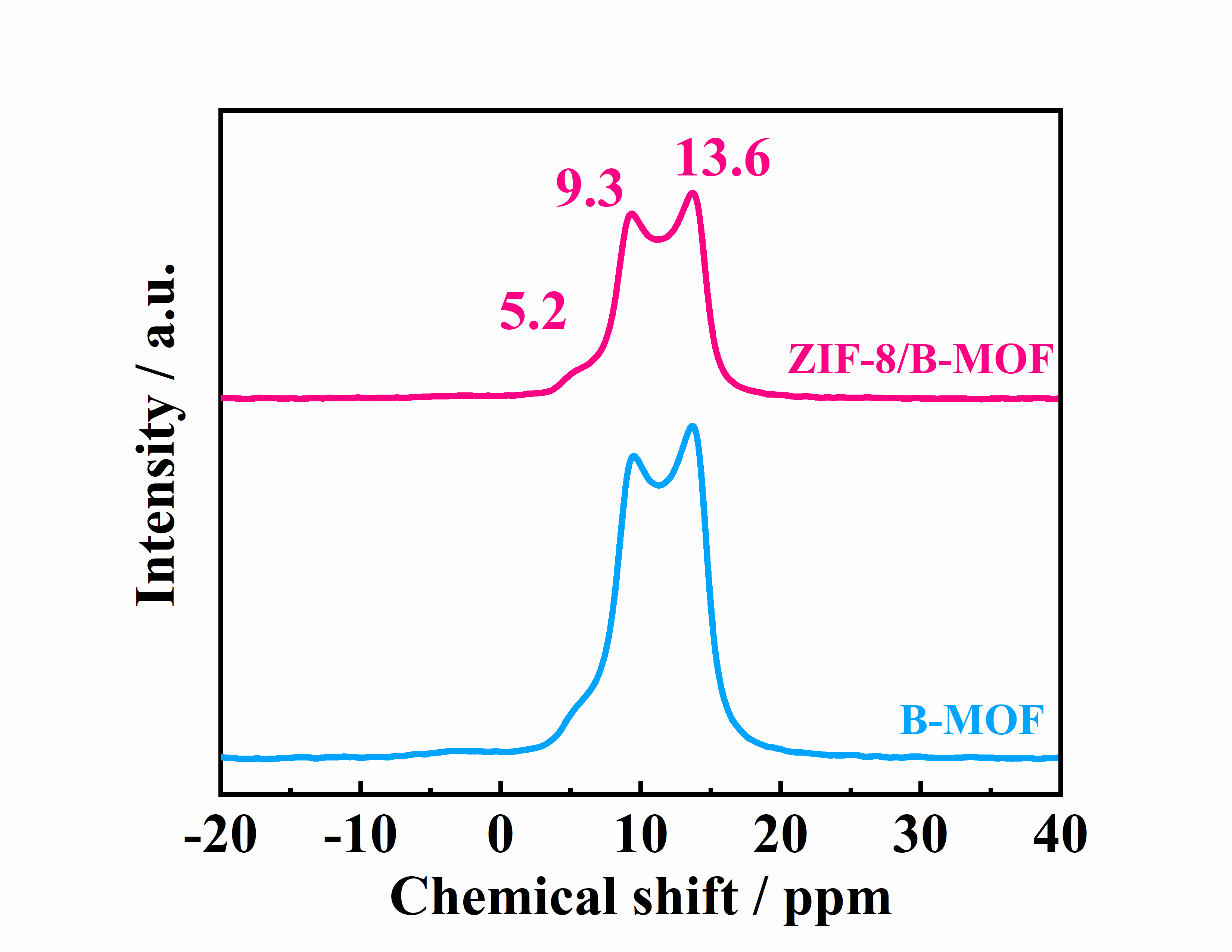


**Figure S5.** ^11^B SSNMR spectra of ZIF-8/B-MOF and B-MOF.

**7.** **EDS spectra of ZIF-8/B-MOF**

**
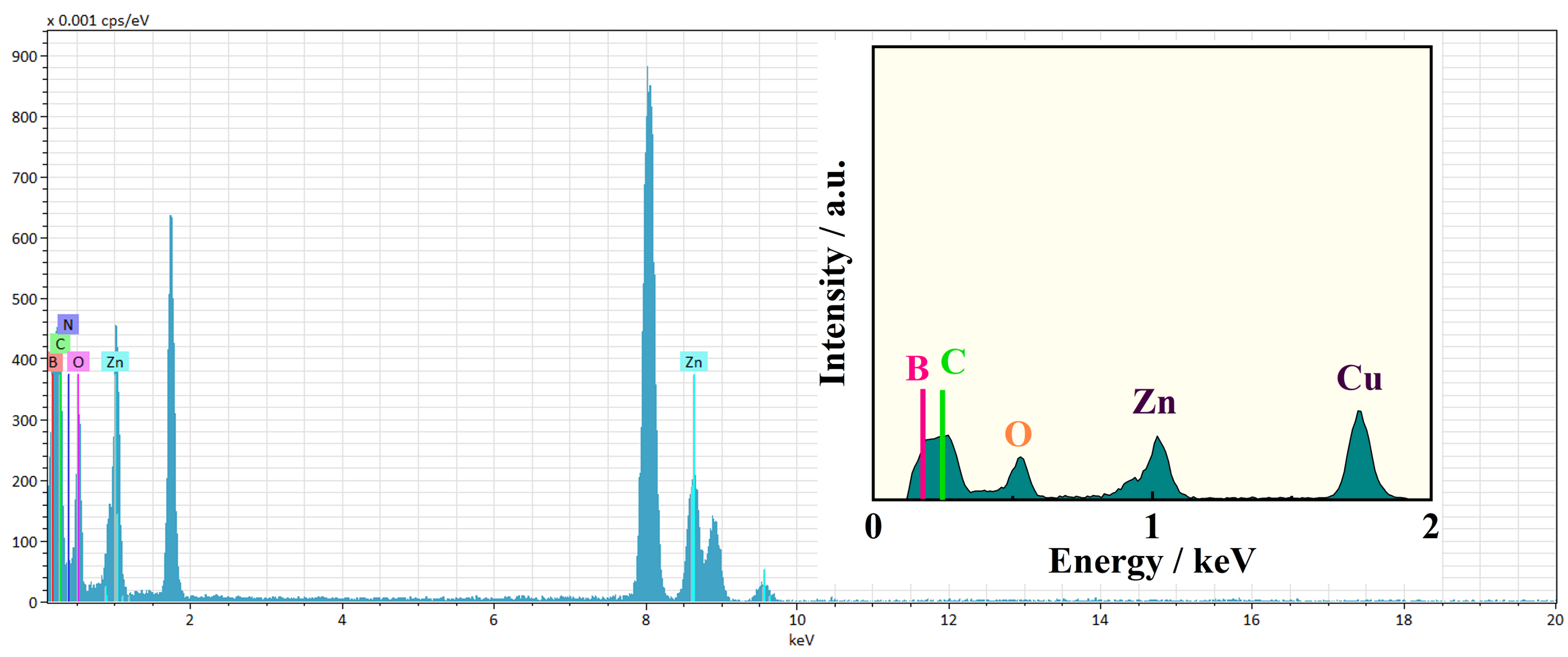
**

**Figure S6.** EDS spectra of ZIF-8/B-MOF.

**8.** **N_2_ sorption**


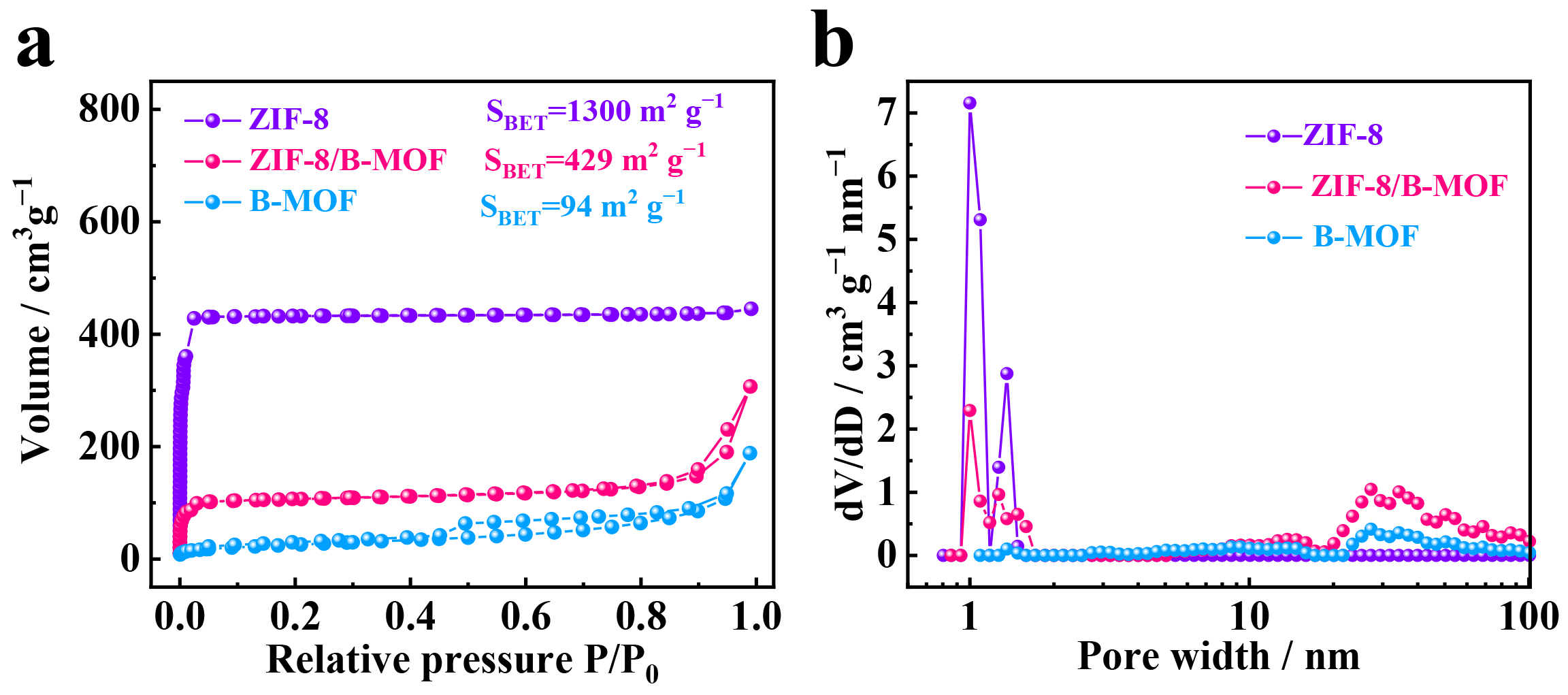


**Figure S7.** Characterizations of NC, NC/BNC and BNC. a, b, Nitrogen adsorption-desorption isotherm (a) and the corresponding pore size distribution (b) of ZIF-8, ZIF-8/B-MOF and B-MOF.

**9.** **XRD patterns**


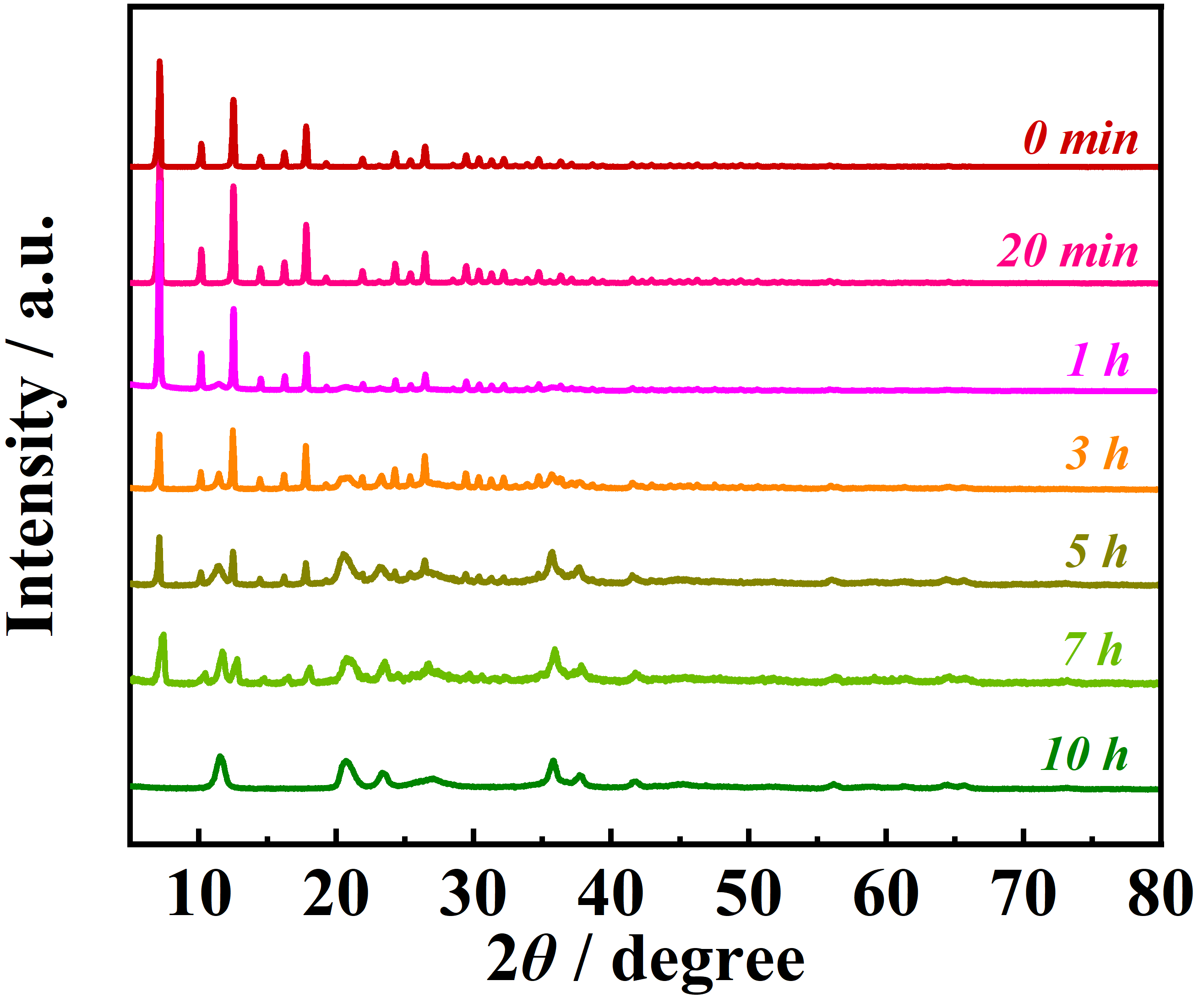


**Figure S8.** XRD patterns of intermediate products yielded at different reaction time.

**10. SEM and TEM images**


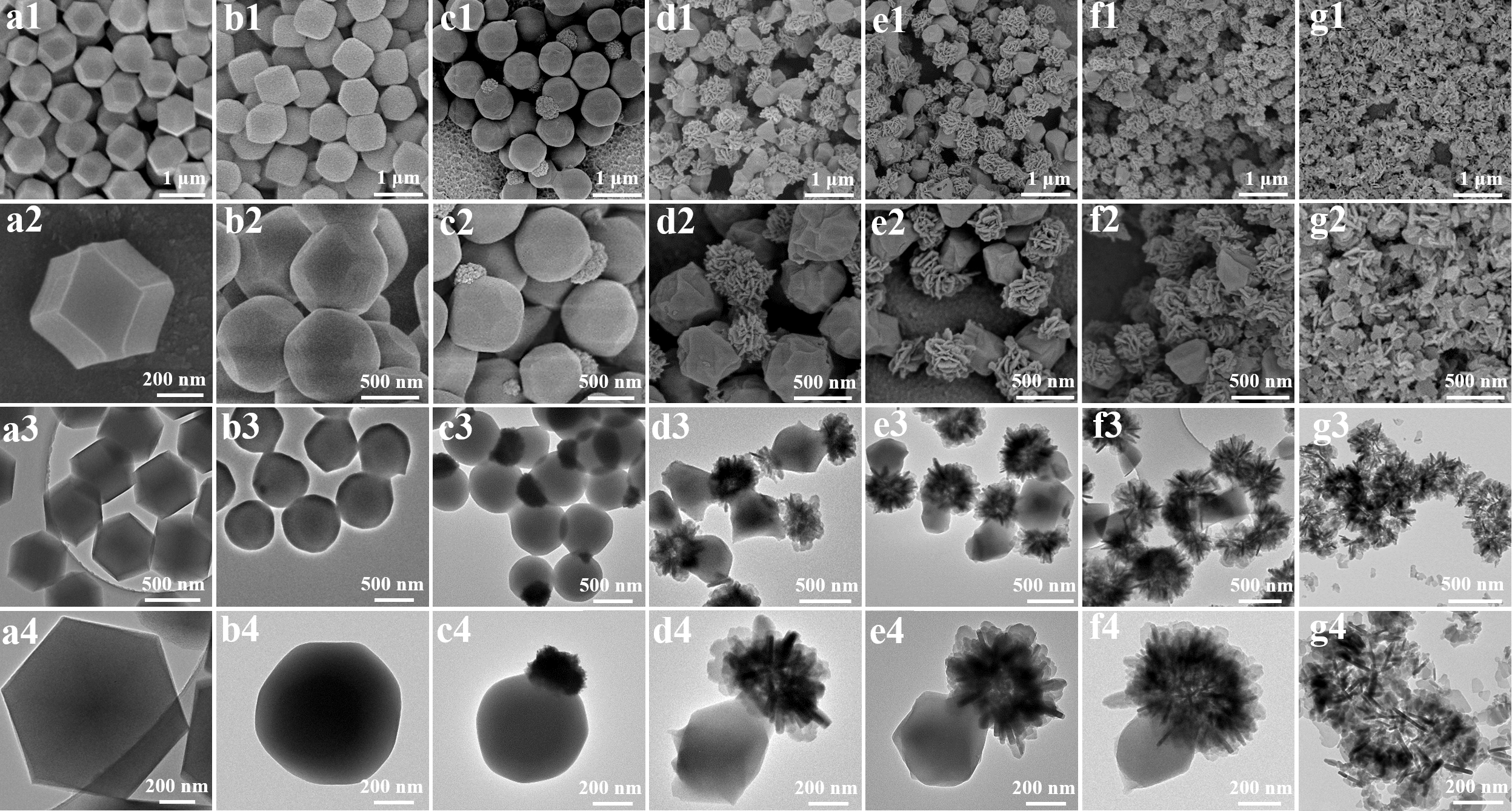


**Figure S9.** Structural transformation of ZIF-8 to ZIF-8/B-MOF to B-MOF. a1–g1, a2–g2, SEM images, a3–g3, a4–g4, TEM images of the intermediate products prepared with different reaction time at 0 min (a), 20 min (b), 1 h (c), 3 h (d), 5 h (e), 7 h (f) and 10 h (g).

1. **HAADF-STEM and elemental mapping images**


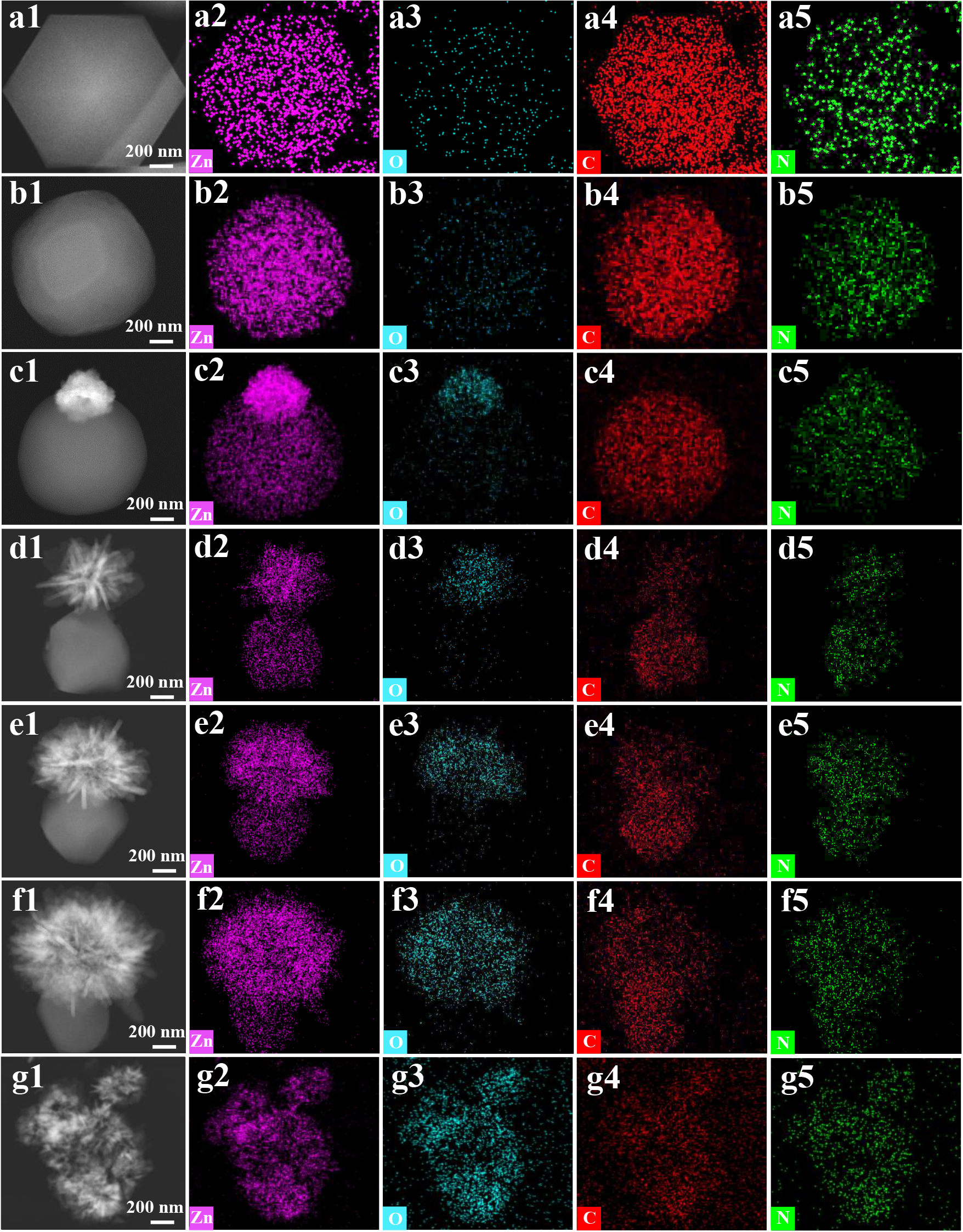


**Figure S10.** Structural transformation of ZIF-8 to ZIF-8/B-MOF to B-MOF. a1–g1, HAADF-STEM, a2–g2, a3–g3, a4–g4, a5–g5, elemental mapping images of the intermediate products prepared with different reaction time at 0 min (a), 20 min (b), 1 h (c), 3 h (d), 5 h (e), 7 h (f) and 10 h (g).

1. **Elemental mapping images and elemental line scan profiles of Zn, O**


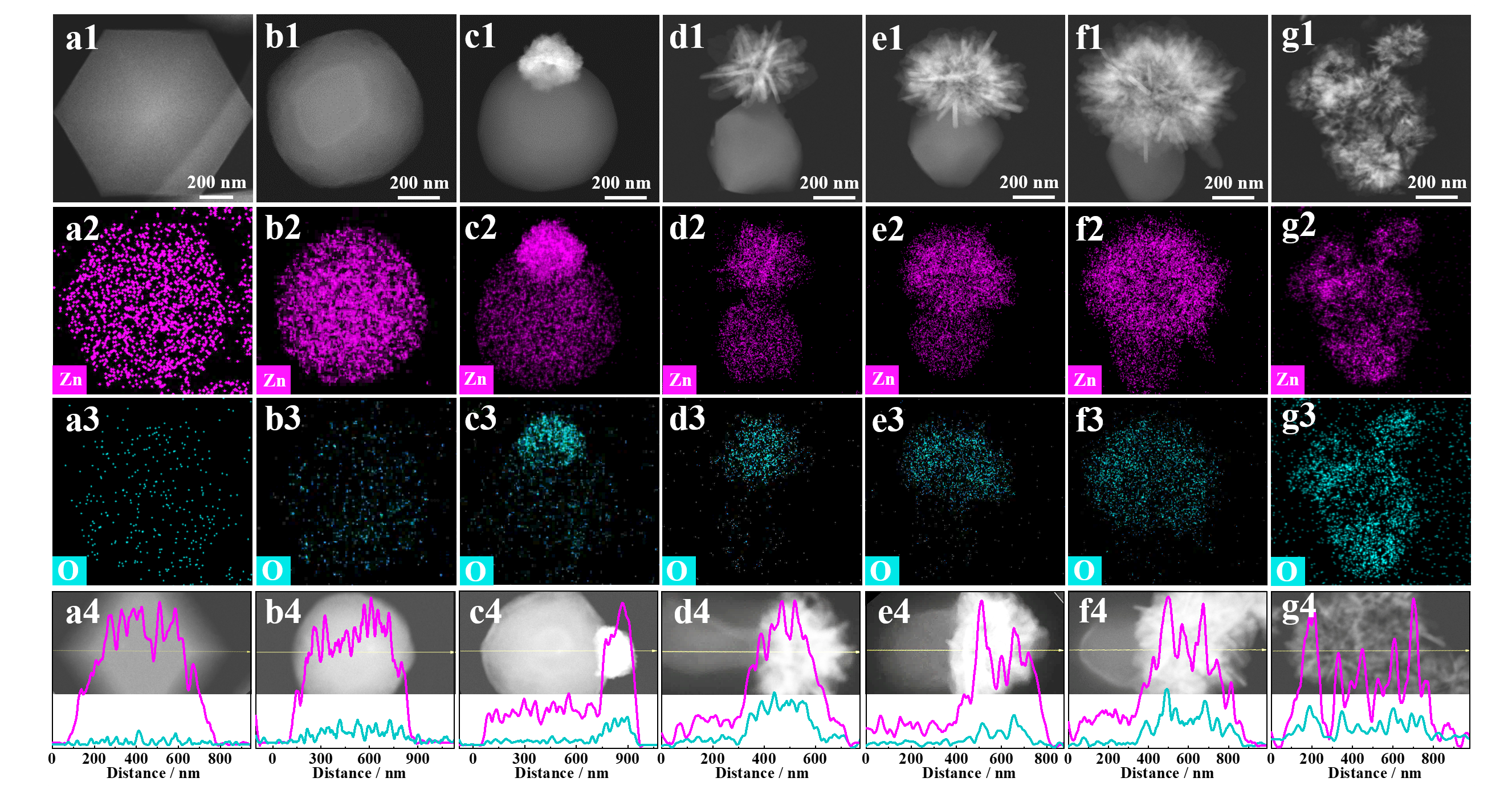


**Figure S11.** Structural transformation of ZIF-8 to ZIF-8/B-MOF to B-MOF. a1–g1, HAADF-STEM, a2–g2, a3–g3, elemental mapping images, and a4–g4, elemental line scan profiles of the intermediate products prepared with different reaction time at 0 min (a), 20 min (b), 1 h (c), 3 h (d), 5 h (e), 7 h (f) and 10 h (g).

1. **Elemental mapping images and elemental line scan profiles of C, O**


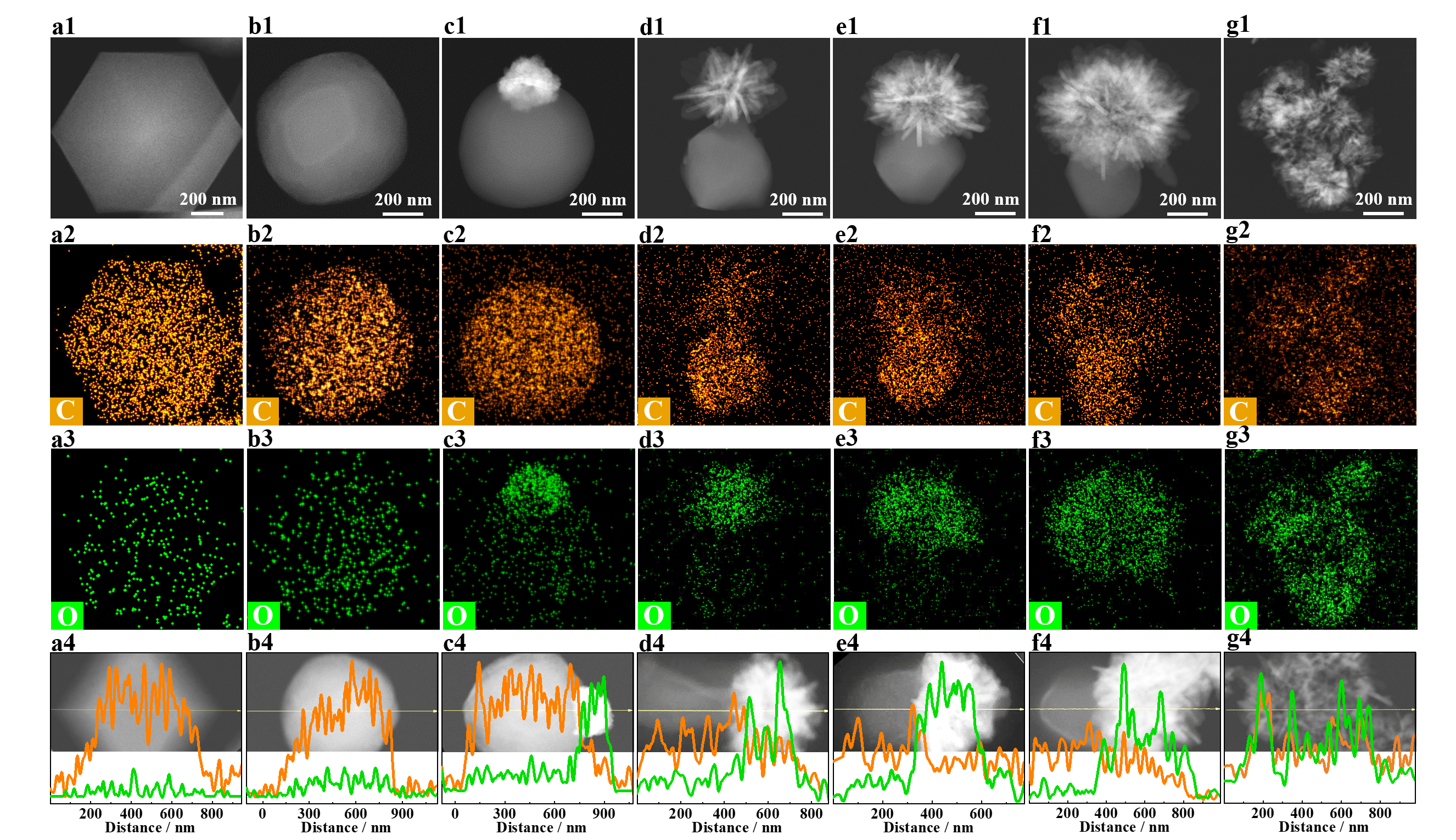


**Figure S12.** Structural transformation of ZIF-8 to ZIF-8/B-MOF to B-MOF. a1–g1, HAADF-STEM, a2–g2, a3–g3, elemental mapping images, and a4–g4, elemental line scan profiles of the intermediate products prepared with different reaction time at 0 min (a), 20 min (b), 1 h (c), 3 h (d), 5 h (e), 7 h (f) and 10 h (g).

1. **FT-IR spectrum**


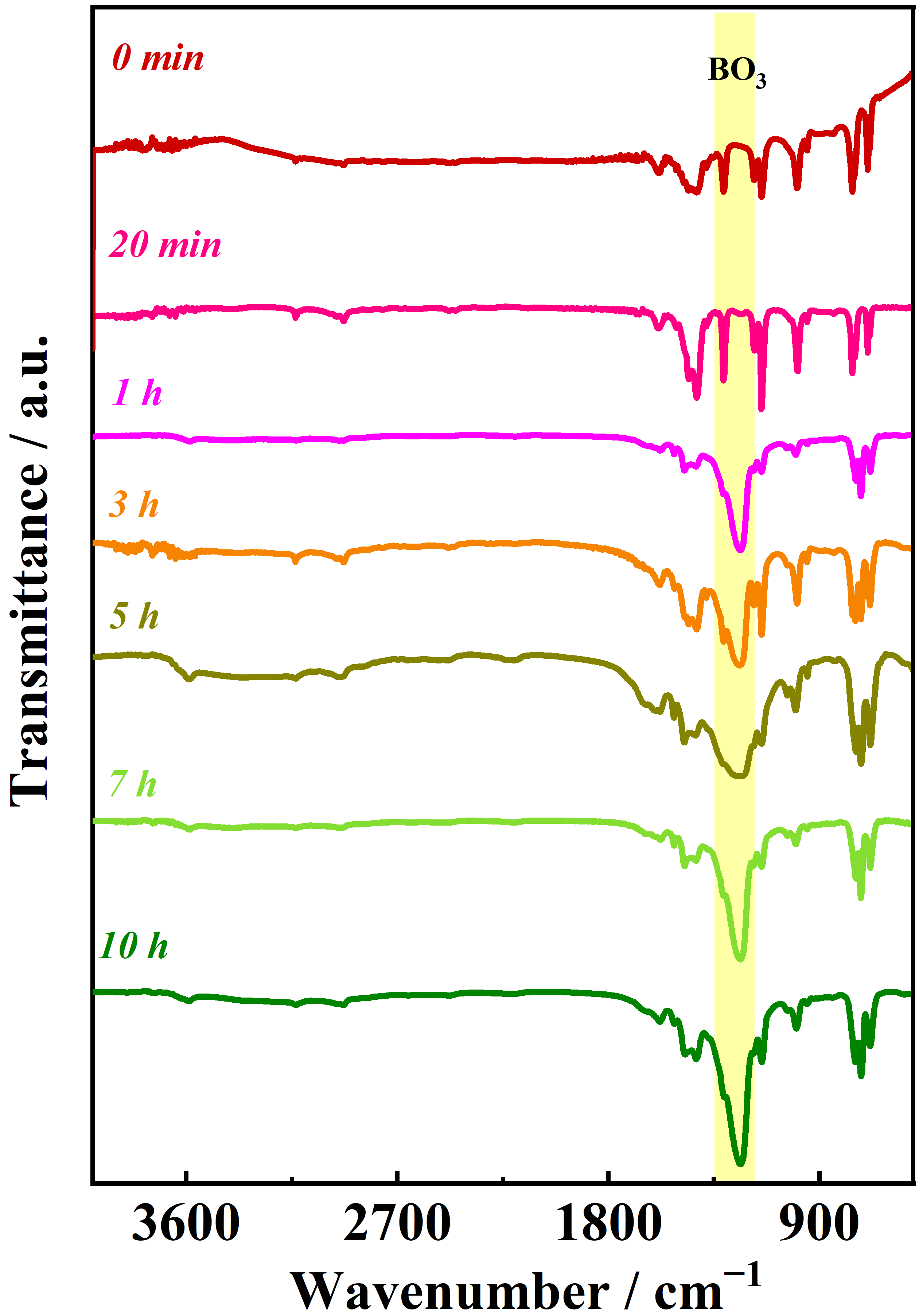


**Figure S13.** FT-IR spectra of intermediate products prepared with different reaction time.

1. **XPS spectra**


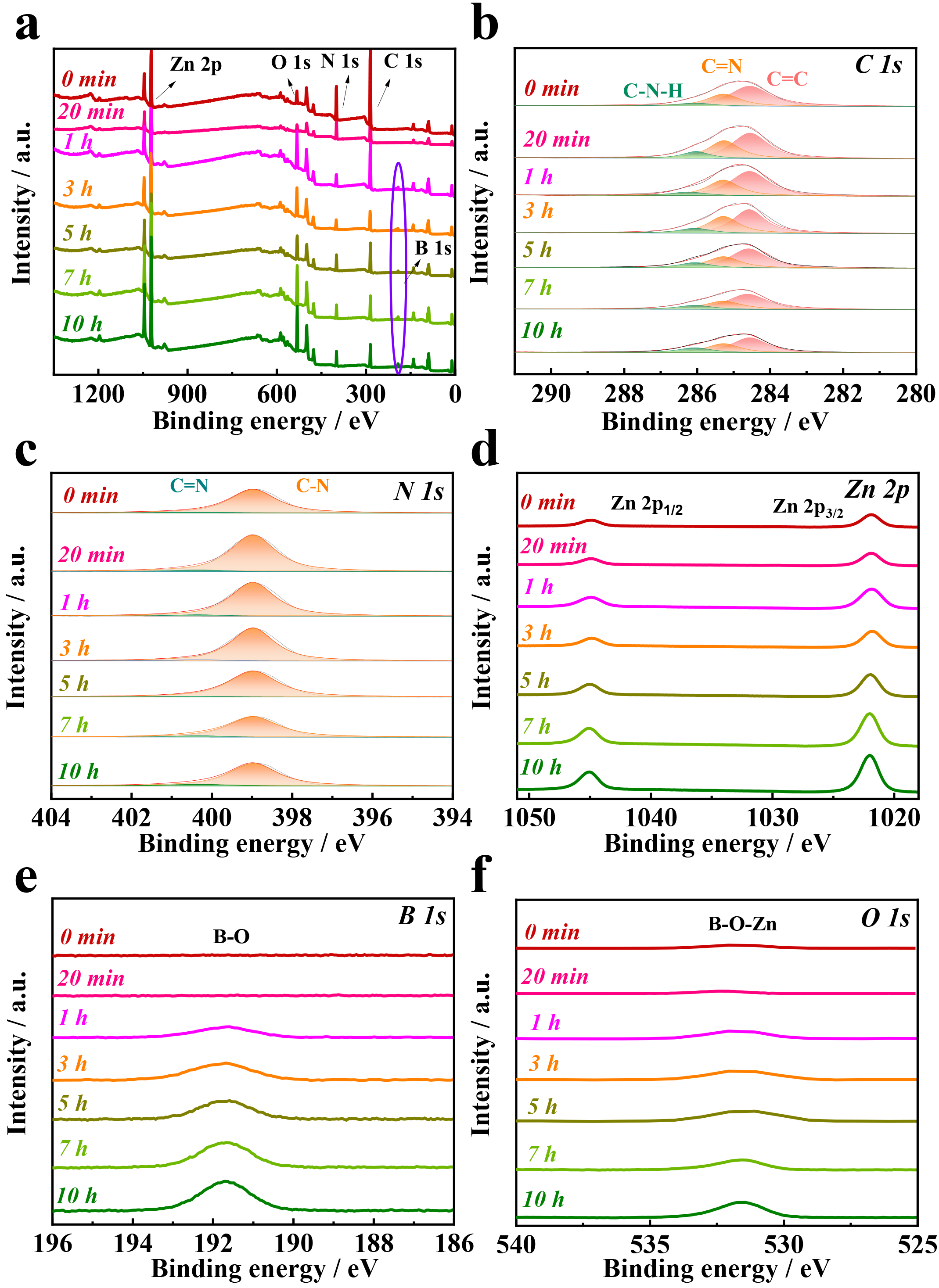


**Figure S14.** XPS characterizations of intermediate products prepared with different reaction time. a–f, Survey XPS spectra (a), high-resolution C 1s spectra (b), high-resolution N 1s spectra (c), high-resolution Zn 2p spectra (d), high-resolution B 1s spectra (e) and high-resolution O 1s spectra (f) of the intermediate products.

**16. Crystal structure**


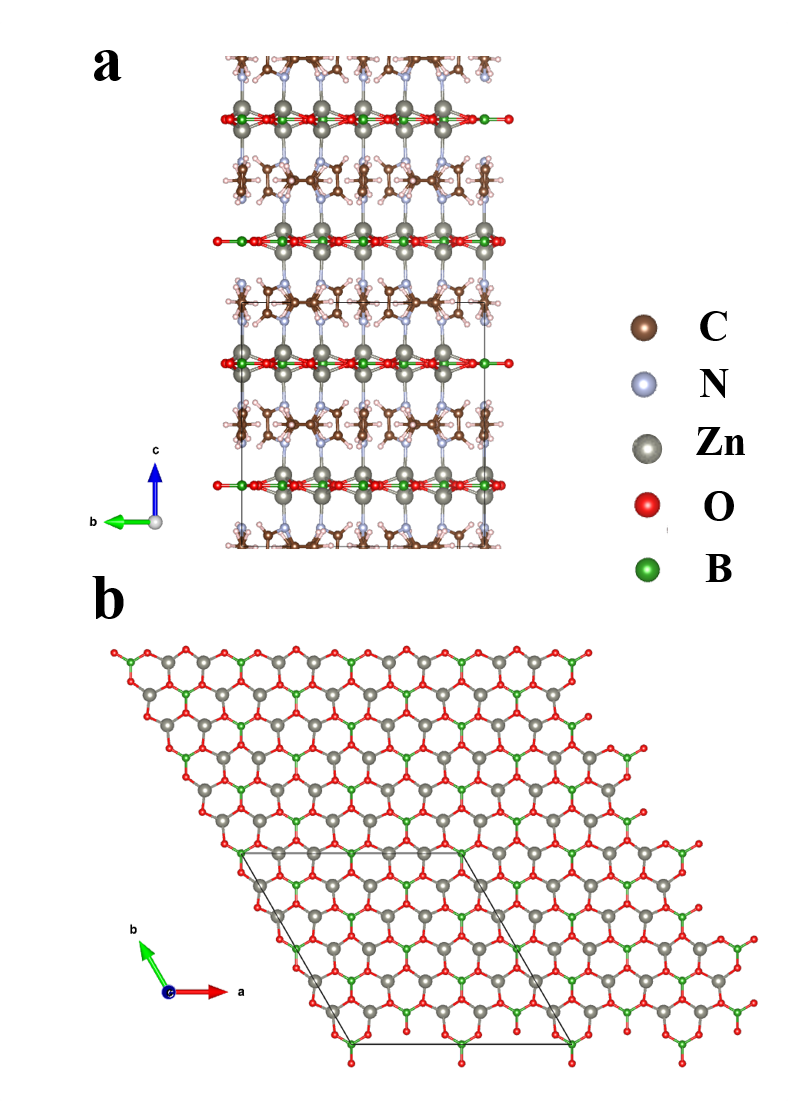


**Figure S15.** Crystal structure of B-MOF. a, Whole crystal structure of B-MOF. b, Crystal structure of the ZnBO layer.

1. **SEM images of ZIF-8 in a methanol solution of H_3_BO_3_ at different temperatures**


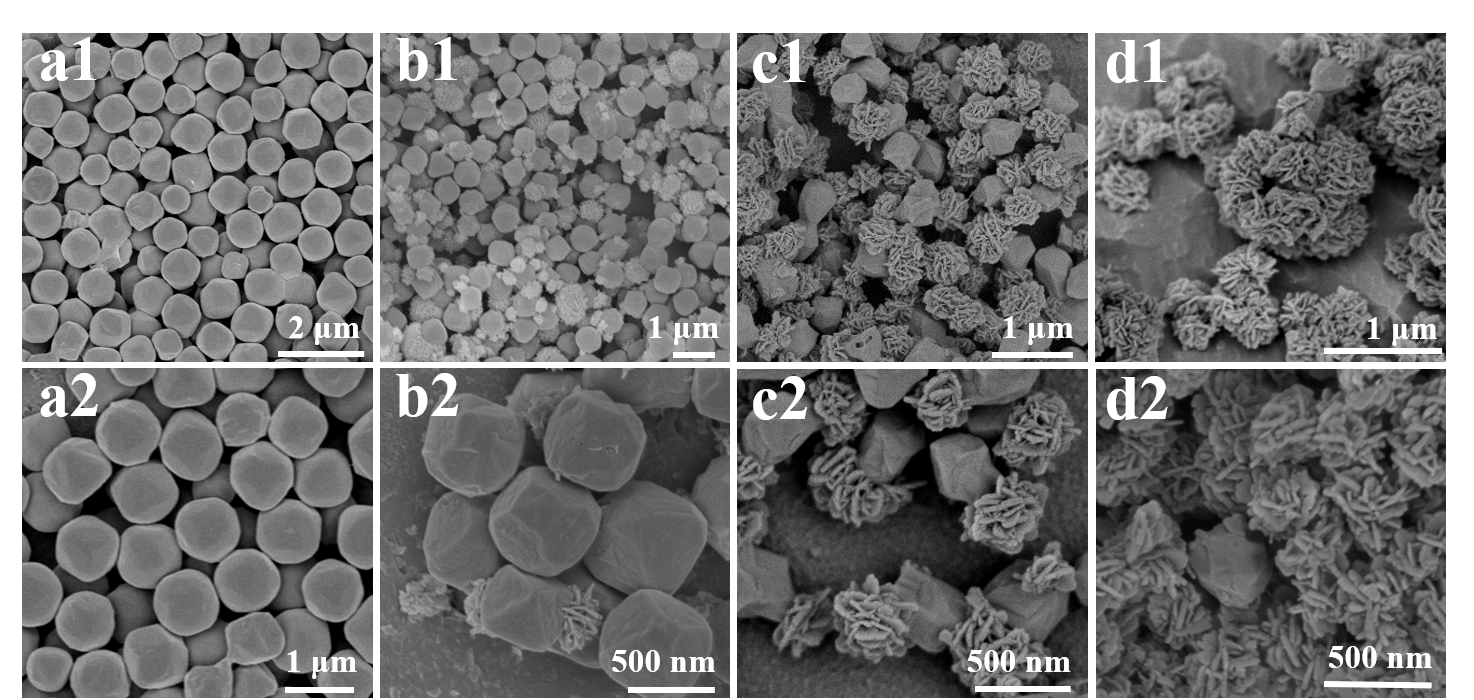


**Figure S16.** Structural transformation of ZIF-8 in a methanol solution of H_3_BO_3_ at different temperatures. a–d, SEM images of the controlled samples prepared at 110 ^o^C (a), 130 ^o^C (b), 150 ^o^C (c) and 170 ^o^C (d).

1. **SEM images of ZIF-8 in different solutions of H_3_BO_3_**

**
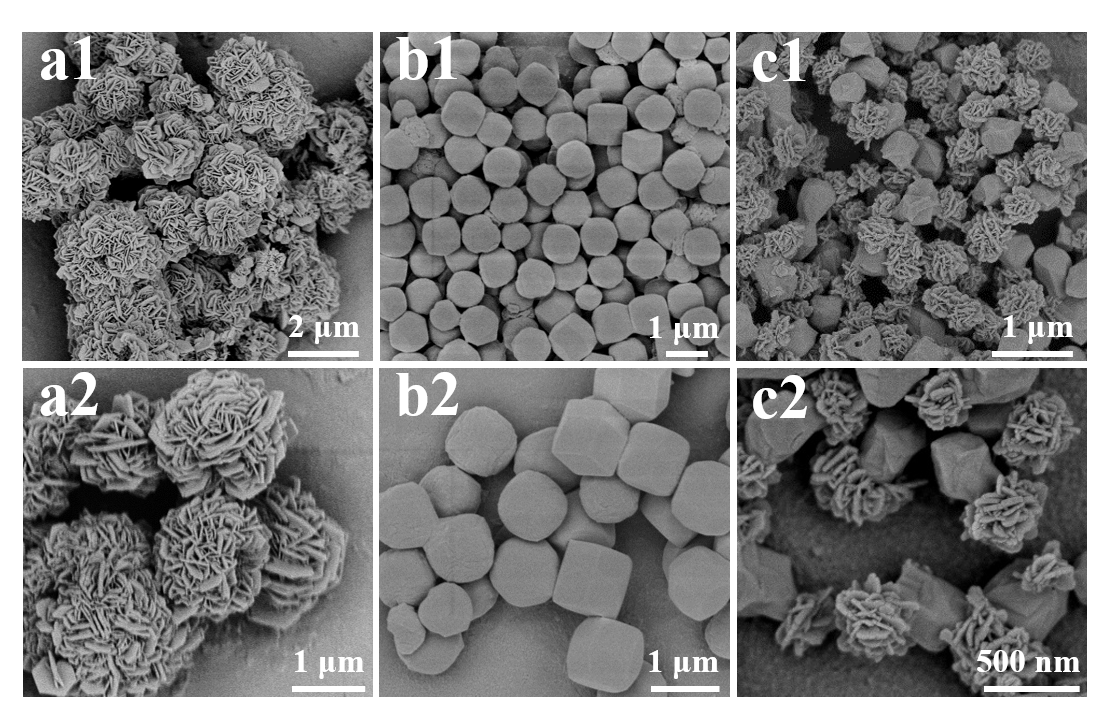
**

**Figure S17.** Structural transformation of ZIF-8 in different solutions of H_3_BO_3_. a–c, SEM images of the controlled samples prepared at 150 ^o^C with different solvents: deionized water (a), ethanol (b) and methanol (c).

1. **SEM images of ZIF-8 in methanol solution with different concentrations of H_3_BO_3_**


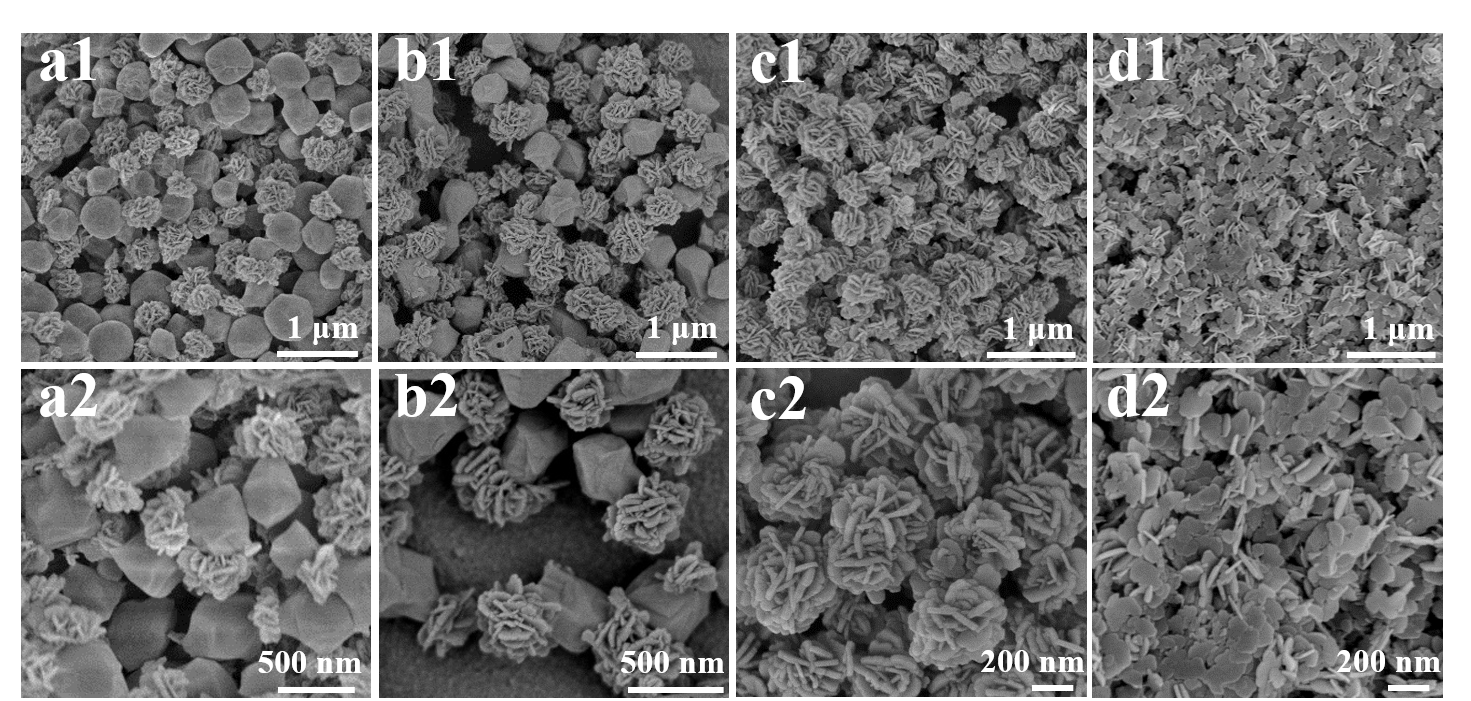


**Figure S18.** Structural transformation of ZIF-8 in methanol solution with different concentrations of H_3_BO_3_. a–d, SEM images of the controlled samples prepared at 150 ^o^C in 10 mM H_3_BO_3_ (a), 20 mM H_3_BO_3_ (b), 30 mM H_3_BO_3_ (c) and 40 mM H_3_BO_3_ (d).

1. **Characterizations and microstructure of the control sample synthesized in one pot**


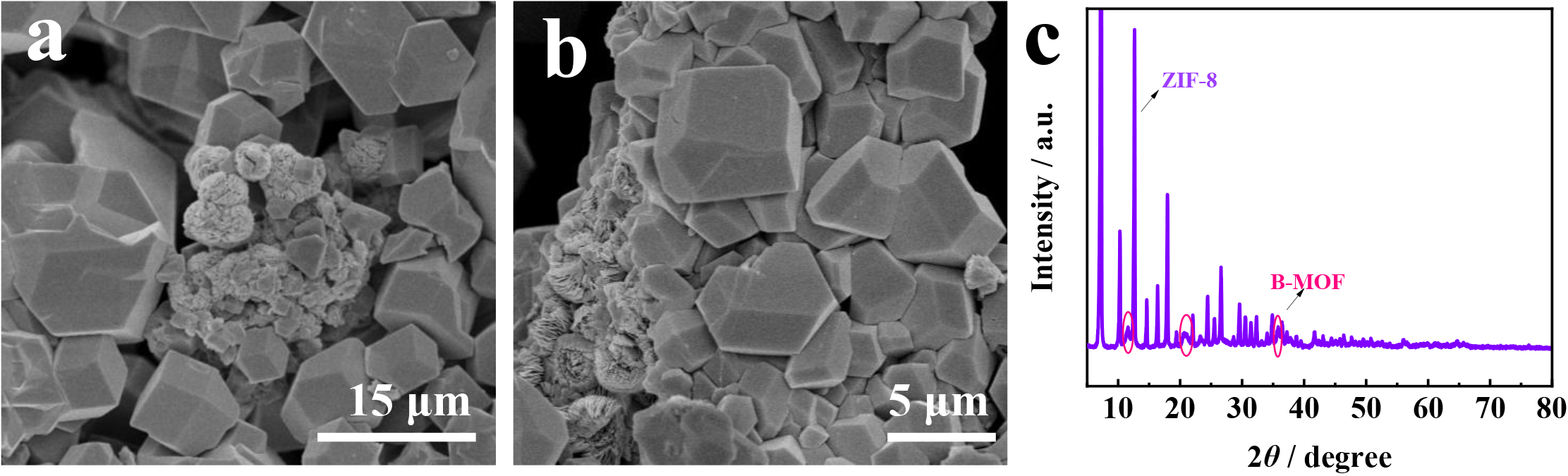


**Figure S19.** Characterizations and microstructure of the control sample synthesized in one pot. a–c, SEM images (a and b) and XRD patterns (c) of the control sample synthesized by heating Zn(AC)_2_∙2H_2_O, 2-MeIm and H_3_BO_3_ in methanol at 150 ℃ for 12 h.

1. **SEM images of different bimetallic ZIFs**


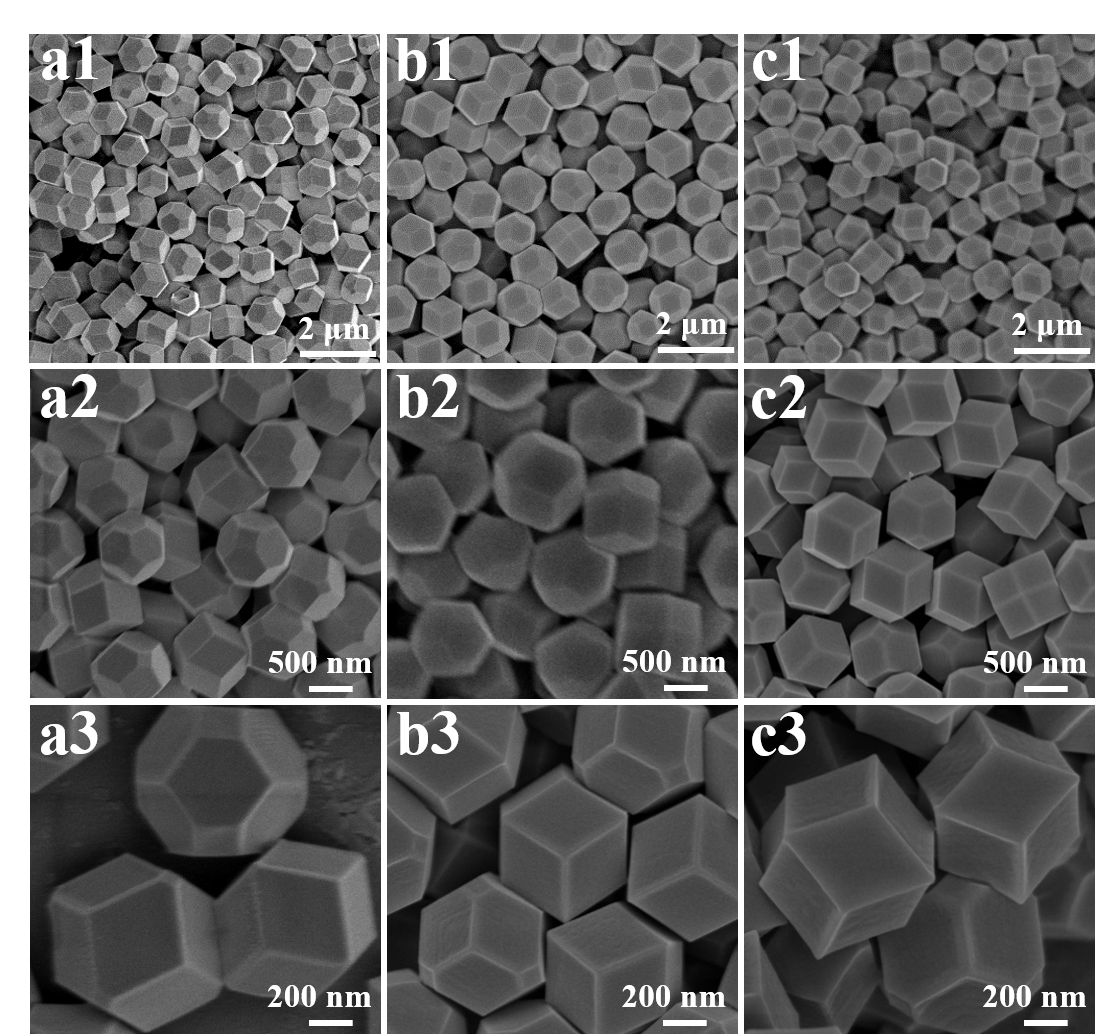


**Figure S20.** Microstructures of different bimetallic ZIFs. a–c, SEM images of ZnCo-ZIF (a), ZnNi-ZIF (b) and ZnCu-ZIF (c).

1. **SEM images** **of different bimetallic ZnM-ZIF/B-MOF heterostructures (M = Co, Ni, Cu)**


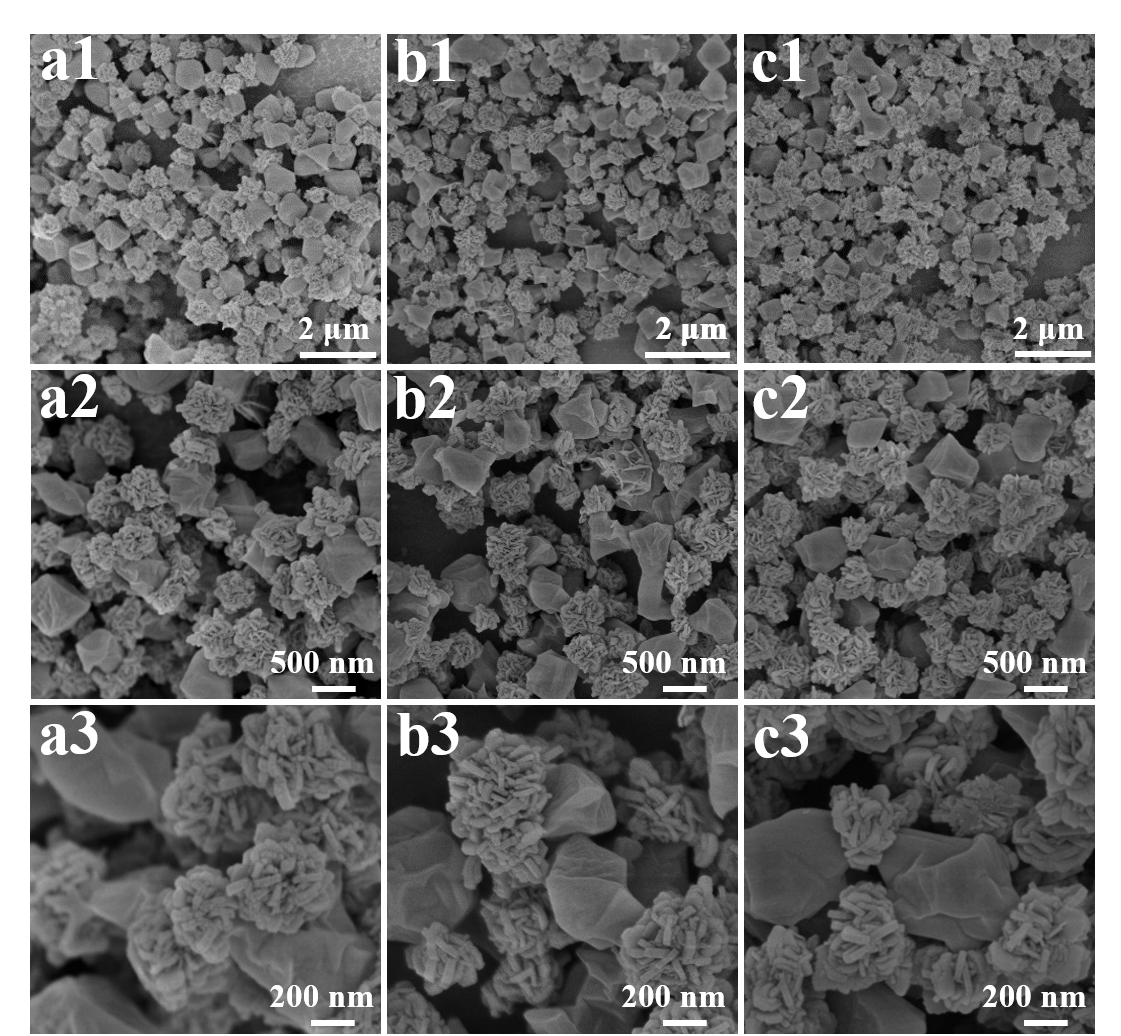


**Figure S21.** Microstructures of different bimetallic ZnM-ZIF/B-MOF heterostructures (M = Co, Ni, Cu). a–c, SEM images of ZnCo-ZIF/B-MOF (a), ZnNi-ZIF/B-MOF (b) and ZnCu-ZIF/B-MOF (c).

1. **XRD characterizations of ZnM-ZIF/B-MOF heterostructures (M = Co, Ni, Cu)**


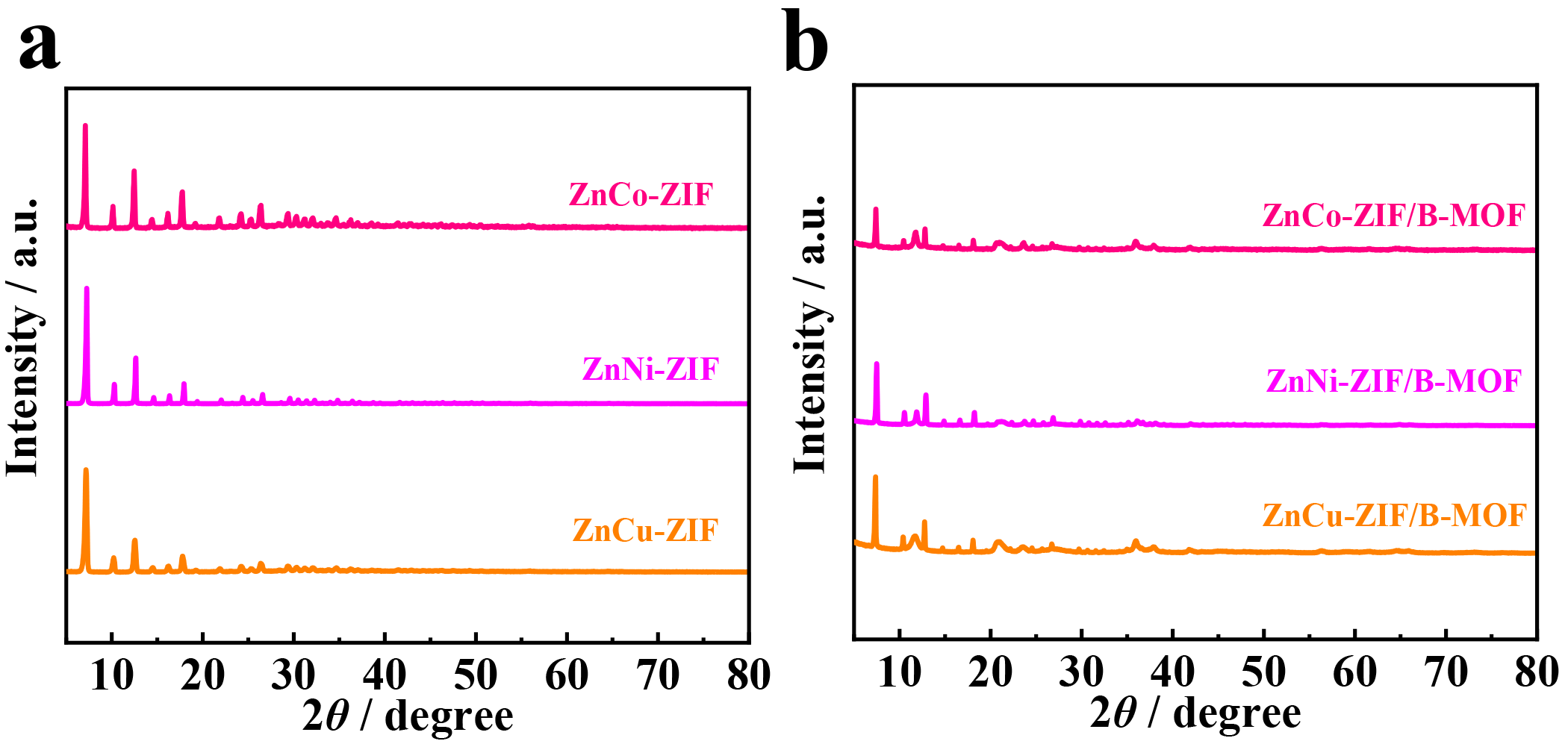


**Figure S22.** XRD characterizations. a, XRD patterns of ZnCo-ZIF, ZnNi-ZIF and ZnCu-ZIF. b, XRD patterns of ZnCo-ZIF/B-MOF, ZnNi-ZIF/B-MOF and ZnCu-ZIF/B-MOF.

1. **XPS characterizations of ZnM-ZIF/B-MOF heterostructures (M = Co, Ni, Cu)**


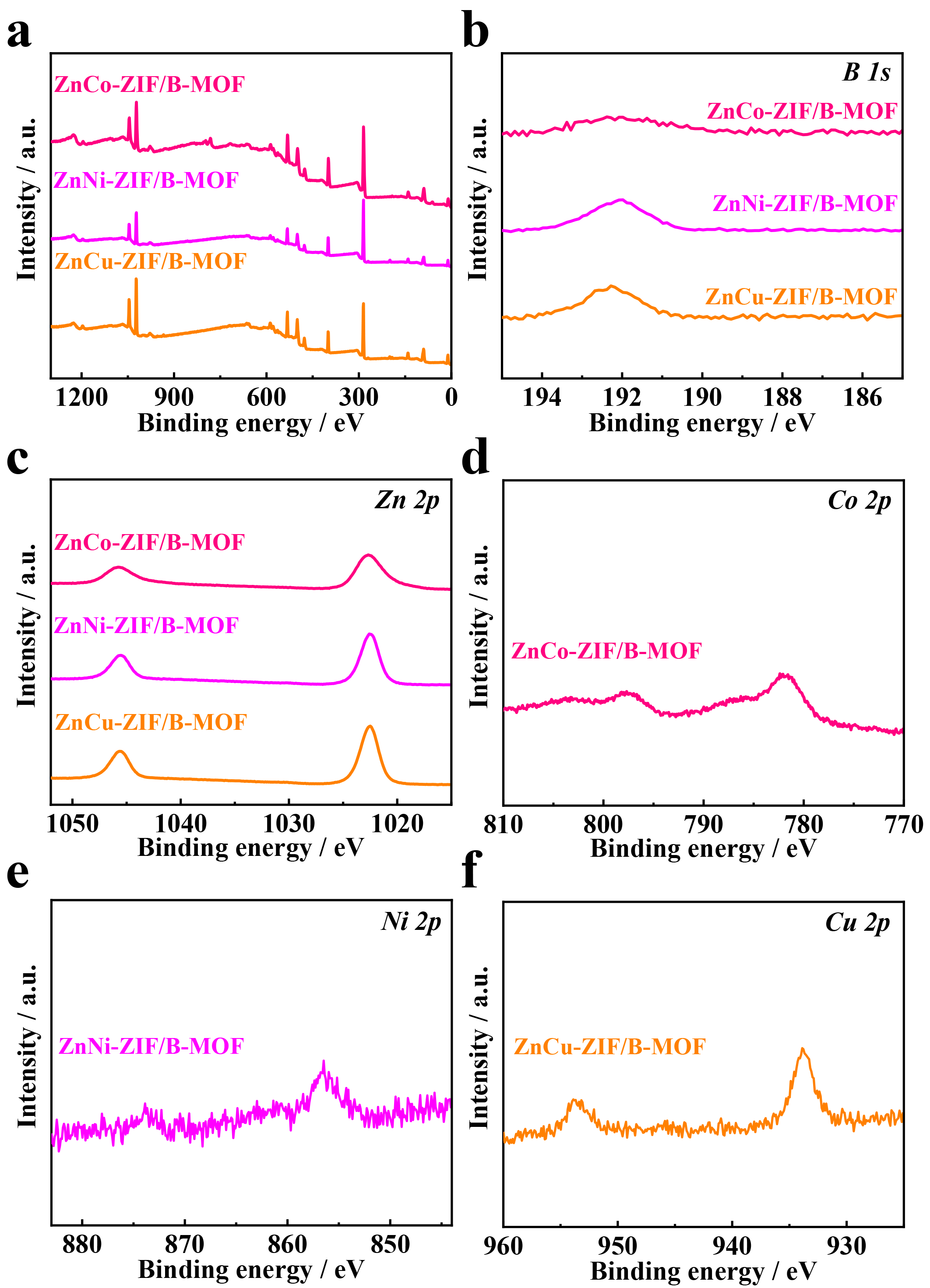


**Figure S23.** XPS characterizations of ZnM-ZIF/B-MOF heterostructures (M = Co, Ni, Cu). a–f, Survey XPS spectra (a), high-resolution B 1s spectra (b), high-resolution Zn 2p spectra (c), high-resolution Co 2p spectra (d), high-resolution Ni 2p spectra (e) and high-resolution Cu 2p spectra (f).

1. **HAADF-STEM and elemental mapping images** **of ZnM-ZIF/B-MOF heterostructures (M = Co, Ni, Cu)**


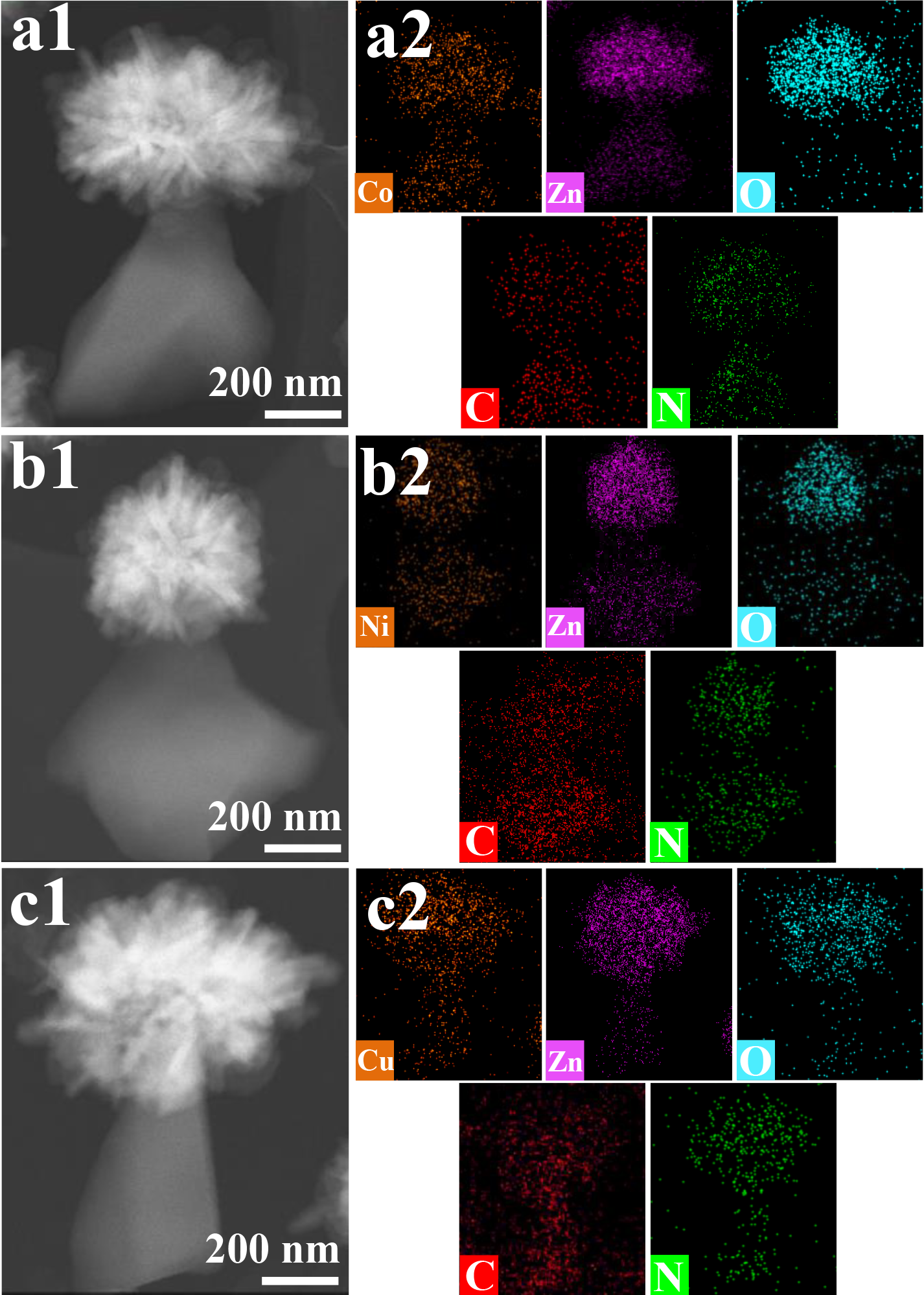


**Figure S24.** Microstructure of ZnM-ZIF/B-MOF heterostructures (M = Co, Ni, Cu). a1–c1, HAADF-STEM, a2–c2, elemental mapping images of ZnCo-ZIF/B-MOF (a), ZnNi-ZIF/B-MOF (b) and ZnCu-ZIF/B-MOF (c).

1. **SEM images of NC/BNC**


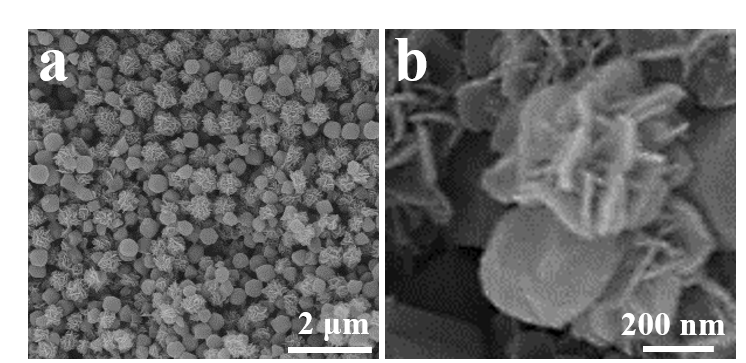


**Figure S25.** Microstructure of NC/BNC. a, b, SEM images of NC/BNC.

1. **Microstructure of NC/BNC**


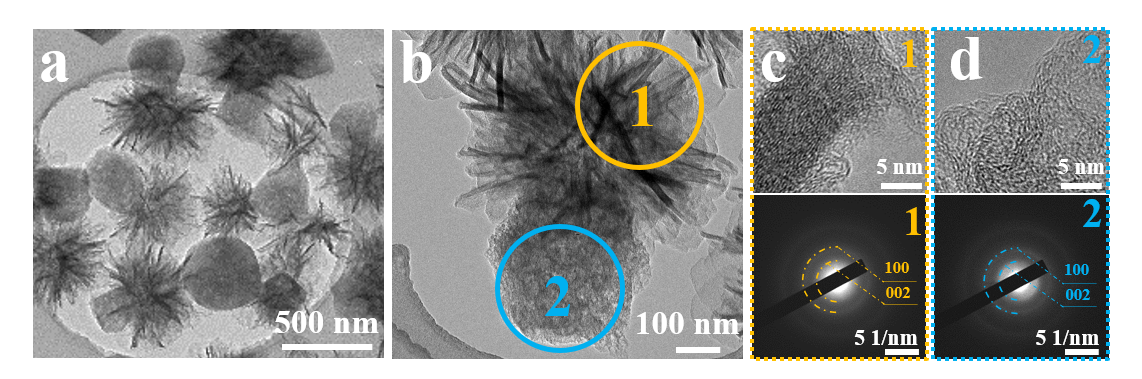


**Figure S26.** Microstructure of NC/BNC. a, b, TEM images of NC/BNC. c, d, HRTEM and SAED patterns in different regions, represented by yellow circles (region 1) and blue circles (region 2), respectively.

1. **EPR spectra of ZIF-8, ZIF-8/B-MOF, and B-MOF**


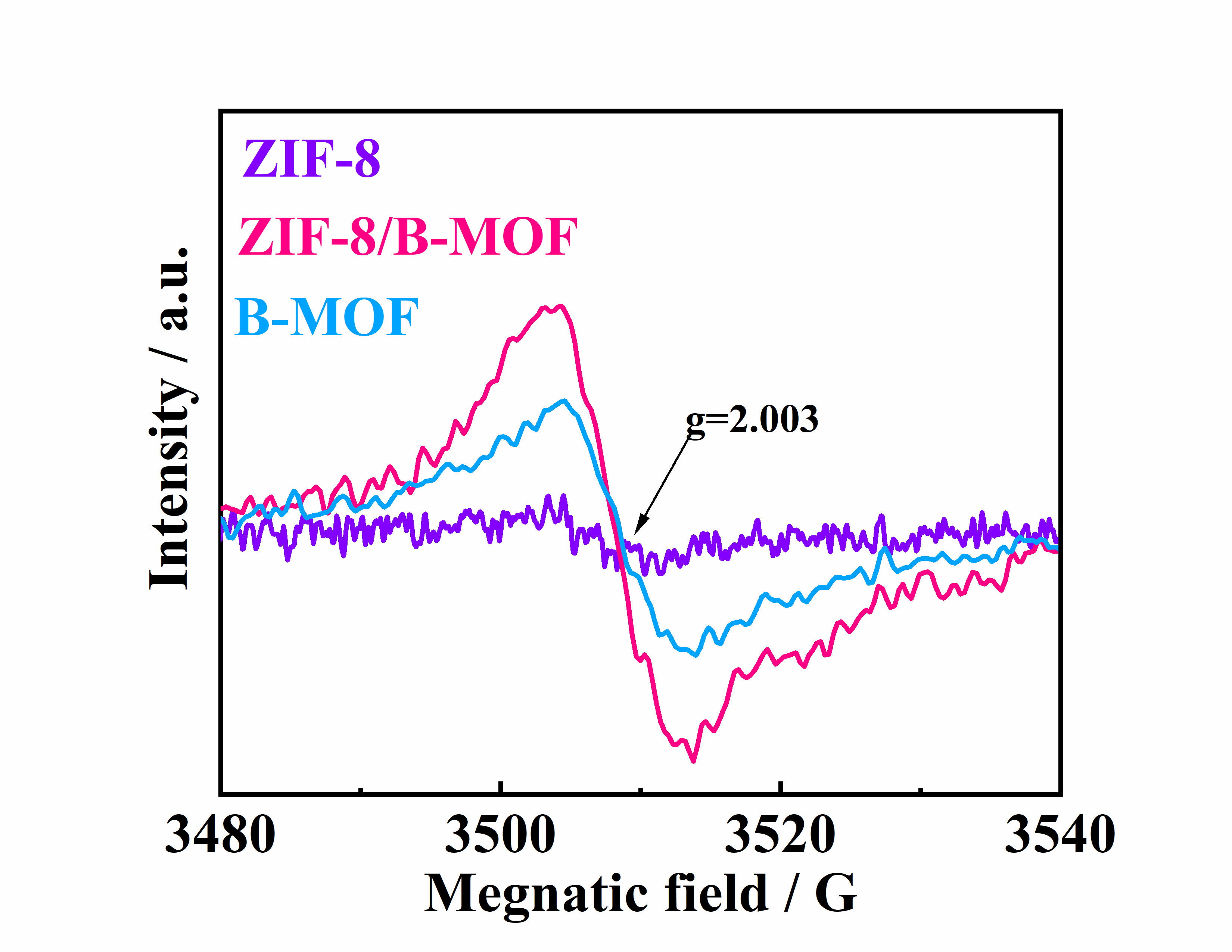


**Figure S27.** Room temperature EPR spectra of ZIF-8, ZIF-8/B-MOF, and B-MOF.

1. **HAADF-STEM and elemental mapping images of NC/BNC**

**
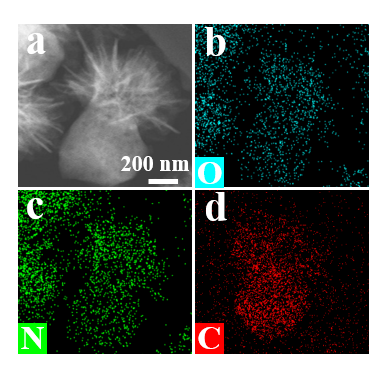
**

**Figure S28.** Microstructure of NC/BNC. a, HAADF-STEM, b–d, elemental mapping images of NC/BNC.

1. **XPS and EELS characterizations of NC derived from ZIF-8-110**


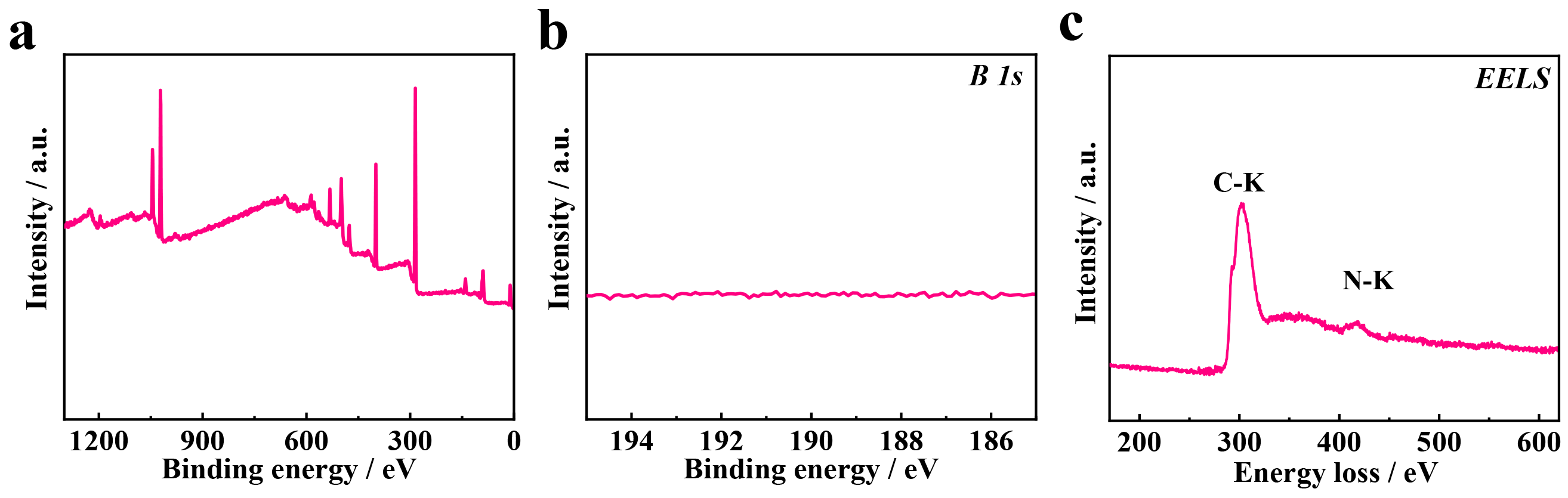


**Figure S29.** a, Survey XPS spectra, b, high-resolution B 1s spectra and EELS spectra of NC derived from ZIF-8 treated in a methanol solution of H_3_BO_3_ at 110 ℃.

1. **Pore size distribution of NC, NC/BNC, and BNC**


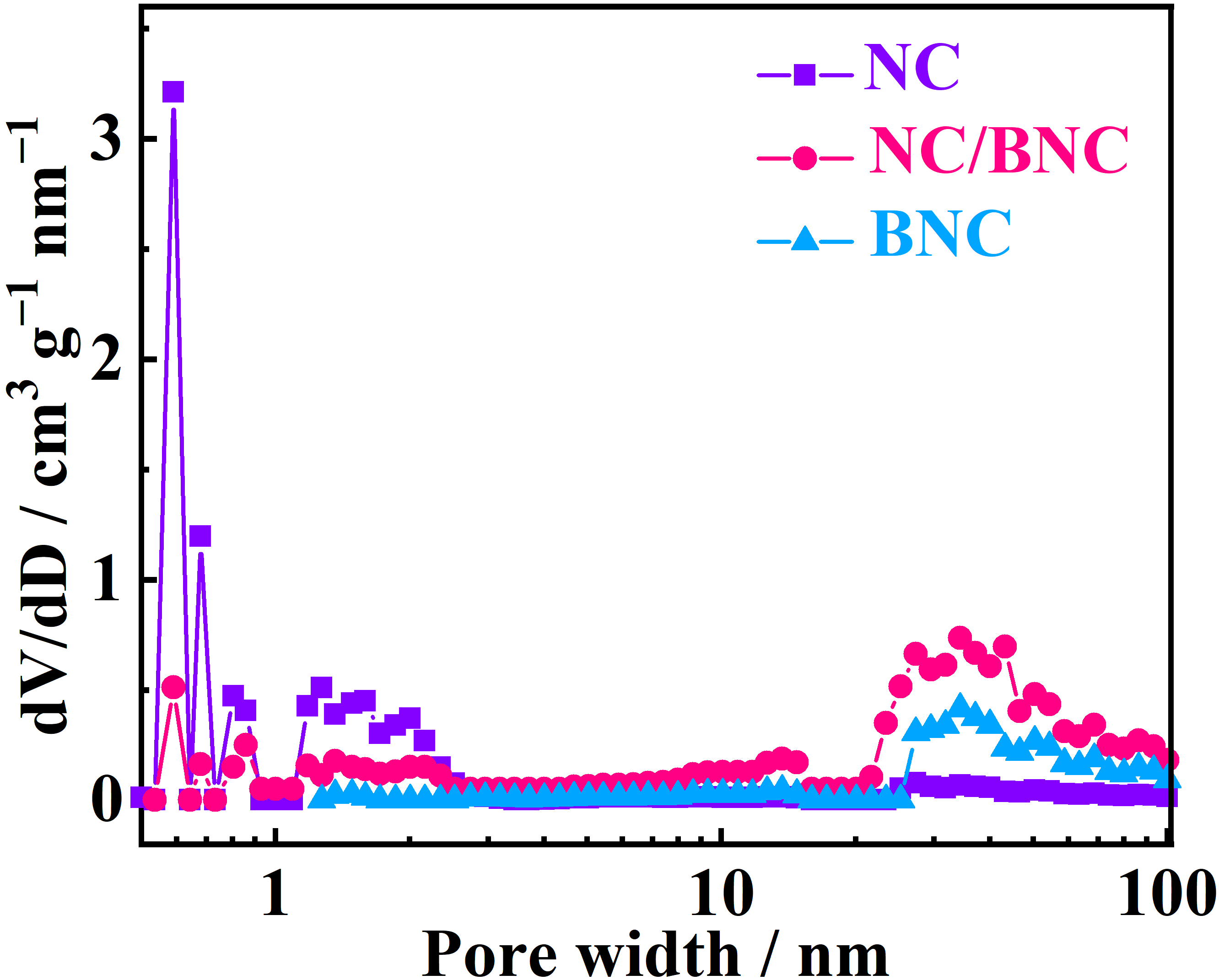


**Figure S30.** The corresponding pore size distribution of NC, NC/BNC, and BNC.

1. **Survey XPS spectra of NC, NC/BNC and BNC**


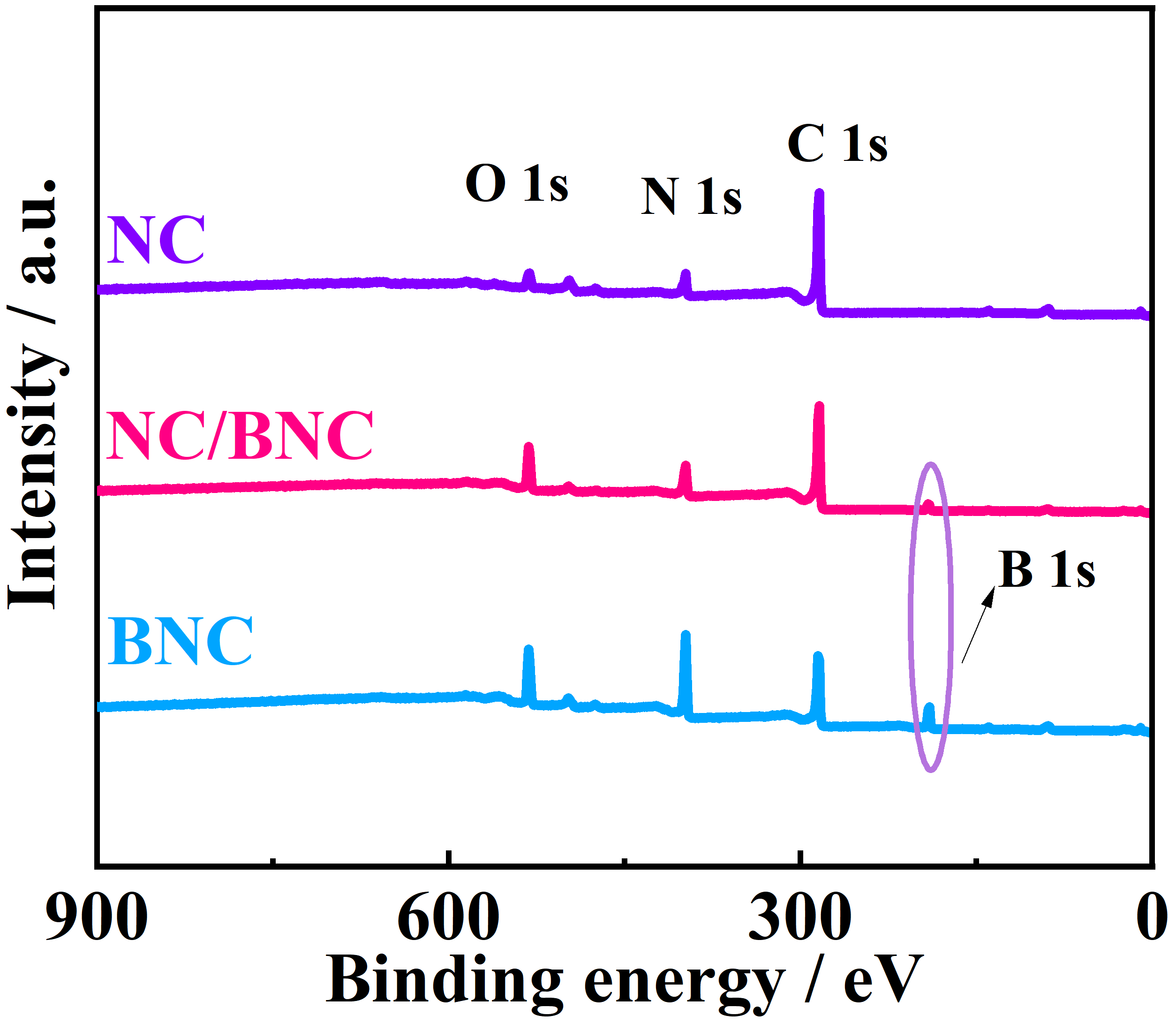


**Figure S31.** Survey XPS spectra of NC, NC/BNC and BNC.

1. **Electrocatalytic N_2_H_4_ performance**


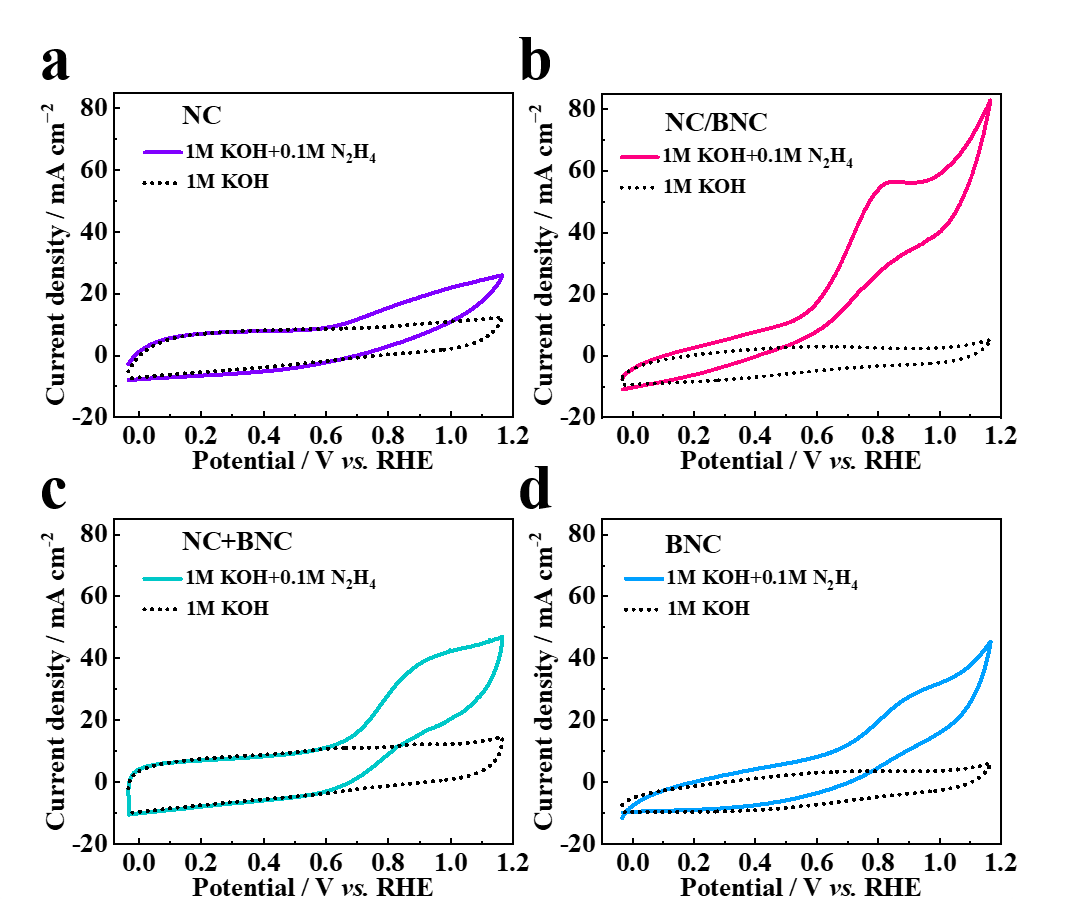


**Figure S32.** Electrocatalytic N_2_H_4_ performance. a–d, CV curves of NC (a), NC/BNC (b), NC+BNC (c) and BNC (d) electrodes in the 1 M KOH without (dashed lines) or with (solid lines) 0.1 M N_2_H_4_ solution at a scan rate of 50 mV s^−1^.

1. **CV curves of NC/BNC and carbon cloth electrodes**


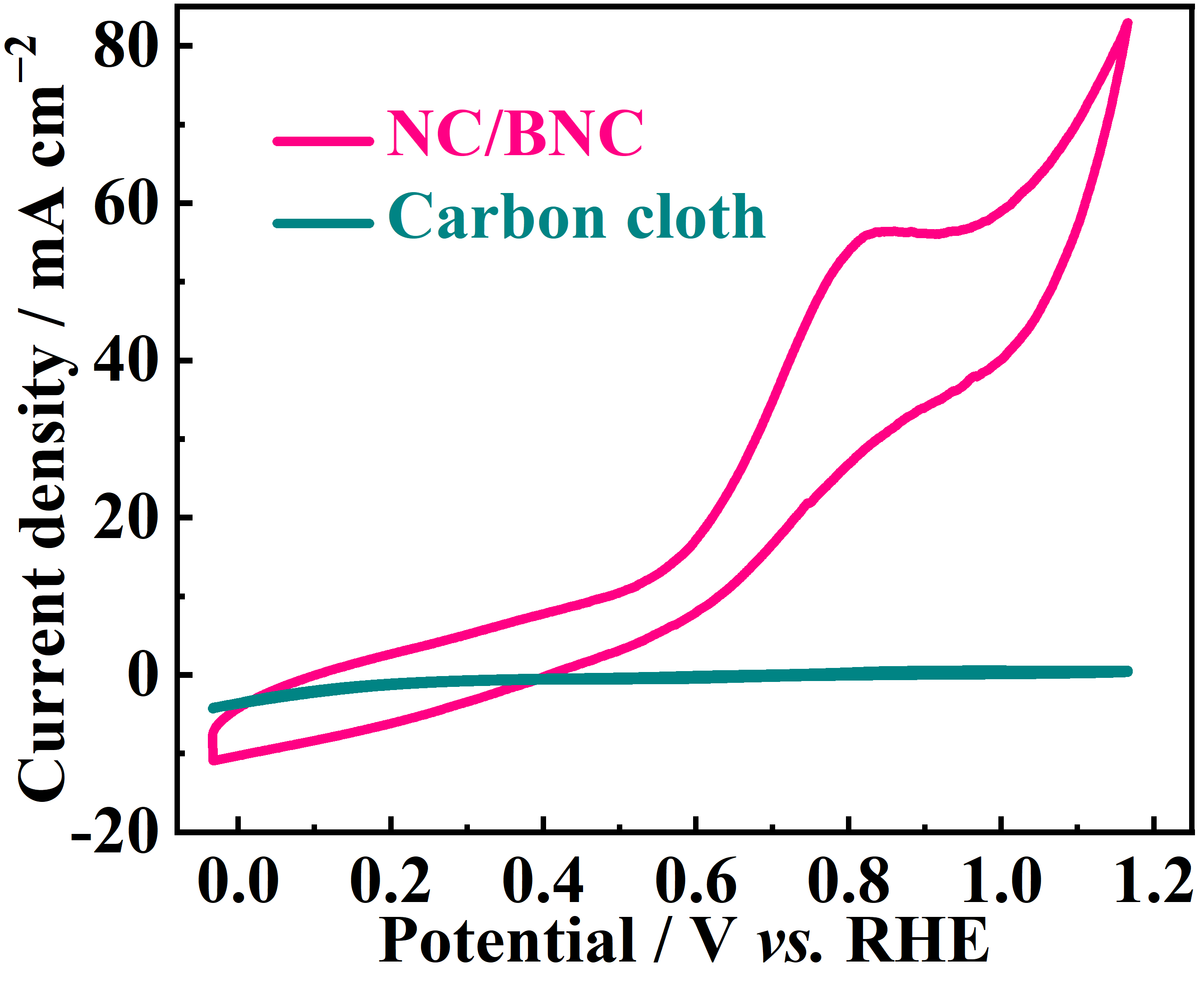


**Figure S33.** CV curves of NC/BNC and carbon cloth electrodes in the 1 M KOH with 0.1 M N_2_H_4_ solution at a scan rate of 50 mV s^−1^.

1. **Photograph of NC/BNC**

**
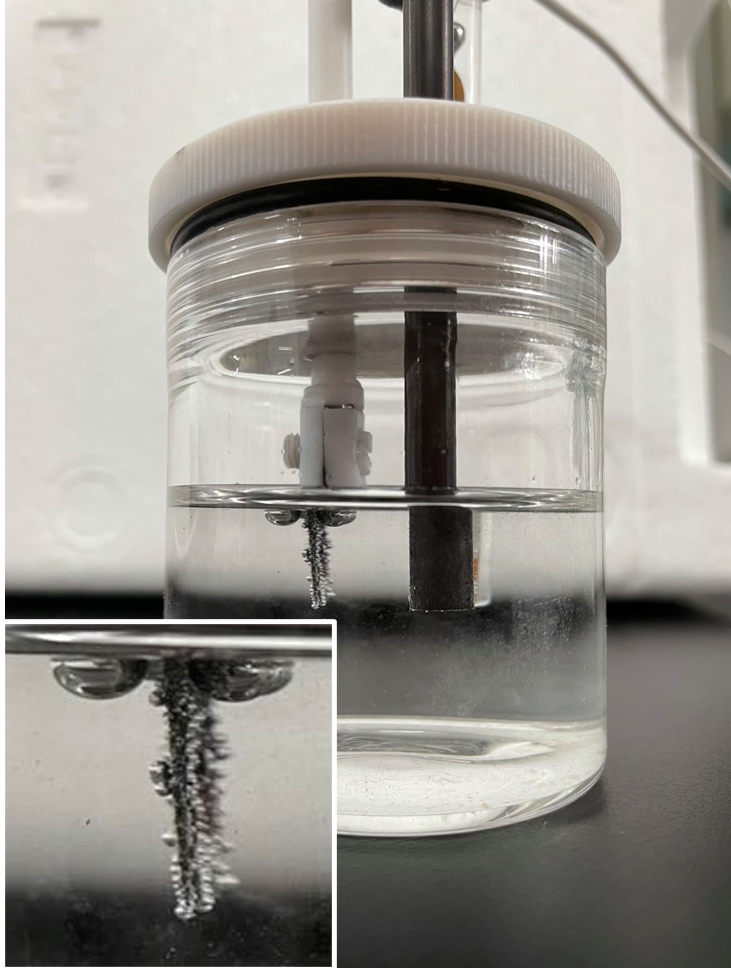
**

**Figure S34.** Photograph of vigorous bubbling on NC/BNC during anodic scans.

1. **Microstructure of NC**


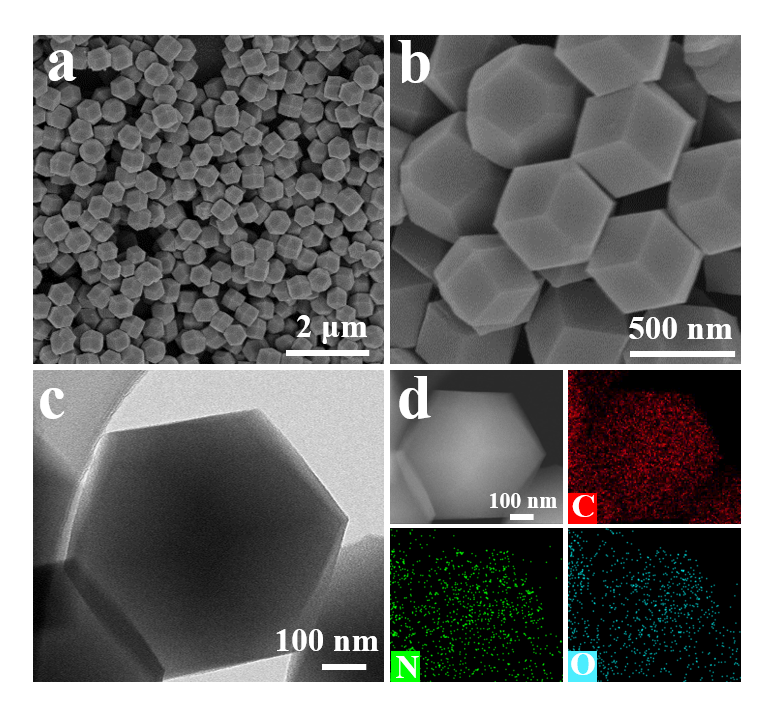


**Figure S35.** Microstructure of NC. a, b, SEM images of NC. c, HAADF-STEM and d, corresponding elemental mapping images of NC.

1. **Microstructure of BNC**


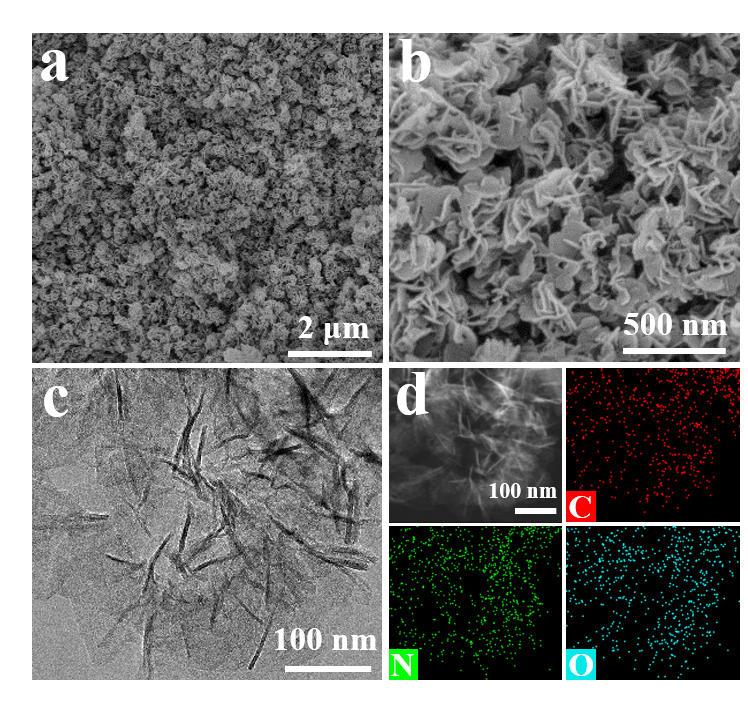


**Figure S36.** Microstructure of BNC. a, b, SEM images of BNC. c, HAADF-STEM and d, corresponding elemental mapping images of BNC.

1. **Electrochemical double-layer capacitance measurement**


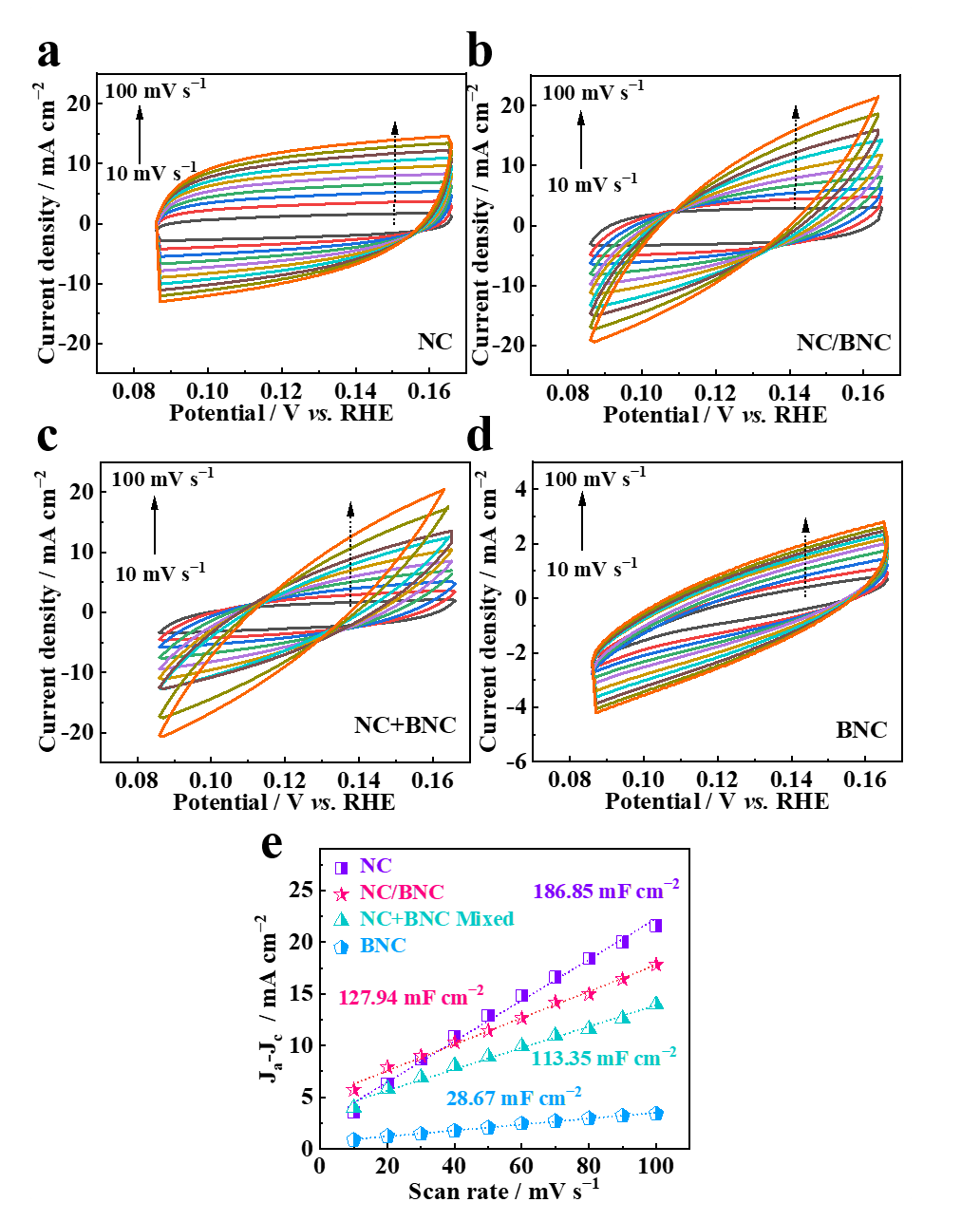


**Figure S37.** Electrochemical double-layer capacitance measurement. a–d, CV curves of NC (a), NC/BNC (b), NC+BNC (c) and BNC (d) at different scanning rates. e, ΔJ fitting for samples (ΔJ = Ja − Jc) vs scan rates at a set potential of +0.126 V vs. RHE.

1. **LSV polarization curves**


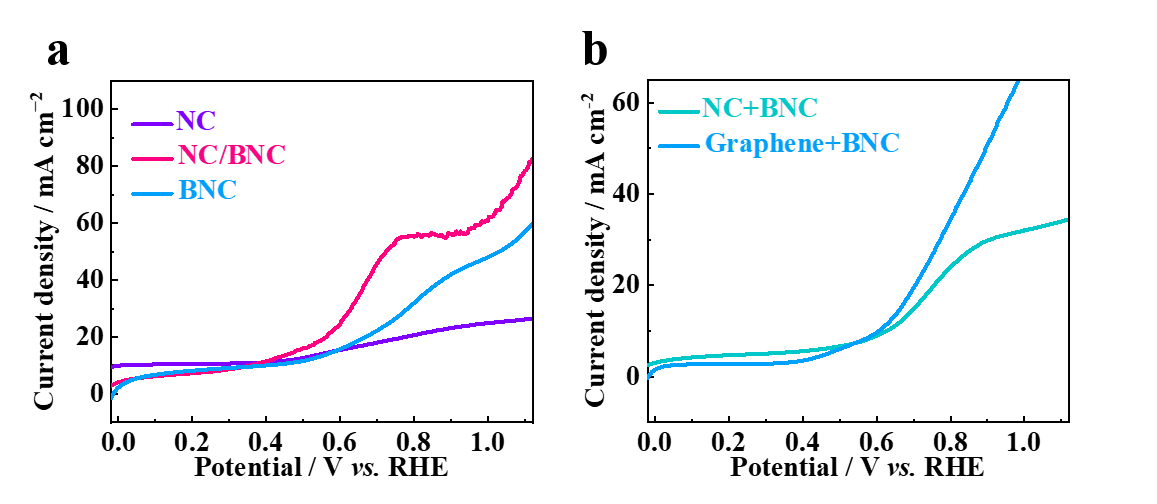


**Figure S38.** a, LSV polarization curves of samples after normalized by the accessible catalytic active sites of electrocatalysts. b, LSV curves of a physical mixture of NC and BNC (NC+BNC) and a physical mixture of graphene and BNC (graphene+BNC) in 1 M KOH with 0.1 M N_2_H_4_ solution at a scan rate of 5 mV s^−1^.

1. **Atomic structure models**


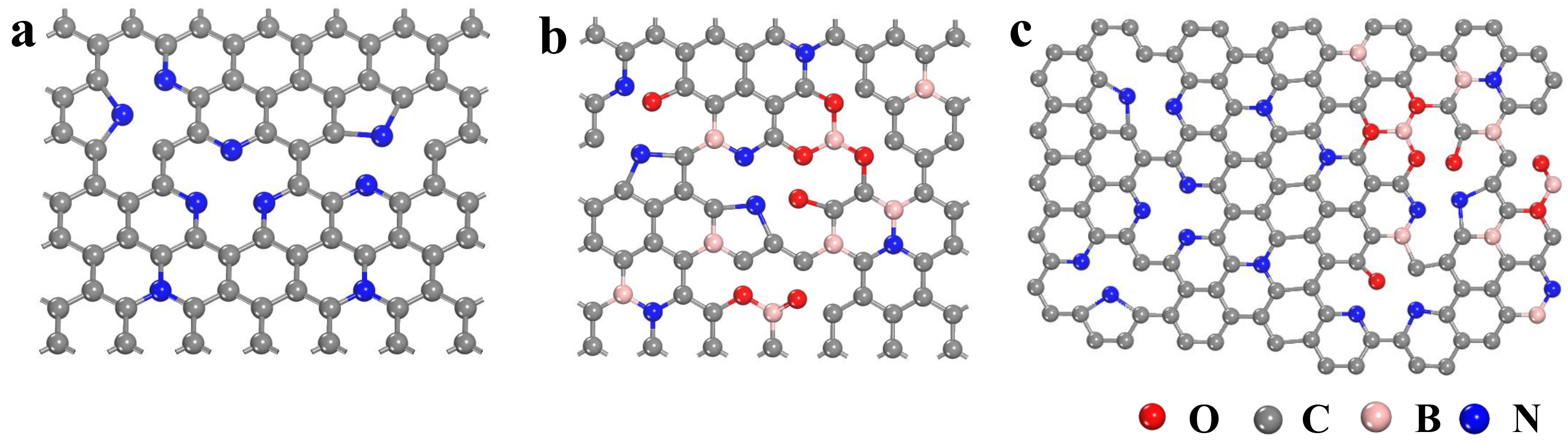


**Figure S39.** Atomic structure models. a–c, Atomic structure models of NC (a), BNC (b) and NC/BNC (c).

1. **Bader charges**


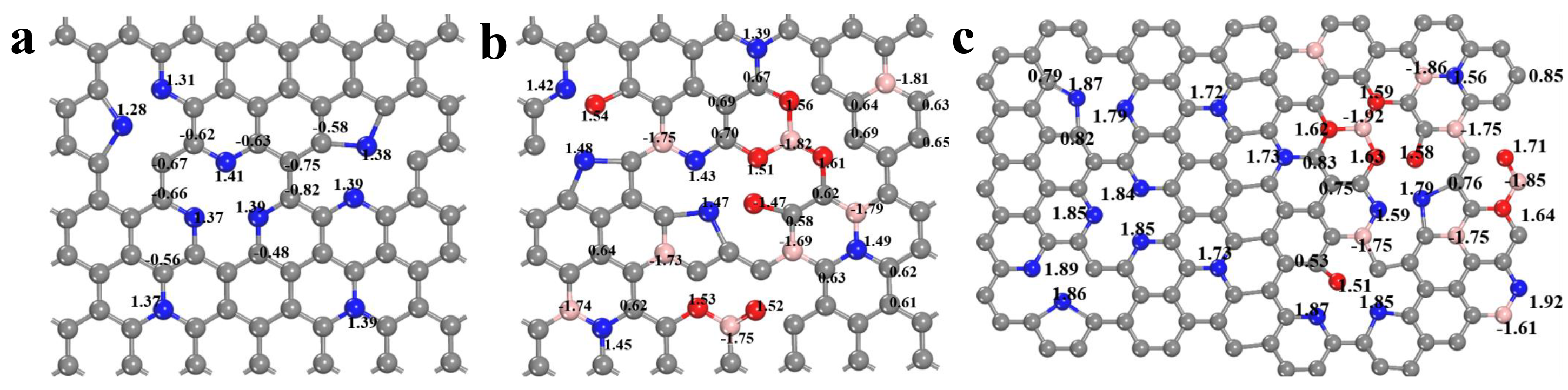


**Figure S40.** Bader charges. a–c, The Bader charges carried by C, N, B and O atoms labeled with numbers of NC (a), BNC (b) and NC/BNC (c).

1. **The adsorption energies of N_2_H_4_ on different adsorption sites of NC**


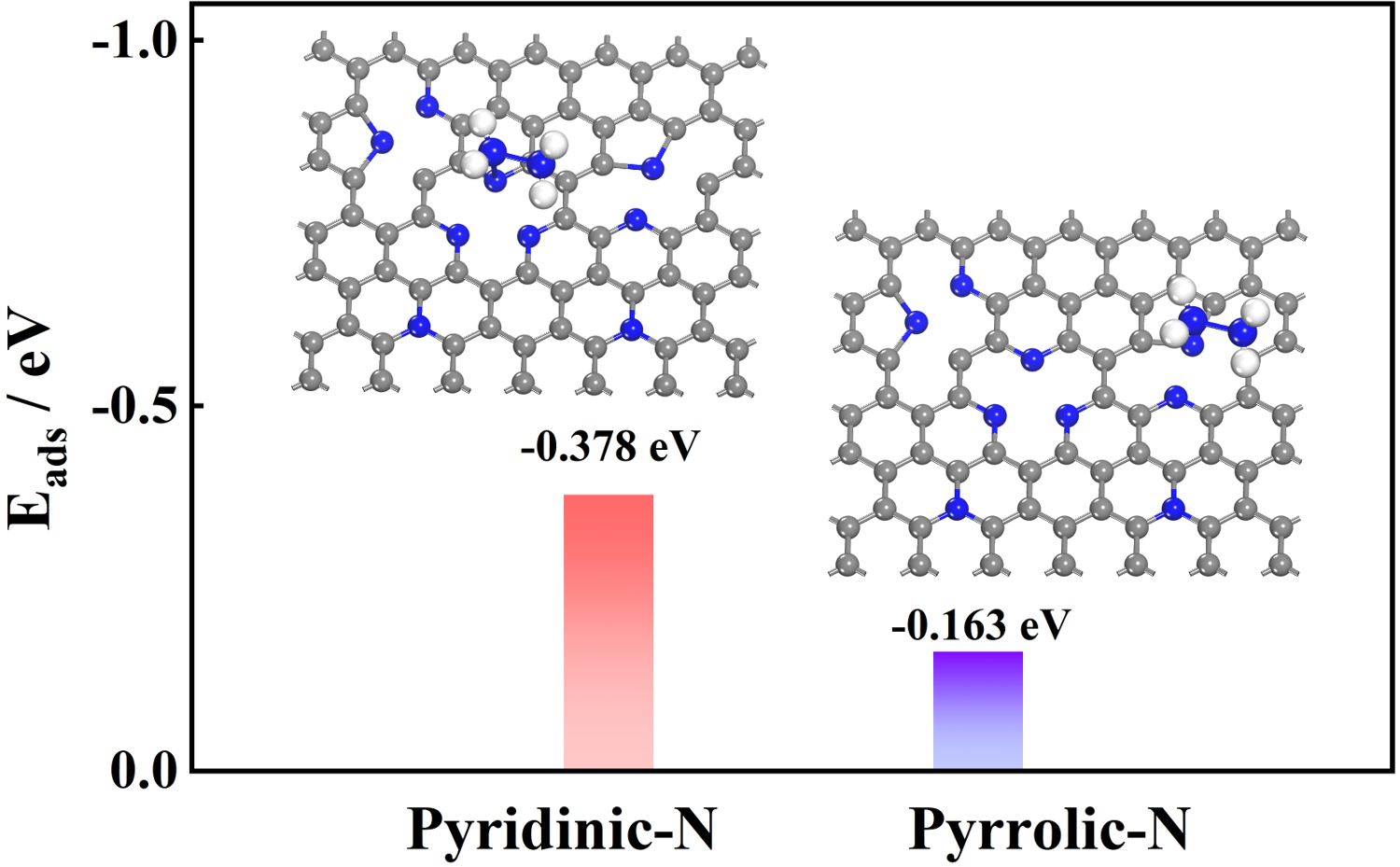


**Figure S41.** The adsorption energies of N_2_H_4_ on different adsorption sites of NC.

1. **The adsorption energies of N_2_H_4_ on different adsorption sites of BNC**


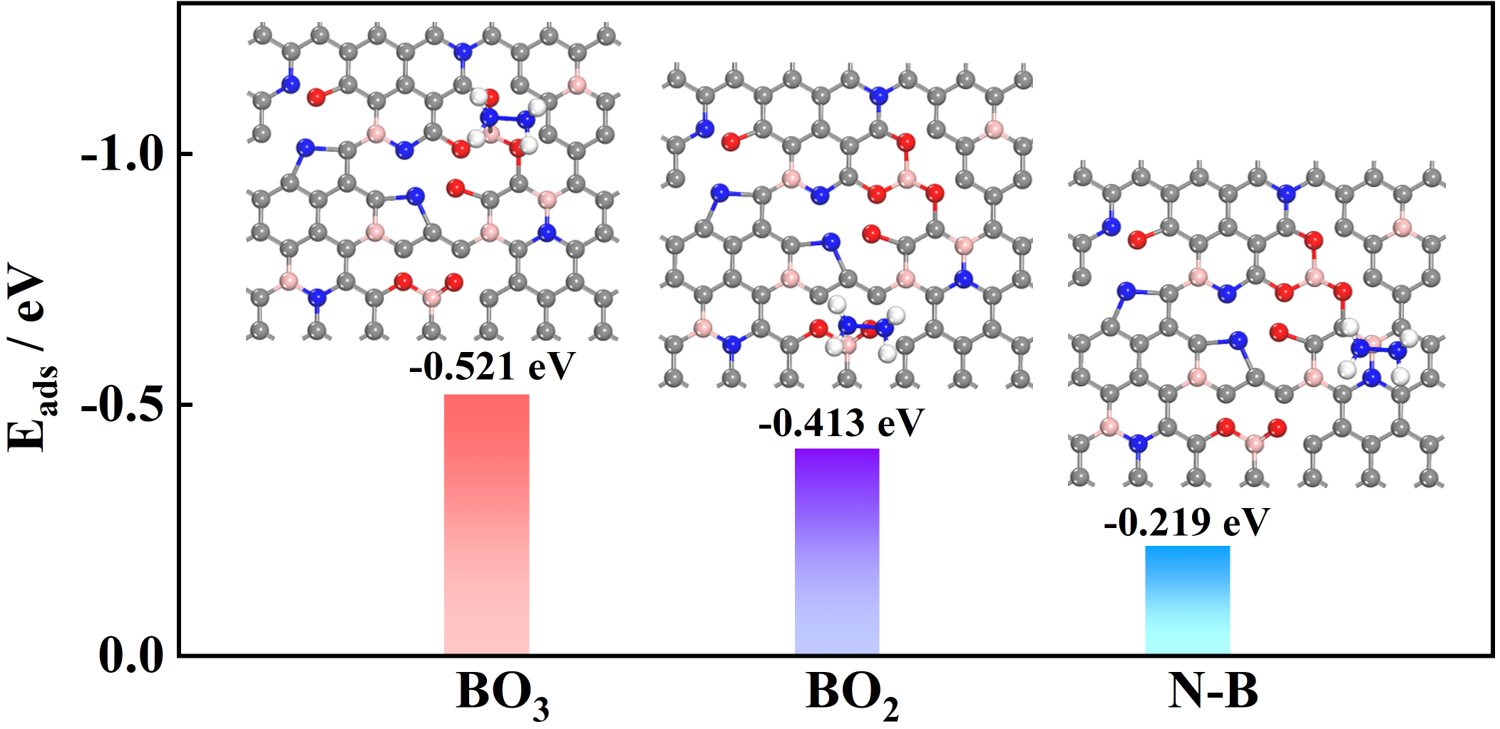


**Figure S42.** The adsorption energies of N_2_H_4_ on different adsorption sites of BNC.

1. **Geometric configurations**


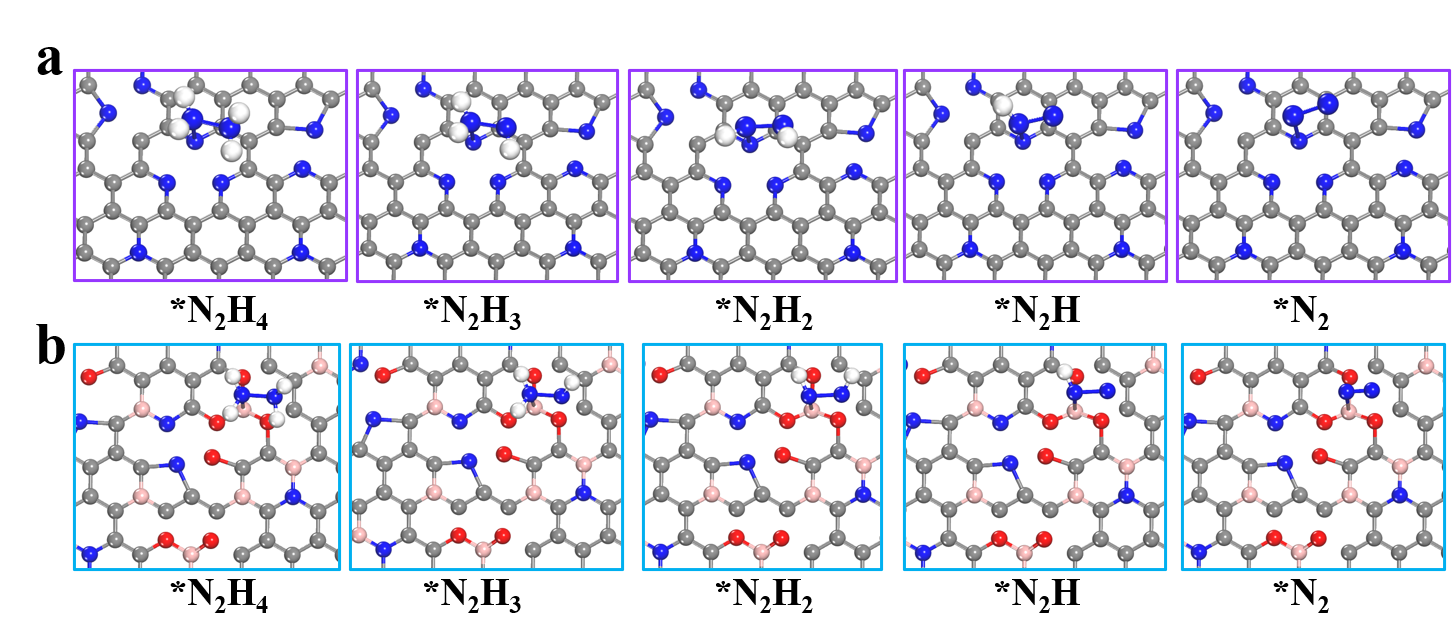


**Figure S43.** Geometric configurations. a, b, Optimized geometric configurations of various reaction intermediates along the reaction path of hydrazine oxidation on NC (a) and BNC (b).

1. **Electrocatalytic N_2_H_4_ performance**

**
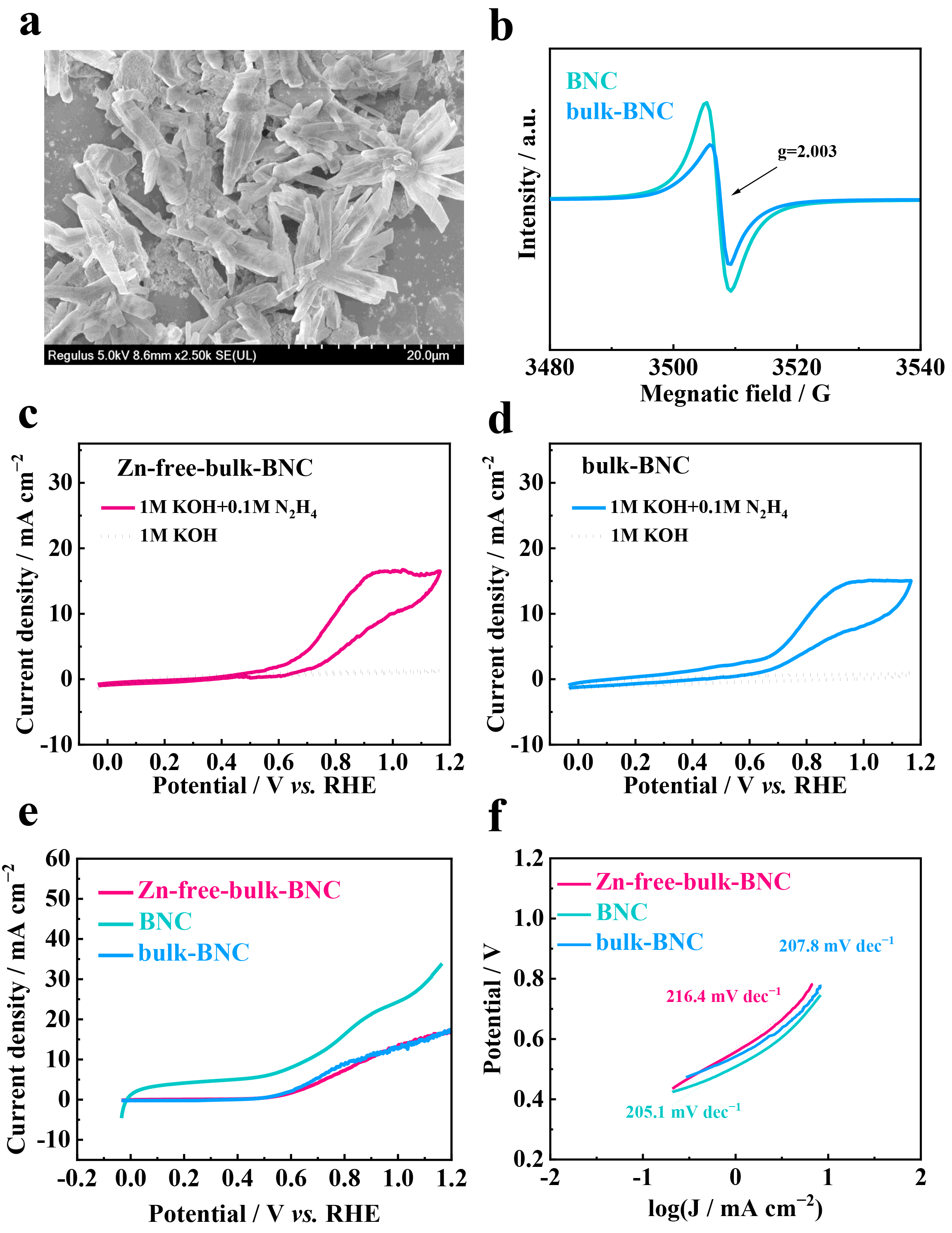
**

**Figure S44.** a, SEM image of bulk-BNC. b, EPR spectra of BNC and bulk-BNC. Electrocatalytic N_2_H_4_ performance. c, d, CV curves of Zn-free-bulk-BNC (c), bulk-BNC (d) electrodes in the 1 M KOH without or with 0.1 M N_2_H_4_ solution at a scan rate of 50 mV s^−1^. e, f, LSV curves (e) and the corresponding Tafel plots (f) of Zn-free-bulk-BNC, bulk-BNC and BNC electrodes in the 1 M KOH with 0.1 M N_2_H_4_ solution at a scan rate of 5 mV s^−1^.

1. **Stability of NC/BNC electrodes**


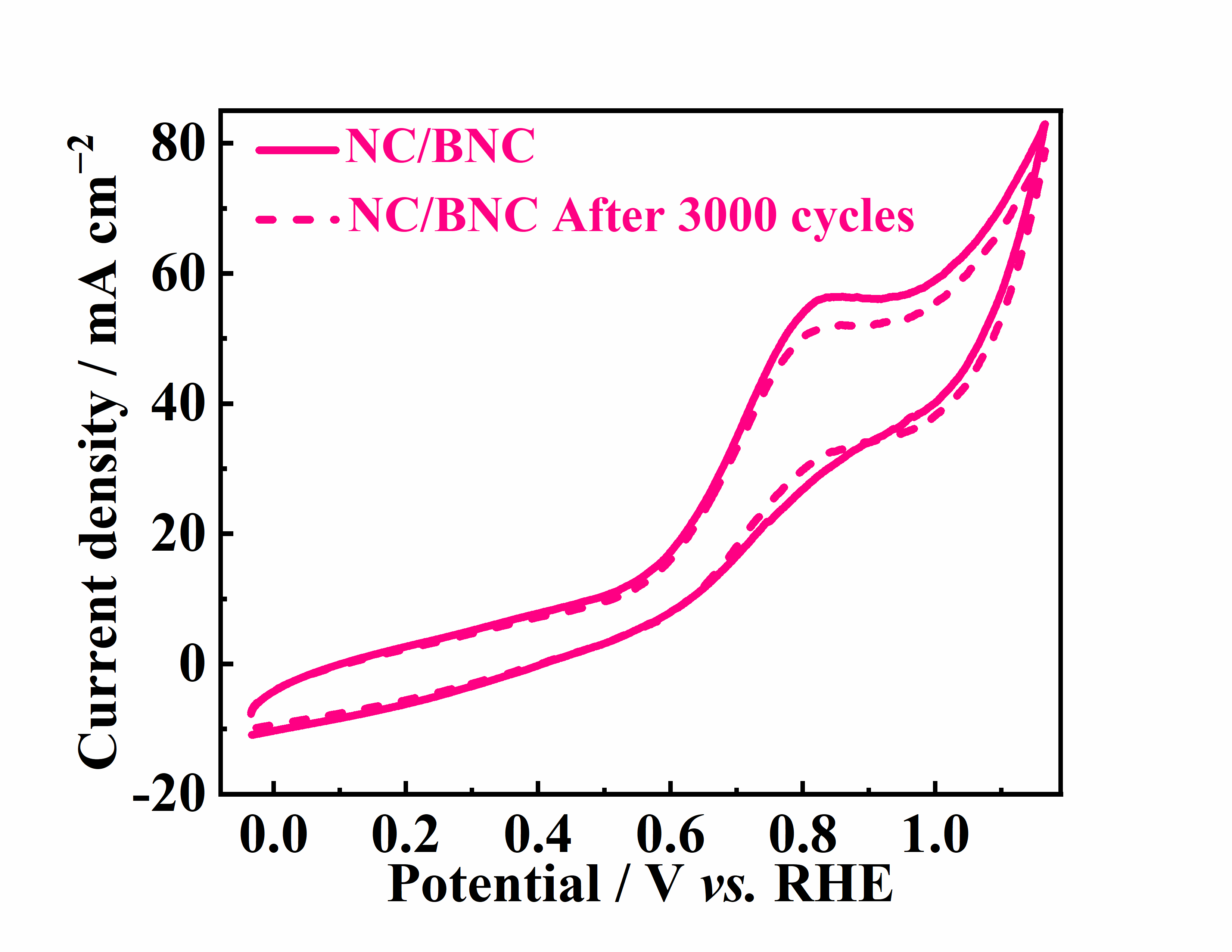


**Figure S45.** NC/BNC electrodes in 0.1 M N_2_H_4_ + 1M KOH solution at a scan rate of 50 mV s^−1^.

1. **Microstructure of NC/BNC after the stability test**


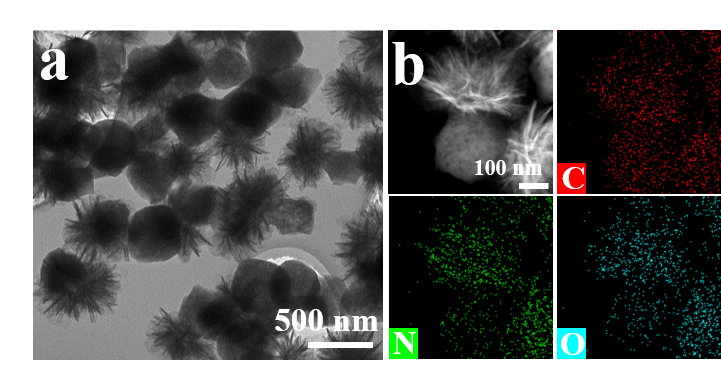


**Figure S46.** Microstructure of NC/BNC after the stability test. a, TEM image of NC/BNC after the stability test. b, HAADF-STEM image and corresponding elemental mapping images of NC/BNC after the stability test.

1. **XRD and XPS spectra of NC/BNC after the stability test**


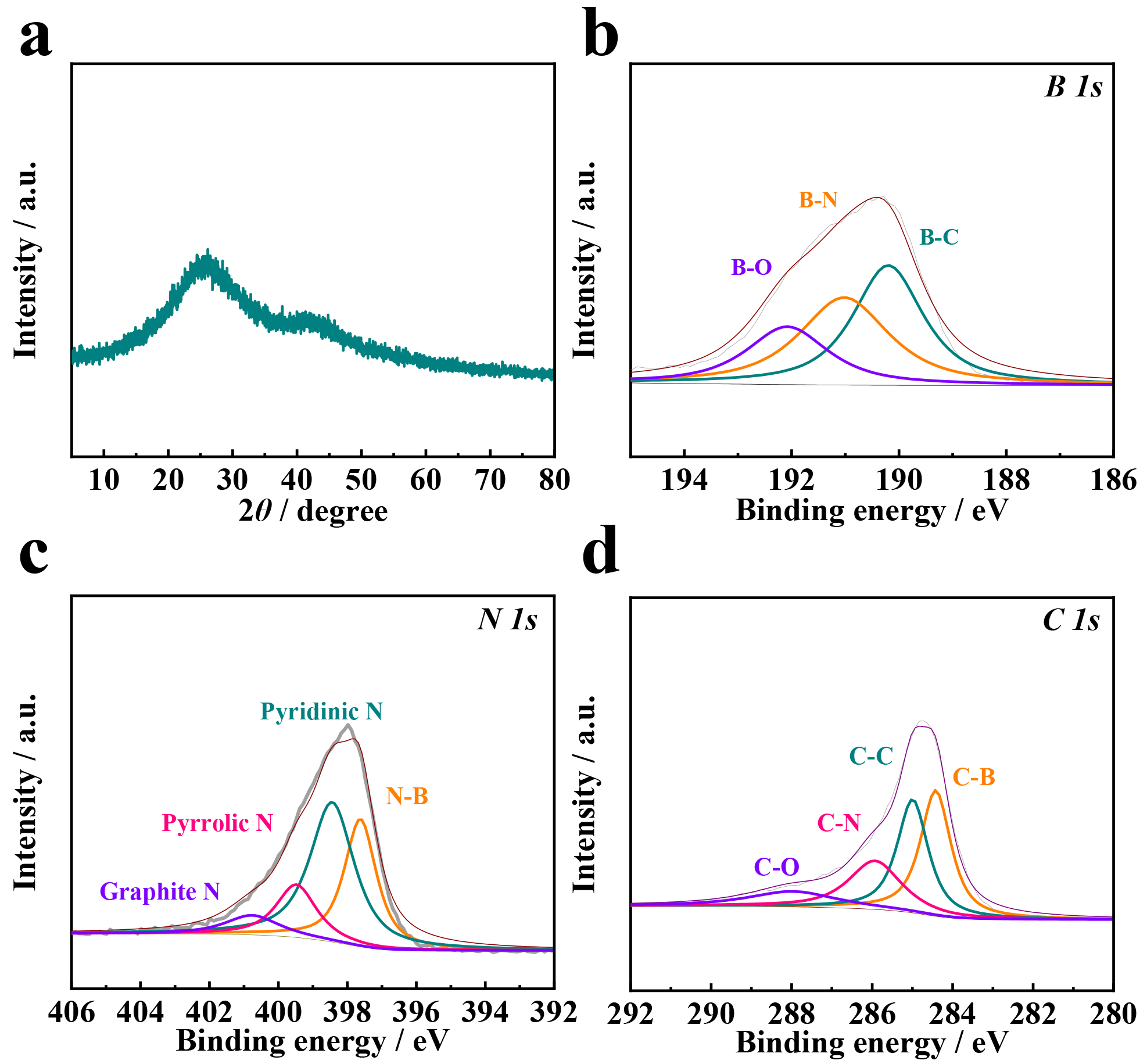


**Figure S47.** XRD and XPS characterizations. a, XRD pattern of NC/BNC after the stability test. b–d, XPS spectra of NC/BNC after the stability test.

1. **XANES spectra of NC/BNC after the stability test**


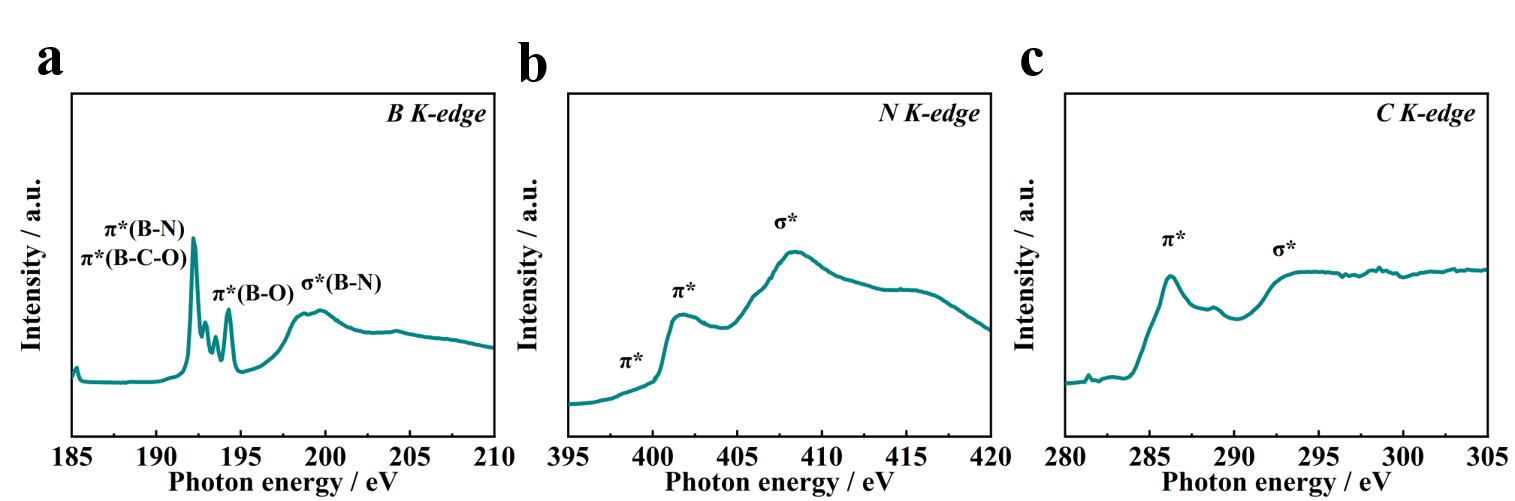


**Figure S48.** XANES characterizations. a–c, XANES spectra of B-K edge (a), N-K edge (b), and C-K edge (c) of NC/BNC after the stability test.

1. **Electrocatalytic OHzS performance**


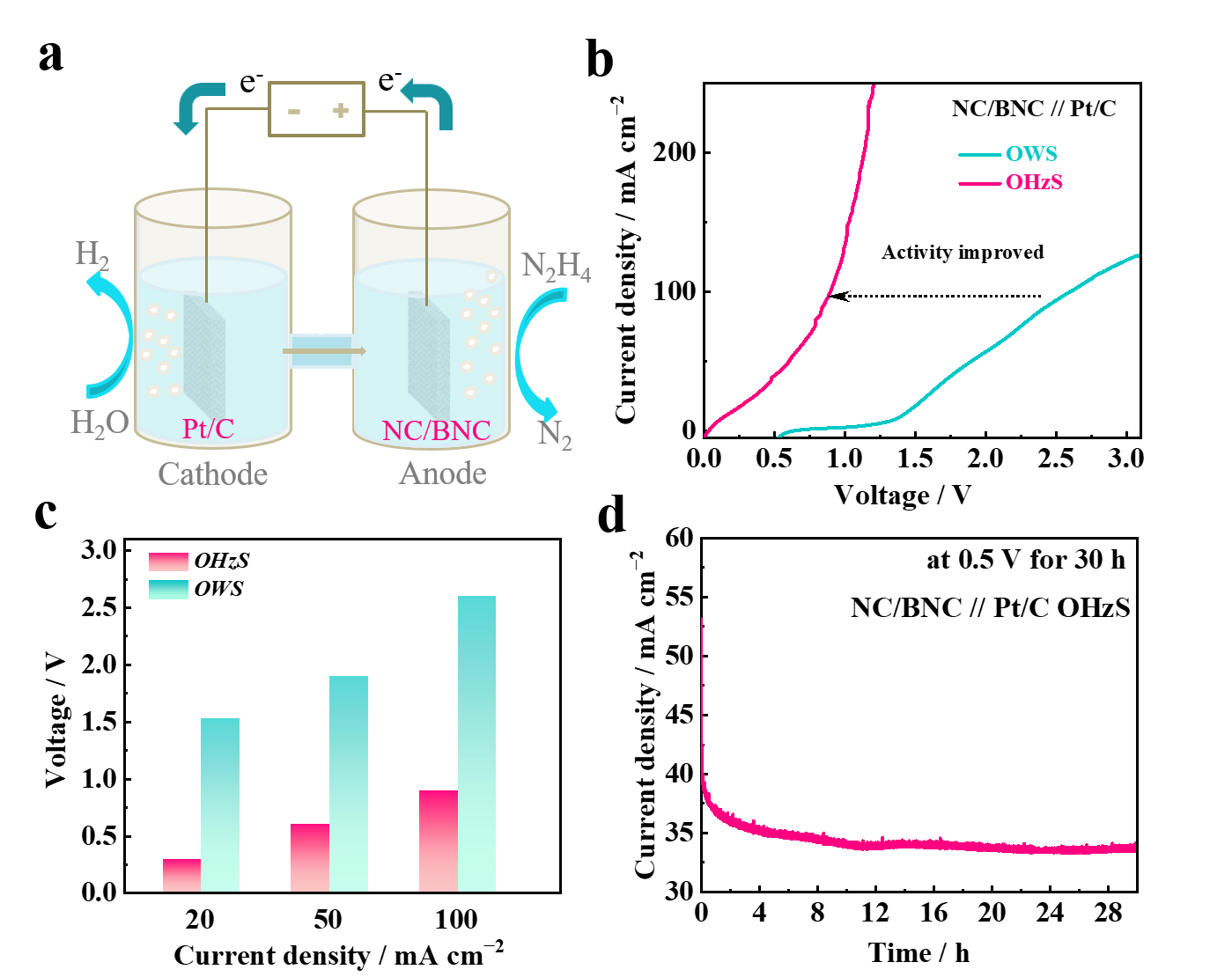


**Figure S49.** Electrocatalytic OHzS performance. a, The schematic illustration of the home-made hydrazine electrolyser. b, LSV curves and potentials for OWS and OHzS. c, Voltages at different current densities for OHzS and OWS. d, The chronoamperometry curve for OHzS recorded at the voltage of 0.5 V in 1.0 M KOH/0.1 M N_2_H_4_.

1. **Characterization results of catalysts**

**Table S1**. Characterization results of catalysts.

| Samples | S_BET_  /m^2^/g | S_Langmuir_  /m^2^/g | V_pore_  /cm^3^/g |
| --- | --- | --- | --- |
| NC | 720 | 1107 | 0.402 |
| NC/BNC | 233 | 736 | 0.578 |
| BNC | 69 | 252 | 0.194 |

1. **ICP-AES results for Zn and B, and elemental analysis data for C, N and H in NC, NC/BNC and BNC**

**Table S2****.** ICP-AES results for Zn and B, and elemental analysis data for C, N and H in NC, NC/BNC and BNC.

| Samples | Zn % | B % | C % | N % | H % |
| --- | --- | --- | --- | --- | --- |
| NC | 10.9 | 0.0 | 50.3 | 10.2 | 3.1 |
| NC/BNC | 6.5 | 6.8 | 45.6 | 14.1 | 3.0 |
| BNC | 3.3 | 14.6 | 36.7 | 19.1 | 2.1 |

1. **Comparison of the HzOR performances of NC/BNC with various reported electrocatalysts**

**Table S3.** Comparison of the HzOR performances of NC/BNC with various reported electrocatalysts.

| Samples | Electrolytes | Onset potentials  /mV | References |
| --- | --- | --- | --- |
| NC/BNC | 1M KOH +0.1 M N_2_H_4_ | 347 | *This Work* |
| S-C | 0.1 M PBS+50 mM N_2_H_4_ | 430 | *J. Mater. Chem. A 2017, 5, 1066-1077.* |
| NSC | 0.1 M PBS+50 mM N_2_H_4_ | 391 | *Appl. Catal. B Environ. 2018, 225, 30-39.* |
| N-O doped C | 0.1 M PBS+50 mM N_2_H_4_ | 390 | *Chem. Commun. 2016, 52, 13588-13591.* |
| Tree Bark NC | 1M KOH +0.1 M N_2_H_4_ | 400 | *Angew. Chem. Int. Ed. 2017, 56, 13513-13516.* |
| N-HPCM | 1M KOH +0.1 M N_2_H_4_ | 420 | *Small 16, 2020, 2002203.* |
| N-doped holey graphene | 0.1 M PBS+10 mM N_2_H_4_ | 507 | *Nanoscale 2013, 5, 3457-3464.* |
| N-doped C | 0.1 M PBS+5 mM N_2_H_4_ | 610 | *Electrochim. Acta 2014, 137, 693-699.* |
| NC  CoSe_2_  NiCoMoO_4_  Co_3_NPNAs/NF  V-Ni_3_N NS  Rh/N-CBs  Rh_2_S_3_/NC  FeN_4_/HPCM  Ni-NSA  Ni_3_S_2_ | 1M KOH +0.1 M N_2_H_4_  1M KOH+0.1M N_2_H_4_  1M KOH+0.3M N_2_H_4_  1M KOH+0.1M N_2_H_4_  1M KOH+0.1M N_2_H_4_  1.0 M KOH + 0.05 M N_2_H_4_  1.0 M KOH + 0.1 M N_2_H_4_  1.0 M KOH + 0.1 M N_2_H_4_  3 M KOH+1 M N_2_H_4_  1 M KOH+0.2 M N_2_H_4_ | 680  15  48  49  2  72  95  297  250  415 | *Angew. Chem. Int. Ed. 2018, 57, 17168-17172.*  *Angew. Chem. Int. Ed. 2018, 57, 7649-7653.*  *Small 2021, 17, 2008148.*  *Angew. Chem. Int. Ed. 2021, 60, 5984-5993.*  *ACS Appl. Mater. Interfaces 2021, 13, 3881-3890.*  *ACS Appl. Mater. Interfaces 2019, 11, 35039-35049.*  *Small Methods 2020, 4, 2000208.*  *Small 2020, 16, 2002203.*  *Angew. Chem. Int. Ed. 2016, 55, 693-697.*  *J. Mater. Chem. A 2018, 6, 19201-19209.* |

1. **Conductivity tests of the catalysts**

**Table S4.** Electrical conductivity of the catalysts.

| Samples | NC | NC/BNC | NC+BNC | BNC |
| --- | --- | --- | --- | --- |
| Conductivity S/mm | 52.8 | 38.6 | 20.9 | 15.4 |

1. **References**

S1. Kresse, G. & Furthmüller, J. Efficiency of ab-initio total energy calculations for metals and semiconductors using a plane-wave basis set. *Comput. Mater. Sci.* **6,** 15-50 (1996).

S2. Kresse, G. & Furthmüller, J. Efficient iterative schemes for ab initio total-energy calculations using a plane-wave basis set. *Phys. Rev. B* **54,** 11169-11186 (1996).

S3. Perdew, J. P., Burke, K. & Ernzerhof, M. Generalized gradient approximation made simple. *Phys. Rev. Lett.* **77,** 3865-3868 (1996).

S4. Kresse, G. & Joubert, D. From ultrasoft pseudopotentials to the projector augmented-wave method. *Phys. Rev. B* **59,** 1758-1775 (1999).

S5. Blöchl, P. E. Projector augmented-wave method. *Phys. Rev. B* **50,** 17953-17979 (1994).

S6. Grimme, S., et al. A consistent and accurate ab initio parametrization of density functional dispersion correction (DFT-D) for the 94 elements H-Pu. *J. Chem. Phys.* **132,** 154104 (2010).
